# Indolylamide Macrocyclization by a *Streptococcus pneumoniae* ThiF-like Enzyme Family Member

**DOI:** 10.1021/acs.orglett.5c01561

**Published:** 2025-05-21

**Authors:** Anshul Rajput, Keelie S. Butler, Daniel A. Springer, Jonathan R. Chekan

**Affiliations:** Department of Chemistry and Biochemistry, 14616 University of North Carolina at Greensboro, Greensboro, North Carolina 27412, United States

## Abstract

ThiF-like enzymes are present in diverse RiPP biosynthetic pathways and are known to catalyze reactions such as thiolactone and phosphoramidate bond formation. To uncover new chemical space for ThiF-like enzymes, we utilized a global genome mining approach and identified a minimal *ind* RiPP cluster in the human pathogen *Streptococcus pneumoniae*. In vitro characterization of IndF demonstrated the first indolylamide (Trp-Ile) linkage in a RiPP pathway and a new reaction type catalyzed by a ThiF-like enzyme.

## Graphical abstract

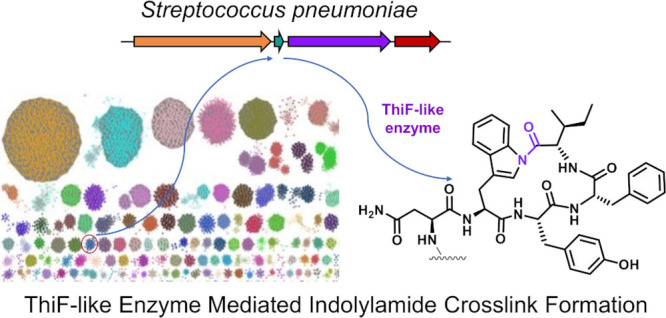

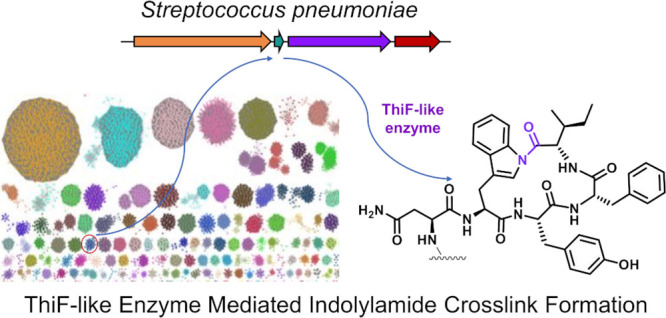

Ribosomally synthesized and post translationally modified peptides (RiPPs) represent a structurally diverse and rapidly growing class of natural products with a wide array of bioactivities.^1,2^ They originate from ribosomal synthesis but undergo further enzymatic post-translational modifications to generate over 40 distinct classes of RiPPs.^1^ The discovery of new RiPPs and their biosynthetic pathways has only accelerated in recent years due to advances in bioinformatic techniques,^3,4^ indicating the prevalence and significance of these molecules in nature.

As RiPPs have grown in number, different approaches have been used to find and classify them. Many RiPP families are defined by a characteristic structural feature, such as lanthipeptides which contain the class defining lanthionine linkage formed using one of five different pathways.^1,5^ In contrast, other well-known RiPPs exist outside this paradigm and are defined by the function of a single enzyme family that can perform diverse transformations, such as the P450s,^6^ radical SAMs (rSAMs/RiPP-RaSs),^7^ burpitides,^8,9^ and MNIOs.^10^ The ThiF-like enzyme family also appears to fall into this latter organization as they are operational in orthogonal RiPP biosynthetic pathways and responsible for a major modification, yet perform disparate reactions.

ThiF itself is present in the pathway for thiamine production,^11^ but related family members have been found in the biosynthesis of several RiPP natural products. For example, MccB is a ThiF-like family member present in the biosynthesis of microcin C7 (Figure 1), an antibiotic produced by *Escherichia coli*.^12^ This enzyme installs a phosphoramidate (N–P) linkage via ATP-dependent adenylation at the C-terminal asparagine in MccA.^13–15^ Similar chemistry was displayed by the microcin C analogue from *Bacillus amyloliquefaciens* which incorporates a carboxymethyl-cytidine moiety in place of C-terminal adenosine (Figure 1).^16^ Another ThiF-like enzyme, PaaA, is present in the biosynthesis of pantocin A (Figure 1), catalyzing its bicyclic core synthesis via an ATP-dependent intramolecular cyclization.^17^ More recently, a ThiF-like enzyme in *Streptococcus pneumoniae* (GrcB) was shown to install a C-terminal Glu-Cys thiolactone macrocycle that is then tailored by a RiPP-Ras family member (Figure 1).^18^ Finally, a targeted genome mining-based effort identified a new ThiF-like enzyme, EnfB, that is responsible for the production of a putative cyclic RiPP named enterofacecin (Figure 1) though its exact structure requires further detailed NMR studies.^19^ While the ThiF reaction products in each of these cases are seemly rather disparate, they are mechanistically similar. In each case, a ThiF-like enzyme adenylates a carboxylic acid using ATP that is then the target of attack by different nucleophiles to yield the observed scaffolds (Figure 1A).

**Figure 1:**
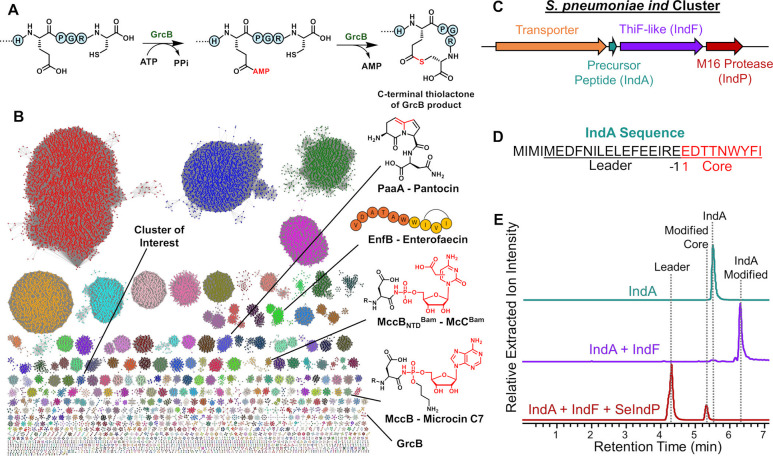
(A) The ThiF-like enzyme GrcB catalyzes cross-link formation by utilizing ATP to generate an adenylated intermediate. (B) Sequence similarity network of ThiFs with RRE motifs. SSN was generated using an e-value of 90 and displayed as a 90% rep network. Documented ThiF-like enzyme RiPP reactions are indicated. (C) *Ind* biosynthetic gene cluster from *S. pneumoniae*. (D) Annotated IndA precursor peptide sequence. (E) UHPLC-HRMS (Ultra-High Performance Liquid Chromatography-High Resolution Mass Spectrometry) analysis of enzymatic assays. Extracted ion chromatograms for IndA alone (top trace), IndF activity assay (middle trace), and SeIndP proteolysis assay (bottom trace); (IndA [M+2H]^+2^: 1613.7319 *m*/*z*, IndA-Modified [M+2H]^+2^: 1604.7229 *m*/*z*, Leader [M+2H]^+2^: 1028.9822 *m*/*z*, Modified Core [M+2H]^+2^: 585.7591 m*/z*) using a mass tolerance of 20 ppm.

The findings in the past three decades along with a recent bioinformatic analysis of lactic acid bacteria imply that many more ThiF-like enzymes remain to be discovered.^19^ To explore this hypothesis, we employed a global bioinformatic approach by scanning the entirety of the bacterial protein RefSeq database (>326 million proteins) for the presence of ThiF-family members (PF00899) using the publicly available hidden Markov model (HMM). The results of this yielded a list of 172,313 bacterial ThiF-like proteins, the majority of which were unrelated to RiPP biosynthesis. To limit our search to candidates only found in RiPP biosynthetic clusters, we focused on using a RiPP Recognition Element (RRE)-based genome mining strategy. RREs are often present fused to catalytic domains in RiPP biosynthetic clusters and bind to the leader peptide using a conserved structural architecture.^20^ Therefore, we reasoned that ThiF-like enzymes fused to RREs may be likely candidates for RiPP biosynthetic enzymes. Filtering the identified ThiF-like enzymes for the presence of RREs using the software RRE-Finder^21^ in exploratory mode identified 27,400 candidate RRE-ThiF fusions. The results were then analyzed by creating a sequence similarity network (SSN) using the Enzyme Function Initiative-Enzyme Similarity Tool (EFI-EST)^22,23^ and annotated with the previously documented ThiF chemistry (microcin C7, pantocin, GrcB, and enterofaecin). The remaining, unannotated SSN clusters were then analyzed for the presence of novel ThiF-containing RiPP-like gene clusters (Figure 1B). Specifically, they were searched for the presence of conserved precursor peptide-like genes, proteases, and a transporter. Moreover, clusters with cyclodehydratases (YcaOs) we excluded as ThiFs in these systems have been shown to serve only as docking domains.^24^ While several SSN clusters appeared to fit this criteria to potentially encode new RiPP chemistry (Figure S1), we focused on a cluster rich in Streptococci significant to human health. In this SSN grouping we noted a biosynthetic gene cluster from *S. pneumoniae*, an important opportunistic pathogen responsible for the majority of bacterial pneumonia cases (Figure 1C). This *ind* cluster encodes a precursor peptide (IndA), a ThiF-like enzyme (IndF), a peptidase (IndP), and a transporter.

In order to characterize the IndF-catalyzed reaction, we utilized an in vitro biochemical approach. IndF was heterologously expressed as a maltose binding protein (MBP) fusion construct in *E. coli*, followed by purification by Ni-NTA and size exclusion chromatography (Figure S2). The IndA precursor peptide that lacked the N-terminal MIMI amino acid sequence was synthesized in parallel using solid-phase peptide synthesis (SPPS) (Figure 1D). Upon incubation of purified MBP-IndF (10 μM) with substrate IndA (100 μM), 4 mM MgCl_2_, 0.5 mM ATP in 20 mM HEPES-buffer (pH 7.5) at room temperature for 18 h, we observed a new mass of 1604.7229 *m*/*z* [M+2H]^+2^ corresponding to a loss of one H_2_O molecule compared to the control of 1613.7319 *m*/*z* [M+2H]^+2^ (Figures 1E, S3 and S4). Consistent with the ATP-dependent function of ThiF-like enzymes, assays performed without ATP did not yield any mass change (Figure S5). Further MS/MS analysis revealed that the modification was localized to the C-terminus of IndA (Figures S3 and S4).

We next tried to assess the activity of the protease in the cluster, SpIndP. This enzyme was annotated as a zinc-dependent M16 protease family member (PF00675). Proteases in this group are composed of between two and four structurally related domains either as a single polypeptide or formed as part of a dimeric complex.^25^ Surprisingly, SpIndP appeared to exist as an atypical single domain protein (Figure S6) and heterologous expression of the SpIndP either with a His_6_ tag or MBP-fusion in *E. coli* produced no observable protein. Examination of homologous proteases in related clusters indicated that other IndPs appeared to be a more typical two domain M16 protease (Figure S6). Indeed, the IndP from *Streptococcus equi* (SeIndP) was successfully expressed in *E. coli*. The predicted precursor peptide in the *S. equi* cluster closely matched the *S. pneumoniae* IndA precursor peptide (Figure S6), suggesting it could serve as a replacement. In vitro incubation of SeIndP with IndF and IndA indicated cleavage into a leader (1028.9822 *m*/*z* [M+2H]^+2^) and a modified core (585.7591 *m*/*z* [M+2H]^+2^) composed of a EDTTNWYFI sequence (Figures 1D, 1E, and S7–S9). While SeIndP could also cleave the linear IndA peptide, it did so at a much lower rate than the modified IndA (Figure S10). MS/MS analysis of the modified core indicated unusual fragmentation wherein the Y7 and F8 amino acids could be fragmented from the modified product, but not in the linear IndA. This seemed to suggest that a cyclization occurred between W6 and I9 (Figure S11). It should be noted that the amino acids are numbered according to conventional RiPP leader-core nomenclature (Figure 1D).

To definitively locate the position and linkage of the IndF-catalyzed cyclization, extensive structure elucidation was conducted with multidimensional-NMR spectroscopy. For this purpose, we scaled up the in vitro assays and the resulting modified IndA was cleaved with α-lytic protease to yield a shortened five amino acid core peptide fragment amenable for structure determination named IndA-Cyclic_N5–I9_ (Figure S12). The crude modified product was purified by flash chromatography, resulting in 2.5 mg of pure product. Separately, the corresponding linear 5-mer peptide (NWYFI) was synthesized in parallel by SPPS as IndA_N5–I9_ (Figure S13). Both IndA_N5–I9_ and IndA-Cyclic_N5–I9_ were subjected to extensive NMR analysis and compared. In the IndA_N5–I9_ spectra, the indole N–H signal appeared at 10.75 ppm as doublet while in IndA-Cyclic_N5–I9_ the indole N–H signal is not present (Figure 2A and Supporting Information). Furthermore, the Trp-H_2_ signal at 7.06 ppm is a doublet, while in IndA-Cyclic_N5–I9_ it appears at 7.56 ppm as a singlet (Figure 2A, B, and C). Combined with the MS/MS data, these results indicate loss of the indole NH proton in the modified peptide and formation of an isopeptide bond with C-terminal Ile. Further analysis using 2D-ROESY was conducted to validate this hypothesis. Indeed, ROESY NMR of IndA-Cyclic_N5–I9_ revealed the connection of Trp-H_2_ (7.56, s) with Ile-H_α_ (4.21, dd) and Ile-H_β_ (2.21, s), both of which were absent in IndA_N5–I9_ (Figure 2B and C). These connections confirmed the formation of an indolylamide isopeptide bond (Figure 2D). Finally, Marfey’s analysis showed an all l-amino acid stereochemistry in the final cyclic product (Supporting Information). To further validate this W6 to I9 linkage, an W6A variant of IndA was synthesized. This peptide showed no conversion to product indicating the importance of W6 in forming the cyclic product (Figures S14–17).

**Figure 2:**
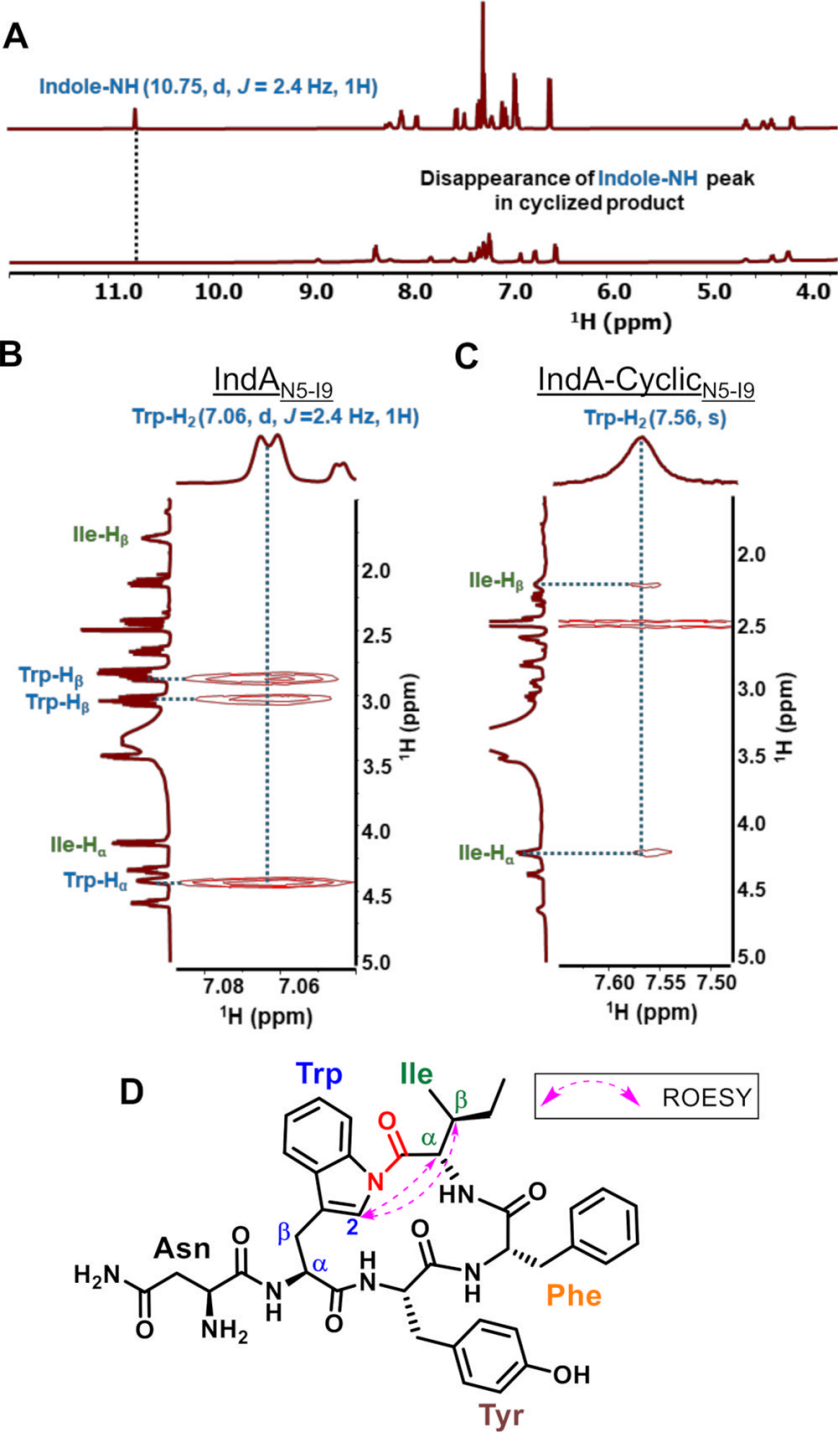
(A) Comparison of ^1^H NMR spectra for linear (Top) and cyclized (Bottom) IndA_N5-I9_ demonstrates the disappearance of the indole-NH proton (Trp-NH) peak in the cyclized product. (B) The ^1^H–^1^H ROESY spectra for IndA_N5–I9_ demonstrates correlations between the indole-2 proton (Trp-H_2_) and the Trp-H_β_ and Trp-H_α_. (C) The ^1^H–^1^H ROESY spectra for IndA-Cyclic_N5–I9_ shows correlations between the indole-2 proton (Trp-H_2_) and the Ile α-proton and Ile β-proton. (D) Selected key ROESY correlation for IndA-Cyclic_N5–I9_.

Due to the unprecedented indolylamide formation by a ThiF-like enzyme, we sought to explore some of the interactions between IndF and IndA. AlphaFold 3 ^26^ was used to create models of the IndF enzyme with the IndA peptide, ATP, and magnesium ion docked into the active site. While modeling of an IndF:IndA monomer did not yield a productive interaction, modeling of the IndF dimer with IndA appeared to be relevant. This observation is consistent with the dimeric crystal structures of both PaaA and MccB.^13,15,17^ Moreover, the predicted fold of IndF was largely consistent with the experimentally determined structures of PaaA and MccB with RMSDs of 3.44 Å and 4.34 Å, respectively (Figures S18 and S19), despite low sequence similarity (Figure S20). Curiously, examination of the IndF oligomeric structure by size exclusion chromatography suggested it to be present in monomeric and dimeric forms, potentially indicating a dynamic structural state (Figure S21).

The model indicated that the IndA substrates were shared across the dimer, with IndA binding to the RRE of one monomer and the C-terminus threading into the active site of the other (Figure 3A). Examination of the active site revealed that the C-terminal I9 carboxylic acid is positioned for adenylation by ATP, consistent with the known mechanism of ThiF-like enzymes (Figure 3B).^13^ Indeed, replacement of ATP with the α-β nonhydrolyzable analogue AMPCPP in enzyme assays abolished turnover while the β-γ nonhydrolyzable AMPPCP analogue still enabled activity (Figure S5). The AlphaFold 3 model also suggested residues important for IndA and ATP binding. Amino acids K256, Y228, and T298 were each located near the IndA core. K256 and Y228 were found approximately 6 Å away from W6 of IndA and T298 was observed within 4 Å from I9 of IndA. Alanine mutations in each of these three residues led to a substantial loss of activity (Figure 3C). The ATP binding interactions were also evaluated. Specifically, K179 and Q167 were found within hydrogen bonding distance of the α-phosphate of ATP. Mutation of this lysine to alanine nearly abolished activity while the Q167A variant had a minor decrease in activity compared to wild-type (WT) IndF. Further analysis of IndF models compared to the solved crystal structures of PaaA and MccB revealed conserved residues in the ATP binding site, as predicted (Figures S22 and S23). Beyond the ATP binding motif of the active site, there were not high levels of conservation with PaaA and MccB, which precluded detailed comparisons.

**Figure 3:**
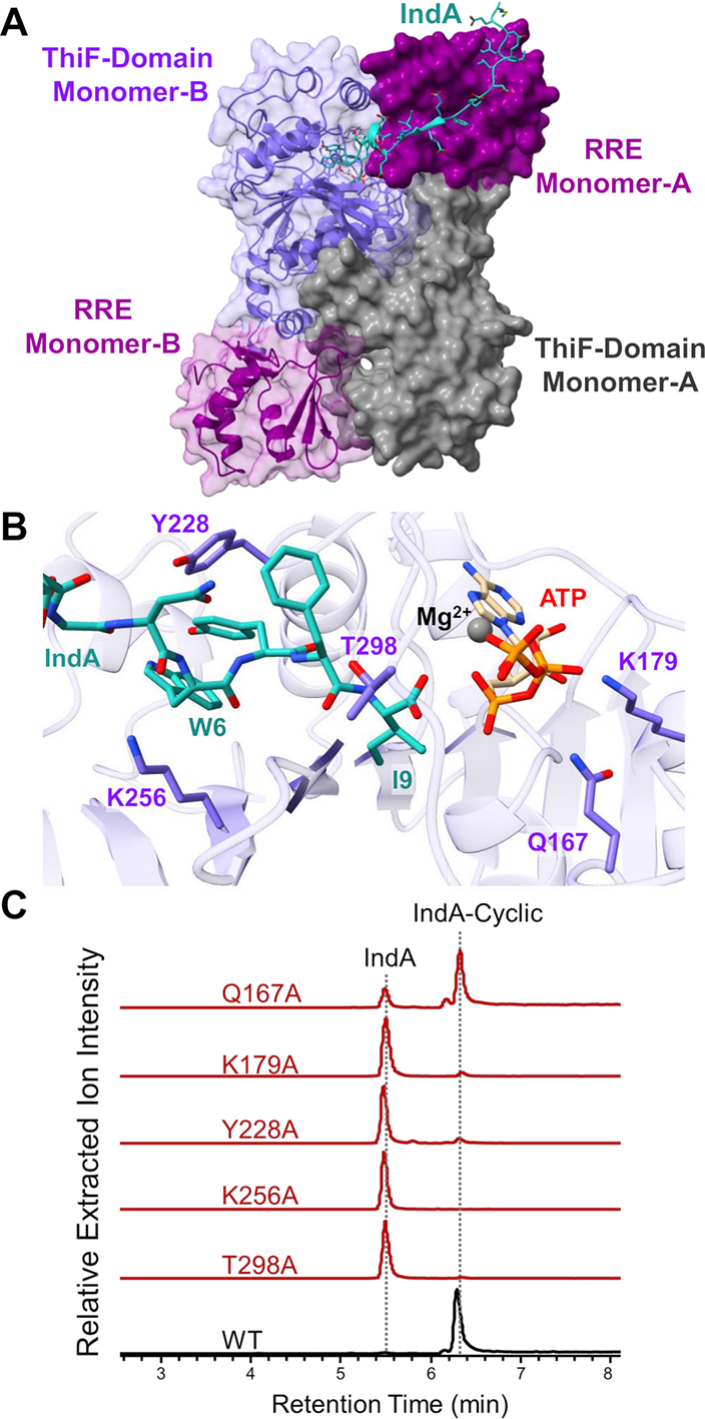
(A) AlphaFold 3 model of the dimeric IndA, IndF, ATP, and Mg^2+^ structure. (B) AlphaFold 3 model of the IndF active site with important residues labeled. (C) Extracted ion chromatograms for IndF mutant assays (IndA substrate [M+2H]^+2^: 1613.7319 *m*/*z* and IndA-Cyclic product [M+2H]^+2^: 1604.7229 *m*/*z*) using a mass tolerance of 20 ppm.

As the indole N1 is not very nucleophilic, it likely requires activation and deprotonation for successful attack of the adenylated intermediate. Recently studies on the indolylamide forming bulferamide thioesterase (BulbE TE) suggested that a His present in active site is responsible for activation of the indole.^27^ Based on our modeling and mutagenesis studies, K256 or Y228 may fulfill this role in promoting nucleophilic attack by Trp-NH. However, our modeling shows the substrate complex. The formation of the adenylated intermediate is likely to induce a rearrangement in the active site to position I9 closer to W9 than the ∼10 Å observed in the current model. Therefore, other residues may be mechanistically important as well.

Ultimately, our global genome mining approach to discover new ThiF-like enzyme chemistry in RiPP biosynthetic pathways uncovered a new modification: macrocyclization through an indolylamide cross-link. This motif is unprecedented in RiPP natural product pathways to our knowledge. Outside of RiPPs, this reaction has only been observed as part of NRPS pathways, such as in bulbiferamide and psychrophilin.^27–30^ In the case of bulbiferamide, this reaction is catalyzed by a specialized *N*-acylindole-forming thioesterase.^27,31^ In addition to the new RiPP scaffold, the presence of this putative natural product may lead to important insights into the pathogenesis of clinically relevant Streptococci. While we are actively working to validate the presence of this indolylamide containing cyclic peptide in *S. pneumoniae* and uncover its physiological function, the cluster’s presence near multiple antibiotic resistance genes and conjugal elements suggests relevance to pathogenesis (Figure S24). Moreover, the prevalence of diverse ThiF-like enzymes across Streptococci (Figure S25) indicates the importance of these pathways and the potential to uncover new RiPP modifications.

## Supplementary Material



## Data Availability

The data underlying this study are available in the published article and its Supporting Information.

## Notes

Description of materials and methods used in this study, results of enzyme assays, MS/MS fragmentation analysis, and peptide structure elucidation (PDF)
