# Supplementary material for: Indolylamide Macrocyclization by a Streptococcus pneumoniae ThiF-like Enzyme Family Member
Source: Org Lett. 2025 May 21;27(22):5765–70. doi: 10.1021/acs.orglett.5c01561 (PMC12150305; doi:10.1021/acs.orglett.5c01561)
Supplement: Supplementary file 1 [file ol5c01561_si_001.pdf]

SUPPLEMENTAL INFORMATION FOR:

Indolylamide Macrocyclization by a *Streptococcus pneumoniae* ThiF-Like Enzyme Family Member

Anshul Rajput‡, Keelie S. Butler‡, Daniel A. Springer, Jonathan R. Chekan*

Department of Chemistry and Biochemistry, University of North Carolina at Greensboro, NC 27402, USA

*Correspondence jrchekan@uncg.edu

Table of Contents

Materials and Methods 4

Cloning *IndF* 4

Protein Expression and Purification 4

Site Directed Mutagenesis of IndF 5

In vitro Enzymatic Assays 6

Size Exclusion Analysis: Gel filtration standard curve 6

UHPLC-HRMS Analysis 6

Solid-phase Peptide Synthesis of IndA 7

Purification of linear $IndA_{N5-I9}$ and $IndA\text{-}Cyclic_{N5-I9}$ 8

NMR Analysis of $IndA_{N5-I9}$ and $IndA\text{-}Cyclic_{N5-I9}$ 8

Marfey's Analysis 9

Supplementary Figures and Tables 10

Table S1. Accession codes and protein sequences 10

Figure S1. Examples of other biosynthetic gene clusters containing ThiF-like enzymes from sequence similarity network. 12

Figure S2. Heterologous expression of *ind* proteins 13

Figure S3. MS/MS spectrum for IndA. 14

Figure S4. MS/MS spectrum for IndA-Cyclic after incubation with IndF. 15

Figure S5. UHPLC-HRMS analysis of IndF assays with varied cofactors/co-substrates. ... 16

Figure S6. *S. equi* and *S.pneumoniae* protease alignment: 17

Figure S7. MS/MS spectrum for cleaved IndA leader after incubation with SeIndP. 18

Figure S8. MS/MS spectrum for cleaved IndA core after incubation with SeIndP. 19

Figure S9. MS/MS spectrum for cleaved IndA-Cyclic core after incubation with IndF and SeIndP. 20

Figure S10. UHPLC-HRMS analysis of SeIndP cleavage of leader peptide from IndA 21

Figure S11. MS/MS fragmentation of IndA-Cyclic core, supporting W6 to I9 cyclization. ... 22

Figure S12. MS/MS spectrum of $IndA\text{-}Cyclic_{N5-I9}$ after cleavage by α-lytic protease. 23

Figure S13. MS/MS spectrum of synthetic $IndA_{N5-I9}$. 24

Figure S14. UHPLC-HRMS analysis of IndF cyclization of IndA variants. 25

Figure S15. MS/MS spectrum of IndA Met(-16)Nle. 26

Figure S16. MS/MS spectrum of IndA-Cyclic Met(-16)Nle 27

Figure S17. MS/MS spectrum of IndA Met(-16)Nle+W6A. 28

Figure S18. PaaA and IndF structure comparison 29

Figure S19. MccB and IndF Structure comparison: 30

Figure S20. Sequence alignment of IndF, EnfB, MccB, PaaA, and GrcB. 31
Figure S21. Linear calibration plot for HiLoad 16/600 Superdex S200 size exclusion chromatography column 32
Figure S22. Structure overlay of PaaA and IndF ATP Binding Domains..........................33
Figure S23. Structure overlay of MccB and IndF ATP Binding Domains 34
Figure S24. Genomic context for IndF biosynthetic gene cluster (GenBank assembly CP002176.1). 36
Figure S25. Sequence Similarity Network with Streptococcus ThiF-like genes..........................37
Table S2. NMR table for $IndA_{N5-I9}$ 38
Figure S26. ^{1}H-NMR spectrum of $IndA_{N5-I9}$ in DMSO-d_6 (500 MHz). 40
Figure S27. ^{1}H-^{1}H-COSY NMR spectrum of $IndA_{N5-I9}$ in DMSO-d_6 (500 MHz). 41
Figure S28. ^{1}H-^{13}C-HSQC NMR spectrum of $IndA_{N5-I9}$ in DMSO- d_6 (500 MHz). 42
Figure S29. ^{1}H-^{13}C-HMBC NMR spectrum of $IndA_{N5-I9}$ in DMSO- d_6 (500 MHz). 43
Figure S30. ^{1}H-^{1}H-ROESY NMR spectrum of $IndA_{N5-I9}$ in DMSO- d_6 (500 MHz). 44
Table S3. NMR table for IndA-$Cyclic_{N5-I9}$..........................45
Figure S31. ^{1}H-NMR spectrum of IndA-$Cyclic_{N5-I9}$ in DMSO-d_6 (500 MHz). 47
Figure S32. ^{1}H-^{1}H-COSY NMR spectrum of IndA-$Cyclic_{N5-I9}$ in DMSO-d_6 (500 MHz). 48
Figure S33. ^{1}H-^{13}C-HSQC NMR spectrum of IndA-$Cyclic_{N5-I9}$ in DMSO-d_6 (500 MHz). 49
Figure S34. ^{1}H-^{13}C-HMBC NMR spectrum of IndA-$Cyclic_{N5-I9}$ in DMSO- d_6 (500 MHz). 50
Figure S35. ^{1}H-^{1}H-ROESY NMR spectrum of IndA-$Cyclic_{N5-I9}$ in DMSO- d_6 (500 MHz). ...51
Figure S36. Extracted ion chromatogram of Marfey's analysis for Asn 52
Figure S37. Extracted ion chromatogram of Marfey's analysis for Trp 53
Figure S38. Extracted ion chromatogram of Marfey's analysis for Tyr 54
Figure S39. Extracted ion chromatogram of Marfey's analysis for Phe 55
Figure S40. Extracted ion chromatogram of Marfey's analysis for Ile..........................56
Supplementary References..........................57

Materials and Methods

Cloning *IndF*

E. coli codon optimized *Streptococcus pneumoniae* ThiF (IndF) gene was commercially purchased from Twist Biosciences (WP_000371017.1) and cloned into maltose binding protein, MBP-pET28, vector for protein expression. Primers were ordered and synthesized by Integrated DNA Technologies (IDT). For construction of the MBP-pET28-IndF plasmid, primers were designed with a 21-nucleotide overhang as follows:

Forward: 5'-CTGTACTTCCAATCCGGATCCATGGACAATTACTTTCCCAAATGGAATCAAGACC -3'

Reverse: 5'-GGTGGTGGTGGTGCTCGAGTTACTCATCAATTTTCAAGATCTCCCACAGCTTAC -3'

The PCR product was then used in Gibson Assembly (New England Biolabs) standard procedure. The constructed vector was transformed into competent DH-10B *E. coli* cells and several colonies were grown overnight at 37 °C at 200 RPM in LB broth with 50 μg mL^{-1} kanamycin. Plasmids were purified using Takara Bio USA, Inc. NucleoSpin® Plasmid purification kit and sequenced by Plasmidsaurus, while the remainder was stored at -20°C. The plasmid was subsequently transformed into competent BL-21 *E. coli* cells and plated on LB media with 50 μg mL^{-1} kanamycin.

Protein Expression and Purification

E. coli BL-21 was used for the heterologous protein expression of *S. pneumoniae* ThiF-like enzyme (IndF) using MBP-pET28-IndF vector cloned into *E. coli* cells. Terrific Broth (TB) supplemented with 4 mL of glycerol and was used to grow *E. coli* cells. Cultures were grown at 37 °C and 200 rpm with antibiotic for selection (kanamycin 50 μg mL^{-1}) for several hours until an OD_{600} of 0.6-0.8 was reached. The temperature of the incubator was then decreased to 18 °C and cells were grown for an additional 1 hour. After cooling, 1 mM isopropyl β-D-1-thiogalactopyranoside (IPTG) was added to initiate protein expression and cultures were grown overnight. Cell cultures were centrifuged at 4000 × g for 30 minutes at 4 °C in Sorvall X Pro Series swinging bucket centrifuge. Cell pellet was resuspended in 40 mL of suspension buffer (20 mM HEPES, 300 mM NaCl, 10% glycerol, pH 7.5). Sonication (Branson Sonifier®) was used to lyse pelleted cells using 80% attenuation in 1-minute intervals, with 15 sec of sonication and 45 sec of rest for a total of 6 minutes of total active sonication time. Cells were centrifuged (20000 × g for 45 minutes at 4°C) and supernatant was loaded into 5 mL HiTrap™ Ni charged column (Cytiva) and washed with buffer A (30 mM imidazole, 20 mM Tris, 1 M NaCl, pH 8.0). Protein was eluted using FPLC (Cytiva Akta Go) and a linear gradient of 30-250 mM imidazole using buffer A and

buffer B (250 mM imidazole, 20 mM Tris, 1M NaCl, pH 8.0) over a 20-minute period, collecting 5 mL fractions. Presence of protein was confirmed with SDS-PAGE. Fractions containing protein were combined and concentrated using 30 kDa Amicon Ultra Centrifugal Filters (Millipore) at 4000 × g. Concentration fractions were run through HiLoad™ 16/600 Superdex 200 prep grade gel filtration column (Cytiva) and eluted in 3 mL fractions using buffer C (20 mM HEPES, 300 mM NaCl, 10% glycerol, pH 8.0). Fractions that contained protein were concentrated using 30 kDa Amicon Ultra Centrifugal Filters (Millipore) at 4000 × g, confirmed by SDS page, quantified using Qubit Broad Range Protein Kit and stored at -80 °C. Attempts at expressing M16 protease in the gene cluster from *S. pneumoniae* (SpIndP) were unsuccessful. A homologous codon optimized protease from a gene cluster in *Streptococcus equi* (SeIndP) was purchased from Twist Biosciences (WP_001034678.1) in pET28 vector and heterologous expression was successful.

Attempts to express the *S. pneumoniae* protease using MBP-pet28 vector were made using the following primers:

SpIndF MBP FWD: 5’-CTGTACTTCCAATCCGGATCC AACAAGAAGATCATTTTCAA-3’

SpIndP MBP REV: 5’-GGTGGTGGTGGTGCTCGAG TCAAATTTTAATAGAGTTCTTAATATA-3’

Site Directed Mutagenesis of IndF

IndF mutants were created using PCR mutagenesis with primers designed using Aligent QuikChange® Primer Design.[1] Primers were synthesized by Integrated DNA Technologies.

K179A_Forward: 5’-TGTTCACTTTCAACGACATTGGCAAGTATGCAGTGGACTGTGTGAA-3’

K179A_Reverse: 5’- TTCACACAGTCCACTGCATACTTGCCAATGTCGTTGAAAGTGAACA-3’

Q167A_Forward: 5’- CAAAGAAAACAACATCAACCGTGCGTCAATGTTCACTTTCAACGAC-3’

Q167A_Reverse: 5'- GTCGTTGAAAGTGAACATTGACGCACGGTTGATGTTGTTTTCTTTG-3'

Y228A_Forward: 5'-GCATGGACGAACCCCCTGCTATCGCACAGCGCTTAG-3'

Y228A_Reverse: 5'-CTAAGCGCTGTGCGATAGCAGGGGGTTCGTCCATGC-3’

K256A_Forward: 5'-CCCAGCGCTCAGCCGGGGCACTGCTTTTCTGTAATCC-3'

K256A_Reverse: 5'-GGATTACAGAAAAGCAGTGCCCCGGCTGAGCGCTGGG-3

T298A_Forward: 5'-ATGATGGAAAACTGATCGCCGCCAACATCCTGACG-3'

T298A_Reverse: 5'-CGTCAGGATGTTGGCGGCGATCAGTTTTCCATCAT-3'

PCR products were transformed into DH-10B *E. coli* cells and plasmids were isolated using Takara Bio USA, Inc. NucleoSpin® Plasmid purification kit. Plasmids were sequenced by Plasmidsaurus to confirm correct mutation. IndF mutants were expressed and purified the same as described for the wild-type IndF.

In vitro Enzymatic Assays

In vitro enzymatic assays of heterologously expressed MBP-IndF was performed with 10 µM IndF and 100µM IndA with 1 mM ATP, 4 mM $MgCl_2$ in buffer consisting of 20 mM HEPES, 300 mM NaCl, pH 7.5. This assay was run alongside separate controls containing no ATP, no $MgCl_2$, and no IndA. The protease assays were carried out using the protease in the corresponding gene cluster from *S. equi* (SeIndP) using the same buffer and conditions as listed above, with the addition of 1 µM protease (SeIndP). Reactions without the protease were incubated overnight at room temperature, then the protease was added on the following day. After ~16 hours, the reactions were quenched using equal volume of 0.1% formic acid in 75% acetonitrile and was centrifuged for 10 minutes at 16000 × g. The assays were then subjected to UHPLC-HRMS analysis.

Size Exclusion Analysis: Gel filtration standard curve

The Bio-Rad Gel Filtration Standard mixture was used to generate the protein standard curve (Figure S21). 0.5 mL of mixture containing thyroglobulin (670,00 Da), γ-globulin (158,000 Da), ovalbumin (44,000 Da), myoglobin (17,000 Da), and vitamin B12 (1,350 Da), was inject to a Cytiva HiLoad 16/60 Superdex 200 size exclusion column pre-equilibrated with GF Buffer (20 mM HEPES, 300 mM NaCl, 10% glycerol, pH 8.0). The elution times were plotted as follows: log (molecular weight) versus the gel phase distribution coefficient (Kav). (Kav = (Ve-Vo)/(Vt-Vo); Ve: analyte retention volume; Vo: void volume; Vt: total volume). MBP-IndF, MBP-IndF:IndA, MBP-IndF+ATP+MgCl2 and TEV cleaved IndF, were analyzed one at a time on a separate run using the same conditions and its elution time was used to estimate its molecular weight.

UHPLC-HRMS Analysis

Analysis of IndF enzymatic assays was performed on Thermo Fisher Orbitrap Elite ETD instrument linked to a Dionex Ultimate 3000 UHPLC system with Kinetex® 1.7 µm C18 (50 x 2.1 mm) with a flow rate of 0.3 mL min^{-1}. Analysis of the reaction was completed using 35 V collision energy in positive ionization mode. Mobile phases for UHPLC were 0.1% (v/v) formic acid in water (A) and 0.1% (v/v) formic acid in ACN (B). The linear gradient from 20%-70% B, with the column at 35°C for a 10-minute run. For MS/MS fragmentation, Thermo Fisher Scientific Q Exactive Plus linked to Acquity UPLC system. For the fragmentation, a HCD collision energy of 20 eV was used with the system in positive mode. The UHPLC gradient used was 20-70% B, with the same mobile phases as previously mentioned, with the column at 35°C for a 13-minute run on Acquity UPLC BEH 1.7 µm C18 (50 X 2.1 mm) column with a flow rate of 0.3 mL min^{-1}. For all samples, internal standard of 1 µm fluorescein was added before analysis run to normalize retention times. For

MS/MS fragmentation, the two hundred most abundant ions were selected to generate the *b* and *y* ions for fragmentation tables. All the activity assay traces were prepared using a mass tolerance of 20 ppm, mass precision of four decimals, and enabling curve smoothing (Gaussian, 15 points).

Solid-phase Peptide Synthesis of IndA

IndA solid-phase peptide synthesis was performed on a Biotage Initiator+ Alstra Automated Microwave Peptide Synthesizer using L-isoleucine-2-chlorotrityl resin. Each peptide started synthesis at the C-terminus and was made toward the N-terminus. N-α-Fmoc-L-amino acids were purchased from ChemImpex and coupling agents diisopropylcarbodiimide (DIC) and Oxyma at 0.5 M solution in dimethylformamide (DMF) using microwave irradiation for each amino acid addition. The process began with swelling the resin with DMF at 70°C for 20 minutes, deprotected with 20% (v/v) piperidine in DMF at 25°C for 10 minutes, coupled using DIC/Oxyma at 75 °C for 5 minutes, then deprotected with 20% (v/v) piperidine in DMF at 25°C for 10 minutes. Following this initial set up, each amino acid was sequentially added with the methods for coupling with DIC/Oxyma at 75°C for 5 minutes and deprotection with 20% (v/v) piperidine in DMF at 25 °C). The bulkier amino acids (tryptophan, phenylalanine, and tyrosine) were double coupled at 50 °C for 10 minutes each round, Asn(-12), Arg(-2), and Asn5 were coupled at 50 °C for 10 minutes, and Ile6 and Leu7 were double coupled using the normal conditions described above and were deprotected at 50 °C. At the end of the synthesis, the resin was washed three times with dichloromethane (DCM), three times with ethyl ether, and then was dried. The dried resin with peptide product was cleaved using a trifluoroacetic acid (TFA) cocktail made of 95% (v/v) TFA, 2.5% (v/v) H_2O, and 2.5% (v/v) triisopropylsilane (TIPS). The peptide cleavage was performed in the peptide synthesis reaction vessel stirring at 700 RPM for 3 hours at room temperature. The reacted solution was pushed through the reaction vessel into a clean vial and dried using line air until volume was decreased to ~1 mL. Pre-chilled ethyl ether was added to precipitate peptide and sample was centrifuged, ethyl ether supernatant was decanted, and peptide was dried using line air. Dried solid was dissolved in 50% (v/v) acetonitrile-basic water and mass of crude peptide was confirmed using UHLC-HRMS. Peptides were purified using HyperSep™ C18 columns using aqueous 10 mM ammonium bicarbonate and acetonitrile. Synthesized peptides eluted between 20-30% acetonitrile. Fractions containing peptide were dried using the SpeedVac (Savant SpeedVac SPD120 Vacuum Concentrator) connected to vapor trap (Savant RVT5105 Refrigerated Vapor Trap) and vacuum pump (Thermo Scientific VLP120 Vacuum Pump) overnight, re-dissolved in acetonitrile, and transferred to clean, pre-weighed scintillation vial and dried in the speedvac, weighed then stored at -20°C.

Purification of linear IndA$_{N5-I9}$ and IndA-Cyclic$_{N5-I9}$

The dried material was dissolved in 5-6 mL of water (1% NaOH) and fractionated via flash chromatography on a CombiFlash EZ Prep (Teledyne ISCO) with the following parameters: injection concentration 100 mg/5 mL; RediSep Rf High Performance 5.5 – 110 mg HP C18 reverse phase column; LC gradient solvent A: water (0.1 % formic acid); solvent B: acetonitrile (0.1% formic acid); eluting gradient (%B, v/v) ; 10-50 % over 25 mins; flow rate 18 mL/min; 5 mL fractions were collected; wavelength window 200-300 nm

NMR Analysis of IndA$_{N5-I9}$ and IndA-Cyclic$_{N5-I9}$

Both the synthesized IndA$_{N5-I9}$ and corresponding IndA-Cyclic$_{N5-I9}$ were analyzed by one-dimensional (1D) and 2-dimensional (2D) NMR in DMSO-d_6 in 5 mm NMR tubes (Wilmad LabGlass). All the NMR experiments were recorded on a JEOL ECA-500 MHz NMR spectrometer (JEOL Ltd.). The chemical shift (δ) values are given in parts per million (ppm), and the coupling constants (J values) are in Hz. All the Structural assignments were made by analyzing ^{1}H NMR with additional information from COSY, HSQC, HMBC and ROESY experiments.

NMR data for IndA$_{N5-I9}$:

^{1}H NMR (500 MHz, DMSO-d_6): δ 10.75 (d, J = 2.4 Hz, 1H), 9.14 (bs, 1H), 8.19 (d, J = 7.6 Hz, 1H), 8.12 – 8.01 (m, 2H), 7.92 (d, J = 8.3 Hz, 1H), 7.52 (d, J = 7.9 Hz, 1H), 7.44 (d, J = 2.6 Hz, 1H), 7.30 (d, J = 8.1 Hz, 1H), 7.28 – 7.20 (m, 4H), 7.20 – 7.12 (m, 1H), 7.06 (d, J = 2.4 Hz, 2H), 7.05 – 7.01 (m, 1H), 6.96 – 6.89 (m, 4H), 6.59 (d, J = 8.4, 2H), 4.61 (td, J = 8.7, 4.6 Hz, 1H), 4.48 – 4.41 (m, 1H), 4.36 (td, J = 8.6, 4.5 Hz, 1H), 4.15 (dd, J = 8.3, 5.7 Hz, 1H), 3.48 (dd, J = 8.7, 4.4 Hz, 2H), 3.10 – 3.0 (m, 2H), 2.93 – 2.75 (m, 3H), 2.67 (dd, J = 14.0, 9.2 Hz, 1H), 2.43 (d, J = 4.3 Hz, 1H), 2.13 (dd, J = 15.2, 8.7 Hz, 1H), 1.87 – 1.72 (m, 1H), 1.49 – 1.38 (m, 1H), 1.25 – 1.20 (m, 1H), 0.90 – 0.76 (overlap, 6H).

^{13}C NMR (125 MHz, DMSO-d_6): 173.3, 172.8, 172.6, 171.1, 170.9, 170.7, 155.7, 137.7, 136.0, 130.1, 129.2, 128.0, 127.8, 127.4, 126.2, 123.6, 120.8, 118.4, 118.1, 114.8, 111.2, 109.8, 56.7, 54.5, 53.7, 53.3, 51.6, 37.4, 36.6, 36.5, 28.4, 27.7, 24.7, 15.6, 11.4.

NMR data for IndA-Cylic$_{N5-I9}$:

^{1}H NMR (500 MHz, DMSO-d_6): δ 8.93 (s, 1H), 8.36 (d, J = 7.7 Hz, 2H), 8.21 (s, 1H), 7.80 (d, J = 7.7 Hz, 1H), 7.56 (s, 1H), 7.40 (s, 1H), 7.37 – 7.25 (m, 3H), 7.23 – 7.17 (m, 2H), 6.90 (s, 1H), 6.75 (d, J = 7.9 Hz, 1H), 6.54 (d, J = 8.0 Hz, 1H), 4.64 (s, 1H), 4.37 (q, J = 7.9 Hz, 1H), 4.23 – 4.19 (m, 2H), 3.51 – 3.49 (m, 2H), 3.08 – 3.01 (m, 2H), 2.95 (dd, J = 13.4, 7.5 Hz, 1H), 2.80 (overlap, 2H), 2.66 – 2.54 (m, 2H), 2.46 (d, J = 3.9 Hz, 1H), 2.30 (dd, J = 15.2, 8.8 Hz, 1H), 2.25 – 2.15 (s, 1H), 1.45 – 1.50 (m, 2H), 0.80 (m, J = 5.3 Hz, 6H).

Marfey's Analysis

To determine the stereochemistry of $IndA_{N5\text{-}I9}$ and IndA-$Cyclic_{N5\text{-}I9}$, Marfey's analysis was used. Standards were prepared to match the core amino acids of the linear IndA and its predicted modified cyclic product. To make each amino acid standard, 0.2 mg of the L- and D-amino acid was aliquoted into separate reaction vials. To each vial, 50 µL of water, 20 µL of 1 M $NaHCO_3$, and 100 µL 1% w/v Marfey's reagent (sodium (2,4-dinitro-5-fluorophenyl)-L-alaninamide) in acetone were added. The reaction mixture was incubated at 40 °C for 1 h with periodic agitation. The reactions were quenched by adding 10 µL of 2 N HCl and dried under a nitrogen stream. Dried samples were dissolved in 1.7 mL of MeOH and injected both individually and as a combined mixture (5 µL) onto UHPLC-HRMS (Thermo Orbitrap Elite mass spectrometer coupled to a Dionex Ultimate 3000 UHPLC), with a gradient of 15–50% B over 8 min on a BEH C18 1.7 µm 100 Å 50 × 2.1 mm LC column using solvents of A: 0.1% formic acid in water and B: 0.1% formic acid in acetonitrile. Derivatized Products were observed at 340 nm and monitored by their expected $[M + H]^+$ ion.

Hydrolyzed derivatized IndA-$Cyclic_{N5\text{-}I9}$ was generated by adding 0.5 mL of 6 N HCl to a 0.2 mg sample in a 4 mL reaction vial, incubating for either 24 h or 8 h (to assure the integrity of the tryptophan residue during hydrolysis) at 90 °C, and then evaporation under a stream of nitrogen. To the hydrolysis product was added 25 µL of water, 10 µL of 1 M $NaHCO_3$ and 50 µL of 1% w/v Marfey's reagent in acetone. This was incubated for 1 h at 40 °C with periodic agitation. The reaction was quenched by adding 5 µL of 2 N HCl, dried under a stream of nitrogen and resuspended in 200 µL of MeOH. The sample was injected (6 µL) onto UHPLC-HRMS using the same conditions and the same instrument as amino acids standards. Tryptophan standards were digested and derivatized concurrently with IndA to show that the tryptophan is degraded during 24 h digestion, but not in the 8 h digestion. All the amino acids were found to have the L-configuration as expected. In addition to hydrolyzing the peptide bonds, the acid hydrolysis conditions appeared to cleave the Asn-Trp bond to generate L-Trp but no mass feature consistent with L-Asn was detected. Instead, mass features were observed corresponding to Asp (hydrolysis product of Asn). Similar results were observed with the standard Asn.

SUPPLEMENTARY FIGURES AND TABLES

Table S1. Accession codes and protein sequences

NCBI Accession Code	Name	Codon Optimized Sequence	Amino Acid Sequence
WP_000371017.1	IndF	ATGGATAATTATTTCCCGAAATGGAACCAGGATCGTATTGTGTATCAG<br>TGGAAAAACGATCGTCTGCGCATTGGCGCGGATGATGTGGATGTGT<br>TAGAAATTACCGGCTACAGCGATTTTTGGAGCGATCTGATTTCGTGC<br>TGCAACGGCATTAATTCATTCGAAGAAATCAAAGATCTGCTGCGTAAA<br>AAATATGATATTAGCGAAAACATTATTGAAAAATACATTAGCAAATTCA<br>GCGATCGCAACCTGCTGGAGATTCTGGATCGCCCGGTGAACCAGAT<br>CGATCATTATCTGATTAACGAAAGCCTGGAAACCTATTACAGTAGCG<br>AAGGCATTGGCGGCATCAAACTGCTGGAAAAACTGAGCAACCTGAAA<br>GTAACCATCCTGGGCTGCGGCGCGGGCGGCAGCCACATTGCGTTG<br>CAGCTGGCCCAGCTGGGCGTGGGCCGCCTGCACCTGGTGGATGAC<br>GATATTGTGAAAGAAAACAACATTAATCGCCAGAGCATGTTCACCTTT<br>AACGATATTGGCAAATATAAAGTGGATTGTGTTAAAGATTGCATTCTG<br>AAACGTAACTATCAGTGCGTGGTGACCAAACGCAAACTGAAAATGAG<br>CACCGTGGATGCCGTGAAAAAAGAAATTAGCGAAAGCGACTGGGTG<br>TTTTGTTGCATGGATGAACCGCCGTATATTGCCCAGCGCCTGGTGAA<br>TCGCGCATGCTACCTGTTTAACATTCCGAGCATTTACTGCTTTAGCCA<br>GCGTAGCGCGGGCAAACTGCTGTTTTGCAATCCGAATATTCAGAACA<br>TTGGCTGCGTGGATTGCCTGCTGTATGAACAGGATAGCGATAATTTC<br>CAGAACCTGGTGAAAAAATTCAGCAACTATGATGGCAAACTGATTAC<br>CGCGAACATCCTGACTAACATCCTGCTGCTGAGCAGCTGGGTTGTG<br>AAAAAATGGCTGGATTGTGTTACCGAAAAAAACAGCAACGTGTGGAA<br>TACCCTGTTTCGTTTTGATTTTTATAGCTTTCGCGAAGATGAATTCAAA<br>CATTTTAGCAAACAGAGCCATTGCCCGACCTGCGGCCATGATTTTGA<br>TAAAAGCAAACTGTGGGAAATTCTTAAAATTGATGAATAA	MDNYFPKWNQDRIVYQWKN<br>DRLRIGADDVDVLEITGYSDF<br>WSDLISCCNGINSFEEIKDLLR<br>KKYDISENIIEKYISKFSDRNLL<br>EILDRPVNQIDHYLINESLETY<br>YSSEGIGGIKLLEKLSNLKVTI<br>LGCGAGGSHIALQLAQLGVG<br>RLHLVDDDIVKENNINRQSMF<br>TFNDIGKYKVDCVKDCILKRN<br>YQCVVTKRKLKMSTVDAVKK<br>EISESDWVFCCMDEPPYIAQ<br>RLVNRACYLFNIPSIYCFSQR<br>SAGKLLFCNPNIQNIGCVDCL<br>LYEQDSDNFQNLVKKFSNYD<br>GKLITANILTNILLLSSWVVKK<br>WLDCVTEKNSNVWNTLFRFD<br>FYSFREDEFKHFSKQSHCPT<br>CGHDFDKSKLWEILKIDE
WP_001034978.1	SpIndP	ATGAACAAAAAAATTATTTTTAAGAAAAACCAGGCGAGCAAATTTGC<br>GTATTTTAGCCTGATGTTTGTGGCGGGTACCAGCATTGAACAGGTG<br>AATGAACTGGGCTTCTCGCACCTGATTGAACACCTGCTGATCCGCG<br>CCGGCAACGAGCAGAGCCTGAACGAACTGTTCGATATGAACGGCG<br>CGGCGATTAAAGGCGAAACCAGCCGCGATTACATTAACCTGAGCG<br>GCTATTGCCTGGCGGAAGATTTTAATAAAATTTTCAAAATCCTGATTA<br>GCCGTATTTTTAATCTGTCGATTACCGAAGATGAACTGCTGCGTGAA<br>AAAAAAATTGTGCTGATTGAACTGAACCAGTATGAGAATAGCAAAAA<br>AAGCATTAACGATAACCGCGTGATCTTTAAAAACAGCAGCTGGAGC<br>ATCGATATTATTGGCACCCGTGGCAACATTGAATATGTGAGCCTGG<br>AAACCATTTACAAATTTTACATTAAAAACAGCATTAAAATTTAA	MNKKIIFKKNQASKFAYFSLM<br>FVAGTSIEQVNELGFSHLIEHL<br>LIRAGNEQSLNELFDMNGAAI<br>KGETSRDYINLSGYCLAEDFN<br>KIFKILISRIFNLSITEDELLREK<br>KIVLIELNQYENSKKSINDNRV<br>IFKNSSWSIDIIGTRGNIEYVSL<br>ETIYKFYIKNSIKI
WP_012678787.1	SeIndP	ATGAACAAAAAAATTATTTTTAAAGGCGATCAGGATAGCAAATTTGC<br>ATATTTTAGCCTGATGTTCAGCGCCGGTACCGCGATCGAAAACACC<br>GAAGAACTGGGTTTTAGCCACCTGATTGAGCACCTGCTGCTGCGCA<br>GCGGCGGCGAACAGAGCCTGAATGAACTGTTTGATAACAACGGCG<br>CGTTCATTGGTGGCGAAACCAGCCGCGATTATATTAACCTGATGGG<br>TTATTGCAAAGCCGAAAATTTTAAAAACATTTTTGAAGCCATTGTTAG<br>CCGTGTGTTCAACCTGAATCTGACCGAAGAAGAACTGCTGCGCGAA<br>AAACGCGTGGTTCTGGTGGAACTGACCCAGTATGAAAATGGCAGCA<br>AAACCGAAAAACTGGTGAGCGATAACCGCCTGATTTTCAAAAACTC<br>GAAATGGAGCGAAGACATTATTGGTGTGCGTGAAAACATCGAAAGC<br>GTGGATCTGAAAAAACTGTACAAATTTTATACCGAAAACATTCAGAA<br>CGGCGAATTTCAGATTGCGATCAGCGGCCCGAATCATCTGAAAGAA<br>GAAATTGCAATTATCGAAAACAAACTGCCGGTGGGCCGTACCCCGG<br>TGAAAAGCAACTTTCCGATTTTTAGTAGCGGCGTGACCGAACGCAA<br>AAAAAACCAGCAGGTGAGCGAAATTTCAATGTACATTGATATTAGCA<br>AGATGACCACCAGCAGCCACGATGTGGCGATTCTGACCATTCTGAA<br>CGCGATGCTGACCGGTGTGAAAGGCAGCGTTCTGGGCGGCAAACT<br>GCGCACCAAAAACCAGTGGGTGTATAACATTATTAGCTTTCCGATTT<br>TTTATAATGGCCTGACCATTCTGAAAATTCTGACCCGTACGCCGGAA<br>ATTCATAAACATCAGGTGGTGCAGGTGTTAAAAGAAGATCTGGTGA<br>ACCGTGAAGATCTGAAAAATACCAAACTGTTTGATAAAGCGAAAAAA<br>CGCGTGATTAATGAAGTGCTGATGTCATACGAAGTGAAAAAAGTGG<br>AATTCCTGAAAACCCTGTGTCGCGAAAAACTGTTCAATATTCCGAGC<br>TGGGAAAGCGTTACCGGCGAAATTGAAAAAGTGAGCCTGAATGAAC<br>TGAATCAGTTTGCGAAAGATGCCCTGATGCAGAACAAACAGTTTCA<br>CATTATTATTAATTGCTAA	MNKKIIFKGDQDSKFAYFSLM<br>FSAGTAIENTEELGFSHLIEHL<br>LLRSGGEQSLNELFDNNGAFI<br>GGETSRDYINLMGYCKAENF<br>KNIFEAIVSRVFNLNLTEEELL<br>REKRVVLVELTQYENGSKTE<br>KLVSDNRLIFKNSKWSEDIIGV<br>RENIESVDLKKLYKFYTENIQ<br>NGEFQIAISGPNHLKEEIAIIEN<br>KLPVGRTPVKSNFPIFSSGVT<br>ERKKNQQVSEISMYIDISKMT<br>TSSHDVAILTILNAMLTGVKG<br>SVLGGKLRTKNQWVYNIISFP<br>IFYNGLTILKILTRTPEIHKHQV<br>VQVLKEDLVNREDLKNTKLFD<br>KAKKRVINEVLMSYEVKKVEF<br>LKTLCREKLFNIPSWESVTGE<br>IEKVSLNELNQFAKDALMQNK<br>QFHIIINC

ADM91236.1	IndA	ATGATCATGATCATGGAAGACTTCAACATCCTGGAACTGGAATTCGA AGAAATCCGTGAA GAAGACACCACCAACTGGTACTTCATC	MIMIMEDFNILELEFEEIREED TTNWYFI
ASB95706.1	SeIndA	ATGAACATGATCATGGAAGACTTCGAAGTTCTGGAACTGGAATTCGT TGAAGTTCGTAAC GAACAGACCACCAACTGGTACTTCGTT	MNMIMEDFEVLELEFVEVRN EQTTNWYFV

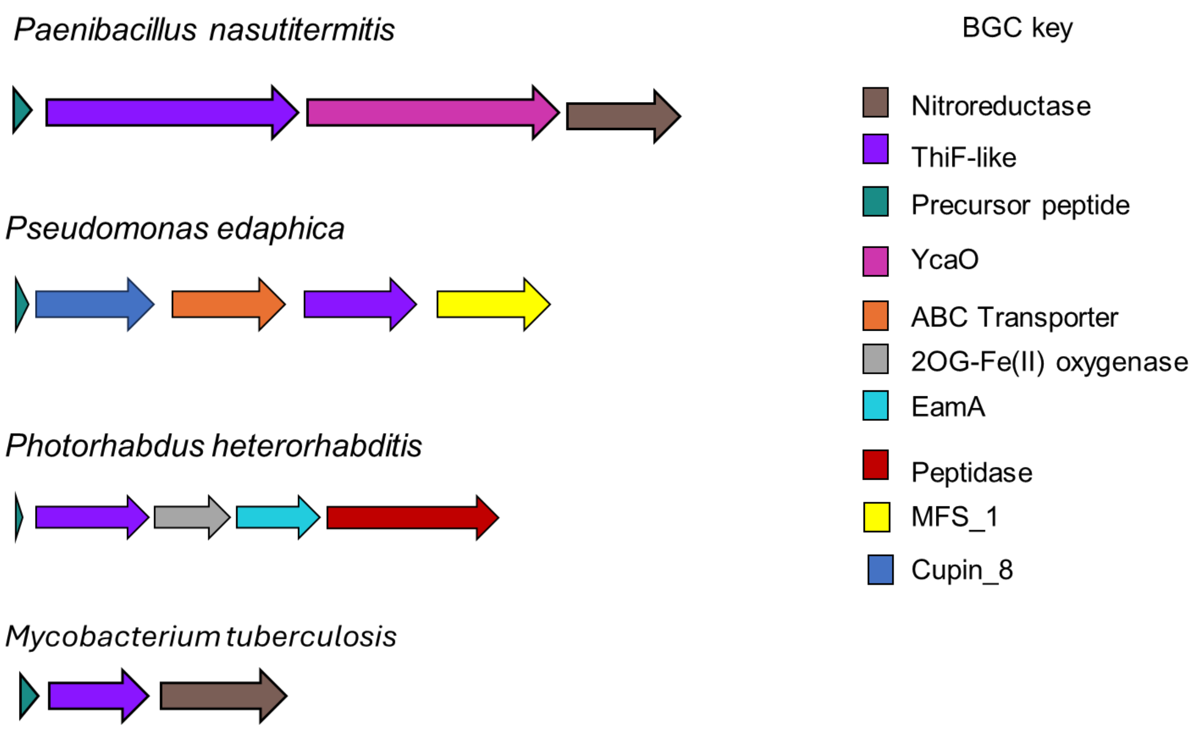

Figure S1. Examples of other biosynthetic gene clusters containing ThiF-like enzymes from sequence similarity network.

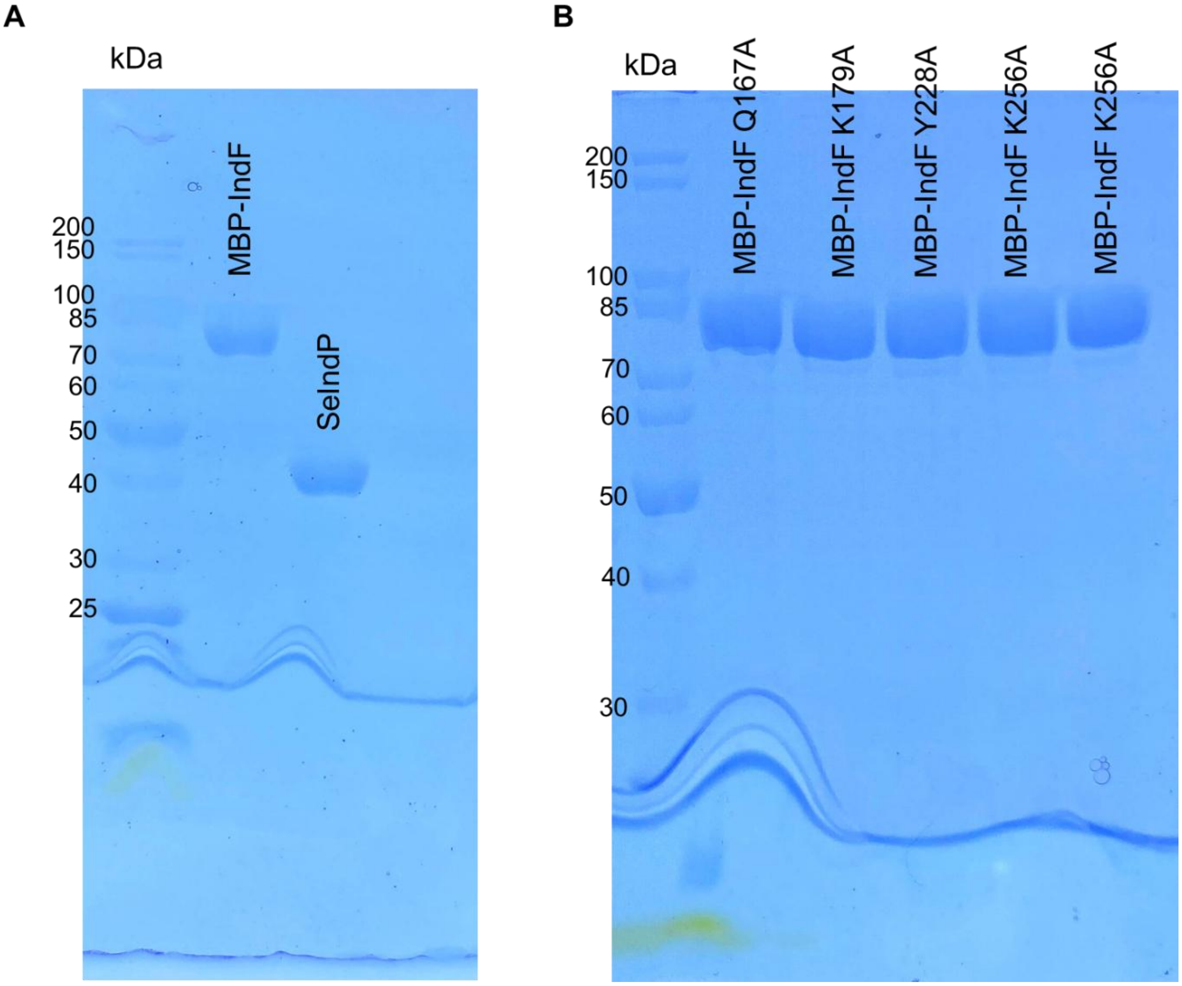

Figure S2. Heterologous expression of *ind* proteins: MBP-IndF (89.01 kDa), SeIndP (44.5 kDa) (A), and MBP-IndF mutants (88.98 kDa) (B). SDS-PAGE gel analysis of expressed proteins with Broad Range Protein Standard from NE Biolabs.

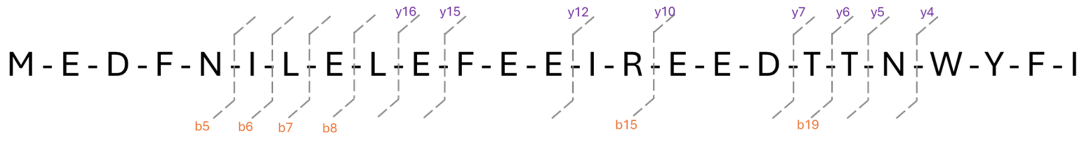

Fragment	calc m/z	Obs m/z	Δm (ppm)
b5	637.2286	637.2271	2.42
b6	750.3127	750.3102	3.33
b7	863.3968	863.3941	3.08
b8	992.4393	992.4359	3.48
b15	1908.9048	1908.8963	4.43
b19	2383.0646	2383.0588	2.42
y4	628.3130	628.3113	2.69
y5	742.3559	742.3524	4.74
y7	944.4513	944.4467	4.85
y10	1317.5634	1317.5614	1.52
y12	1586.7486	1586.7430	3.51
y15	1991.9022	1991.8998	1.18
y16	2120.9448	2120.9390	2.71

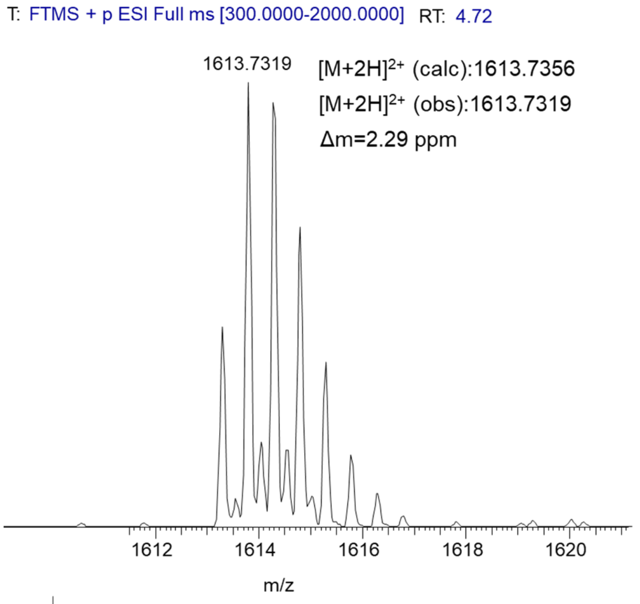

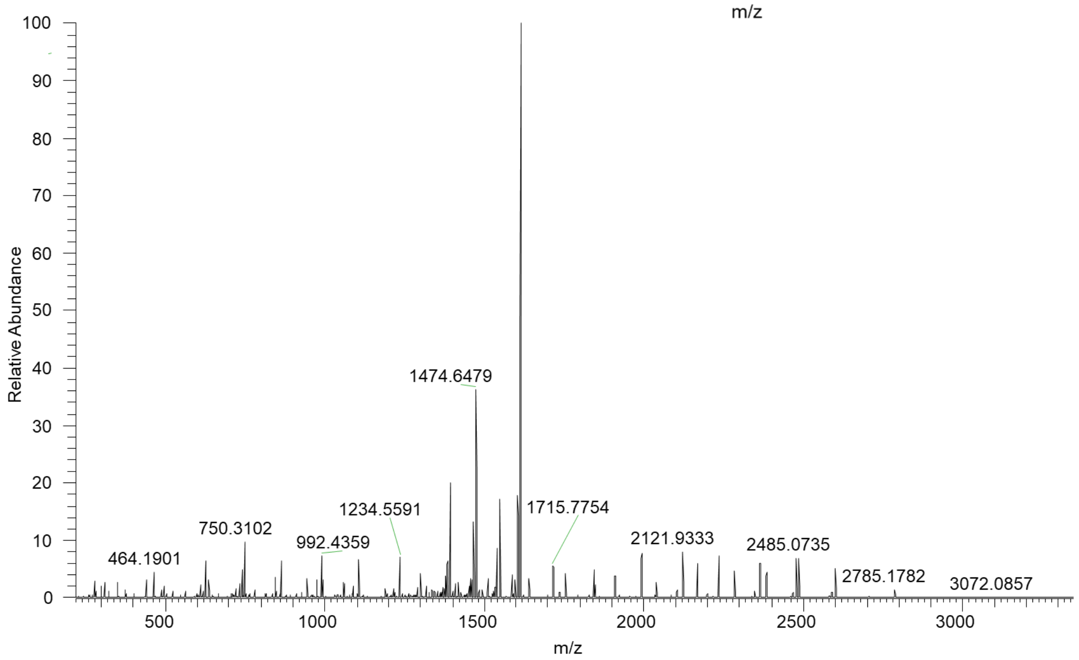

Figure S3. MS/MS spectrum for IndA.

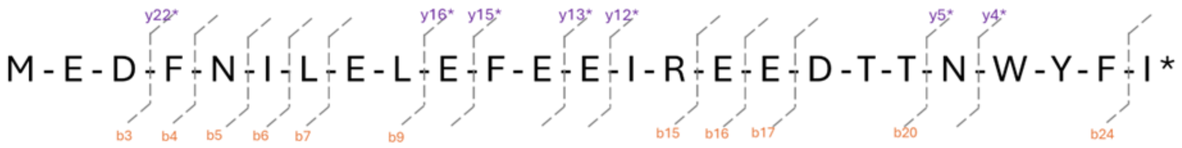

Fragment	calc m/z	Obs m/z	Δm (ppm)
b3	376.1173	376.1160	3.46
b4	523.1857	523.1847	1.93
b5	637.2286	637.2262	3.83
b6	750.3127	750.3093	4.53
b7	863.3968	863.3929	4.47
b9	1105.5234	1105.5181	4.8
b15	1908.9048	1908.9036	0.61
b16	2037.9474	2037.9375	4.83
b17	2166.9899	2166.9816	3.85
$b20^{2+}$	1242.5598	1242.5572	2.06
$b24^{2+}$	1547.6868	1547.6903	2.42
y4*	610.3024	610.3004	3.26
y5*	724.3453	724.3419	4.72
y12*	1568.7380	1568.7349	1.96
y13*	1697.7806	1697.773	4.45
y15*	1973.8916	1973.8873	2.16
y16*	2102.9342	2102.9348	0.31
$y22^{2+}$ *	1416.6738	1416.6679	4.14

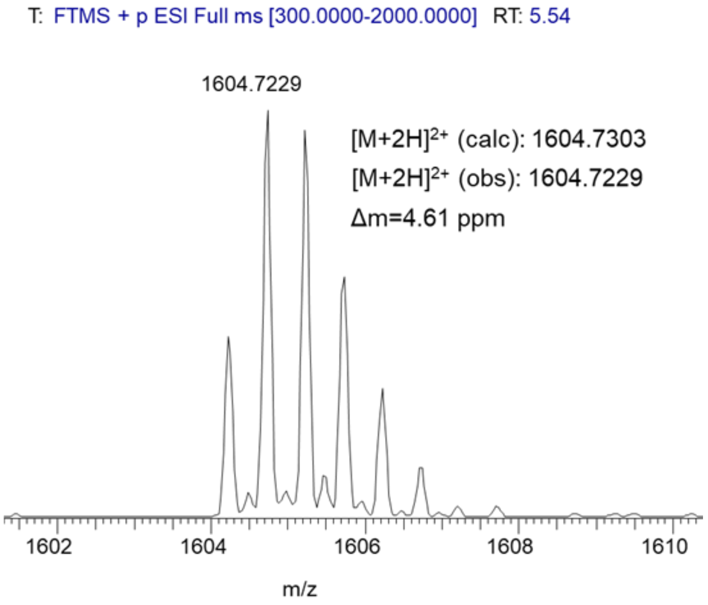

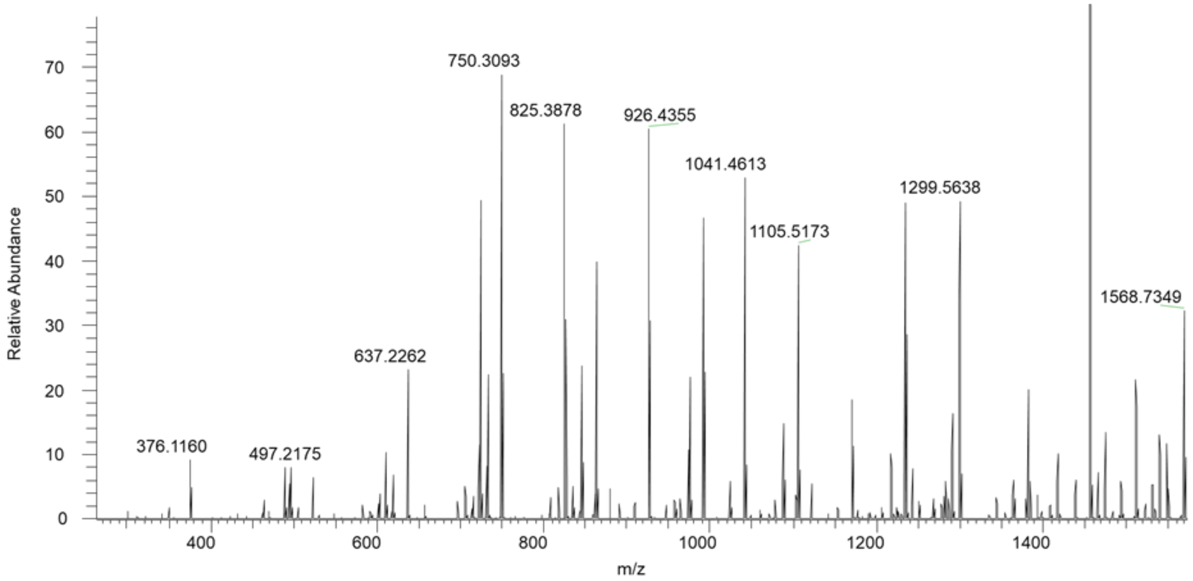

Figure S4. MS/MS spectrum for IndA-Cyclic after incubation with IndF. Star (*) indicates the loss of one water in the MS/MS fragment ions.

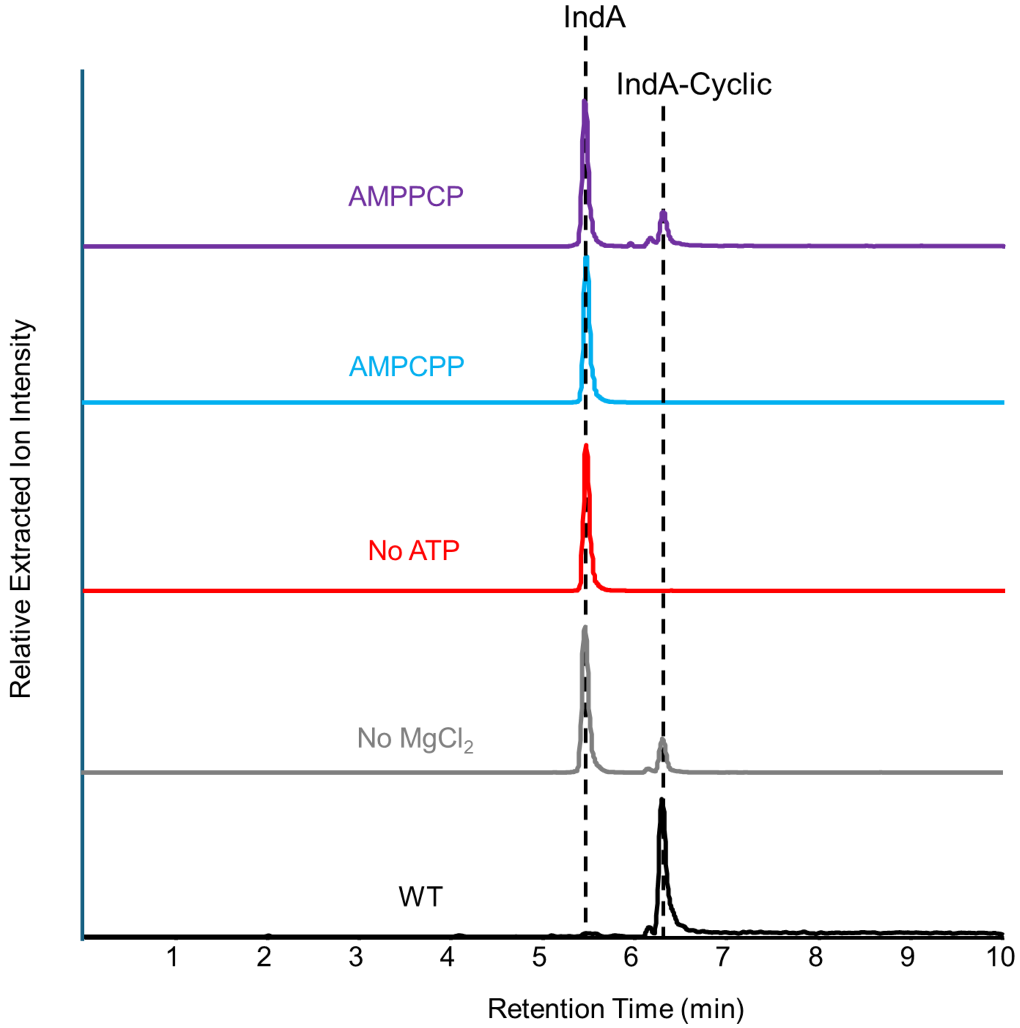

Figure S5. UHPLC-HRMS analysis of IndF assays with varied cofactors/co-substrates. Extracted ion chromatograms for full assay (black), assay without $MgCl_2$ (gray), assay without ATP (red), assay with adenosine 5'-[α,β-methylene] triphosphate (AMPCPP) in place of ATP (blue), and assay with adenosine 5'-[β,γ-methylene] triphosphate (AMPPCP) in place of ATP (purple). (IndA: [1613.7319 *m/z* $[M+2H]^{2+}$, IndA-Cyclic: 1604.7229 *m/z* $[M+2H]^{2+}$) using a mass tolerance of 20 ppm.

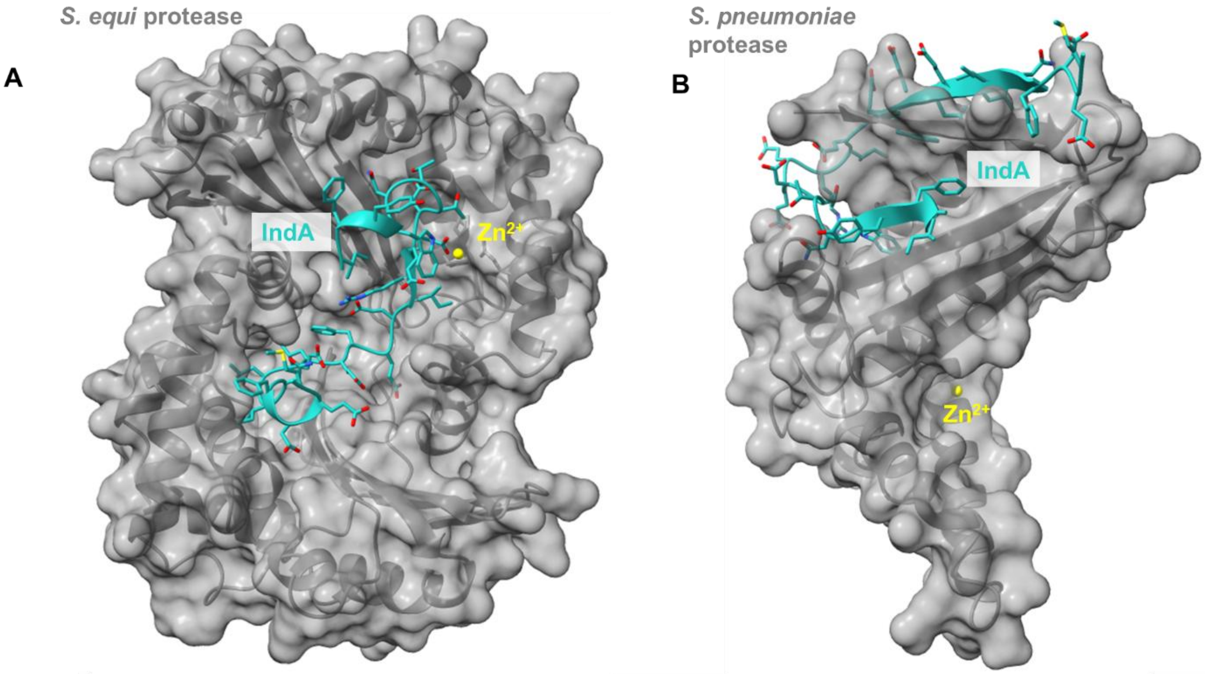

C

```
ref|WP_001034978.1|/1-168     1 MNKKIIFKKNQASKFAYFSLMFVAGTSIEQVNELGFSHLIEHLLIRAGNEQSLNEL  56
ref|WP_012678787.1|/1-391     1 MNKKIIFKGDQDSKFAYFSLMFSAGTAIENTEELGFSHLIEHLLLRSGGEQSLNEL  56

ref|WP_001034978.1|/1-168    57 FDMNGAAIKGETSRDYINLSGYCLAEDFNKIFKILISRIFNLSITEDELLREKKIV 112
ref|WP_012678787.1|/1-391    57 FDNNGAFIGGETSRDYINLMGYCKAENFKNIFEAIVSRVFNLNLTEEELLREKRVV 112

ref|WP_001034978.1|/1-168   113 LIELNQYENSKKS---INDNRVIFKNSSWSIDIIGTRGNIEYVSLETIYKFYIKNS 165
ref|WP_012678787.1|/1-391   113 LVELTQYENGSKTEKLVSDNRLIFKNSKWSEDIIGVRENIESVDLKKLYKFYTENI 168

ref|WP_001034978.1|/1-168   166 IKI----------------------------------------------------- 168
ref|WP_012678787.1|/1-391   169 QNGEFQIAISGPNHLKEEIAIIENKLPVGRTPVKSNFPIFSSGVTERKKNQQVSEI 224

ref|WP_001034978.1|/1-168       --------------------------------------------------------
ref|WP_012678787.1|/1-391   225 SMYIDISKMTTSSHDVAILTILNAMLTGVKGSVLGGKLRTKNQWVYNIISFPIFYN 280

ref|WP_001034978.1|/1-168       --------------------------------------------------------
ref|WP_012678787.1|/1-391   281 GLTILKILTRTPEIHKHQVVQVLKEDLVNREDLKNTKLFDKAKKRVINEVLMSYEV 336

ref|WP_001034978.1|/1-168       -------------------------------------------------------
ref|WP_012678787.1|/1-391   337 KKVEFLKTLCREKLFNIPSWESVTGEIEKVSLNELNQFAKDALMQNKQFHIIINC  391
```

D *S. equi* and *S. pnuemoniae* Precursor Peptide Alignment

```
ASB95706.1/1-29   1 MNMIMEDFEVLELEFVEVRNEQTTNWYFV
ADM91236.1/1-29   1 MIMIMEDFNILELEFEEIREEDTTNWYFI
```

Figure S6. *S. equi* and *S.pneumoniae* protease alignment: AlphaFold 3 models of (A) *S. equi* protease (SeIndP) and (B) *S. pneumoniae* protease (SpIndP) (C) Sequence alignment of SeIndP and SpIndP, (D) Sequence alignment of precursor peptides from *S. equi* (ASB95706.1) and *S. pneumoniae* (ADM91236.1) clusters.

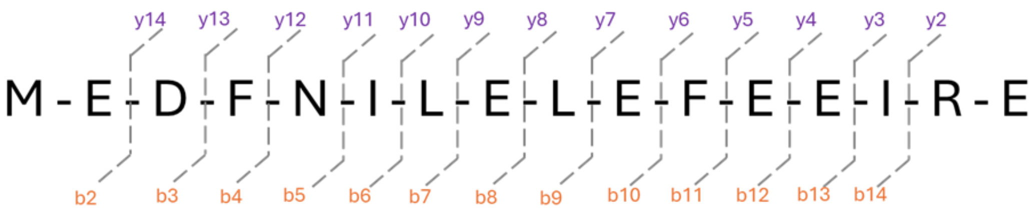

Fragment	calc m/z	Obs m/z	Δm (ppm)
b_2	261.0904	261.0898	2.14
b_3	376.1173	376.1165	2.13
b_4	523.1857	523.1851	1.17
b_5	637.2286	637.2286	0.06
b_6	750.3127	750.3121	0.80
b_7	863.3968	863.3969	0.16
b_8	992.4393	992.4374	1.96
b_9	1105.5234	1105.5229	0.46
b_{10}	1234.5660	1234.5621	3.16
b_{11}	1381.6344	1381.6326	1.31
b_{13}	1639.7196	1639.7152	2.68
b_{14}	1752.8037	1752.7964	4.14
y_2	304.1616	304.1608	2.56
y_3	417.2456	417.2450	1.53
y_4	546.2882	546.2880	0.42
y_5	675.3308	675.3316	1.15
y_6	822.3992	822.3973	2.35
y_7	951.4418	951.4386	3.38
y_8	1064.5259	1064.5246	1.20
y_9	1193.5685	1193.5651	2.82
y_{10}	1306.6525	1306.6484	3.16
y_{11}	1419.7366	1419.7326	2.81
y_{12}	1533.7795	1533.7740	3.60
y_{13}	1680.8479	1680.8422	3.41
y_{14}	1795.8749	1795.8713	1.99

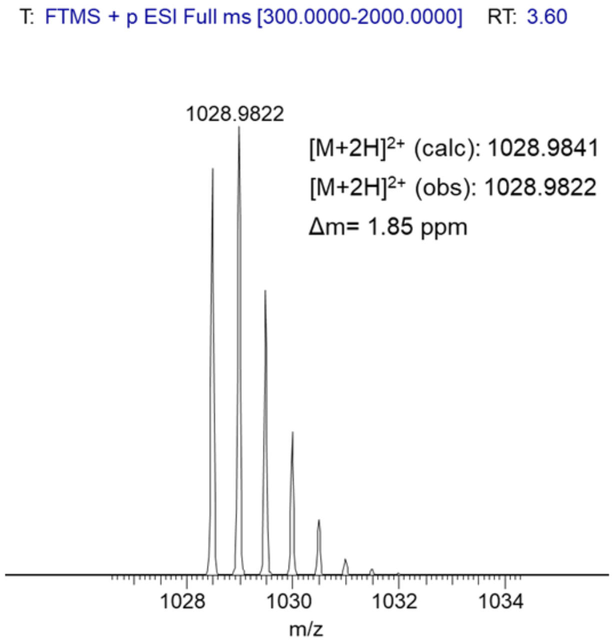

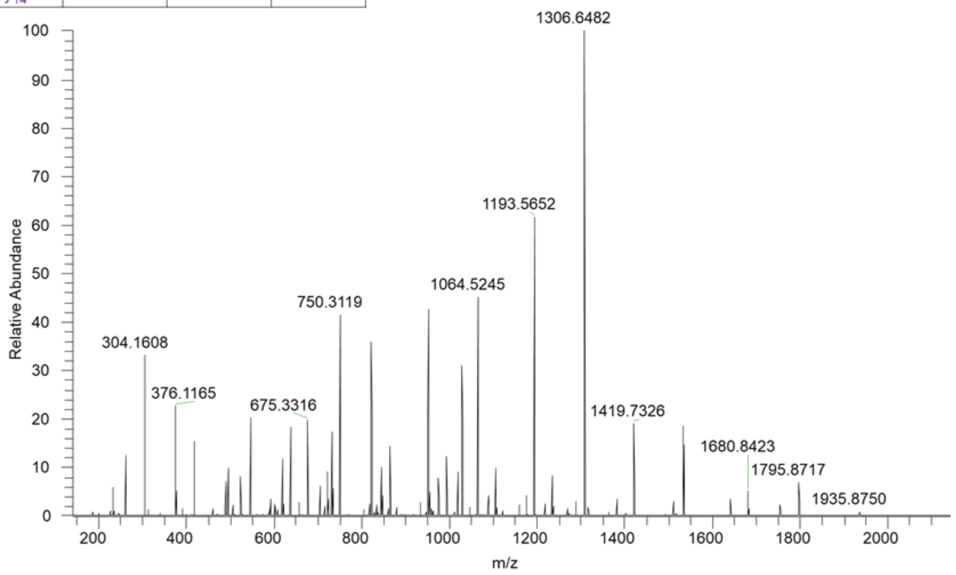

Figure S7. MS/MS spectrum for cleaved IndA leader after incubation with SeIndP.

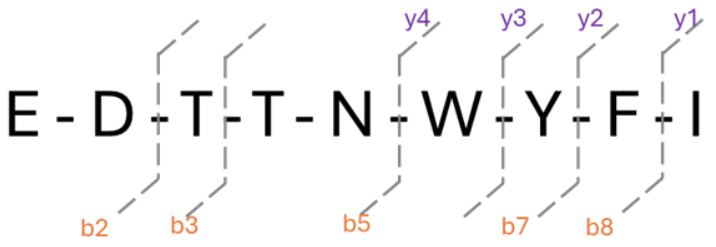

Fragment	calc m/z	Obs m/z	Δm (ppm)
b_2	245.0768	245.0758	4.12
b_3	346.1245	346.1231	4.02
b_5	561.2151	561.2131	3.56
b_7	910.3577	910.3535	4.66
y_1	132.1019	132.1015	3.33
y_2	279.1703	279.1690	4.84
y_3	442.2337	442.2327	2.22
y_4	628.3130	628.3109	3.33

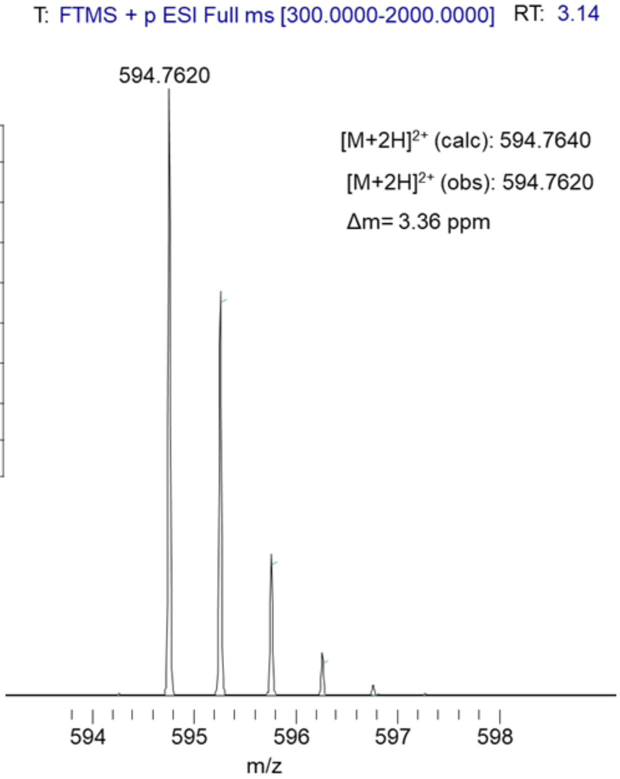

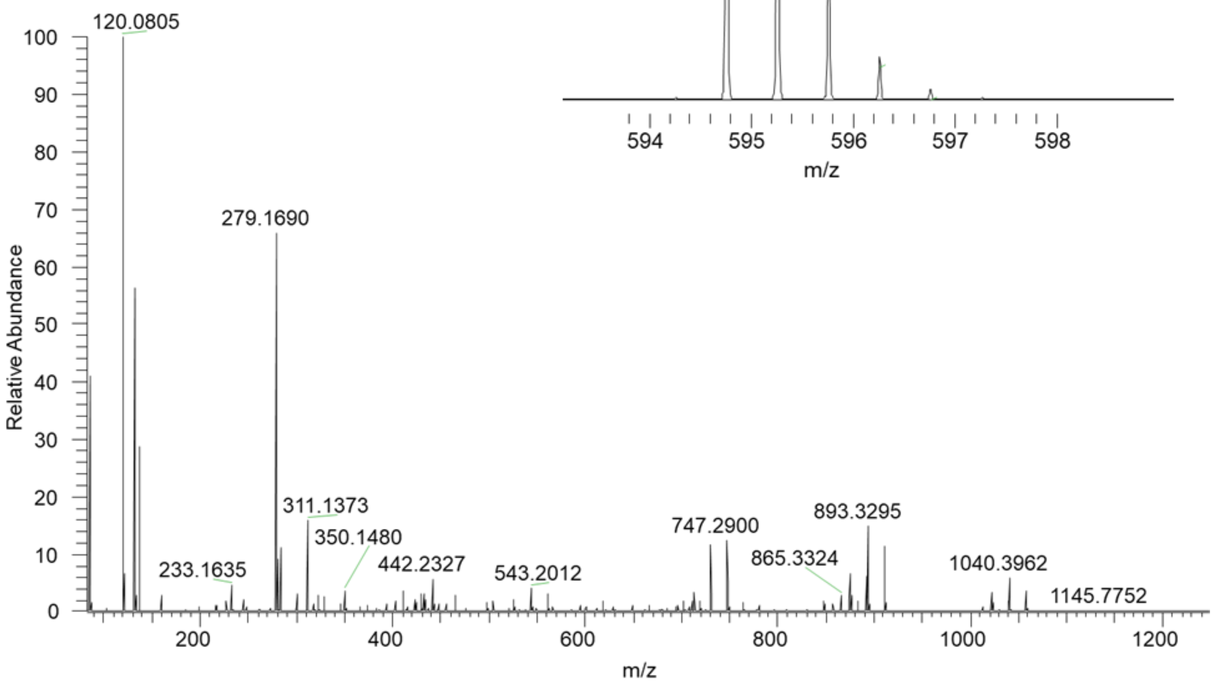

Figure S8. MS/MS spectrum for cleaved IndA core after incubation with SeIndP.

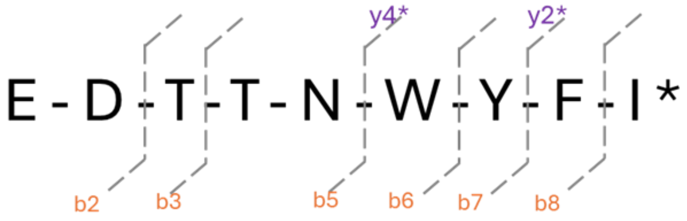

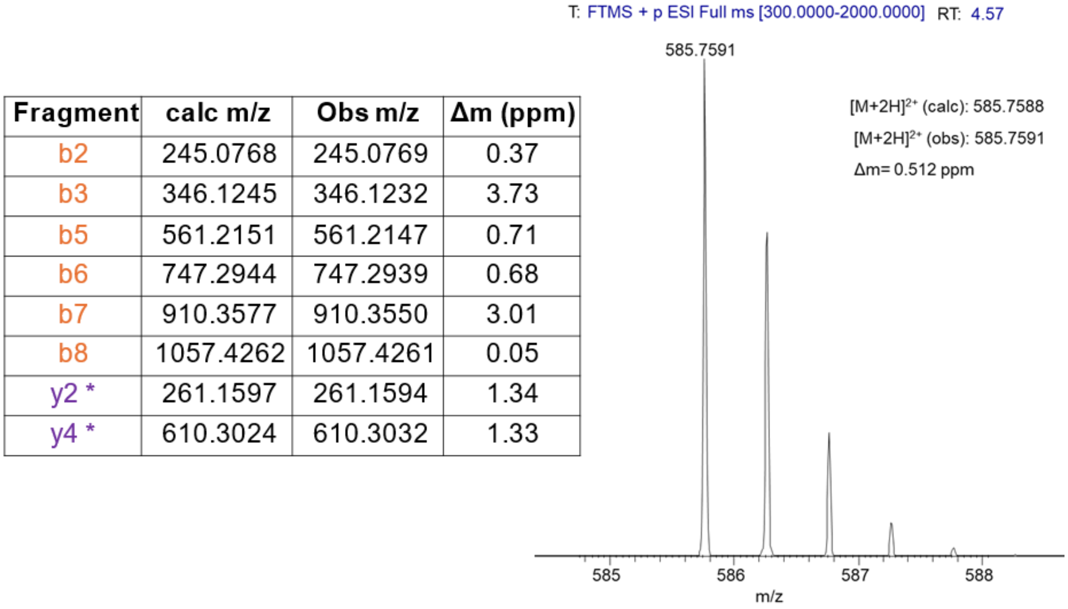

Fragment	calc m/z	Obs m/z	Δm (ppm)
b2	245.0768	245.0769	0.37
b3	346.1245	346.1232	3.73
b5	561.2151	561.2147	0.71
b6	747.2944	747.2939	0.68
b7	910.3577	910.3550	3.01
b8	1057.4262	1057.4261	0.05
y2 *	261.1597	261.1594	1.34
y4 *	610.3024	610.3032	1.33

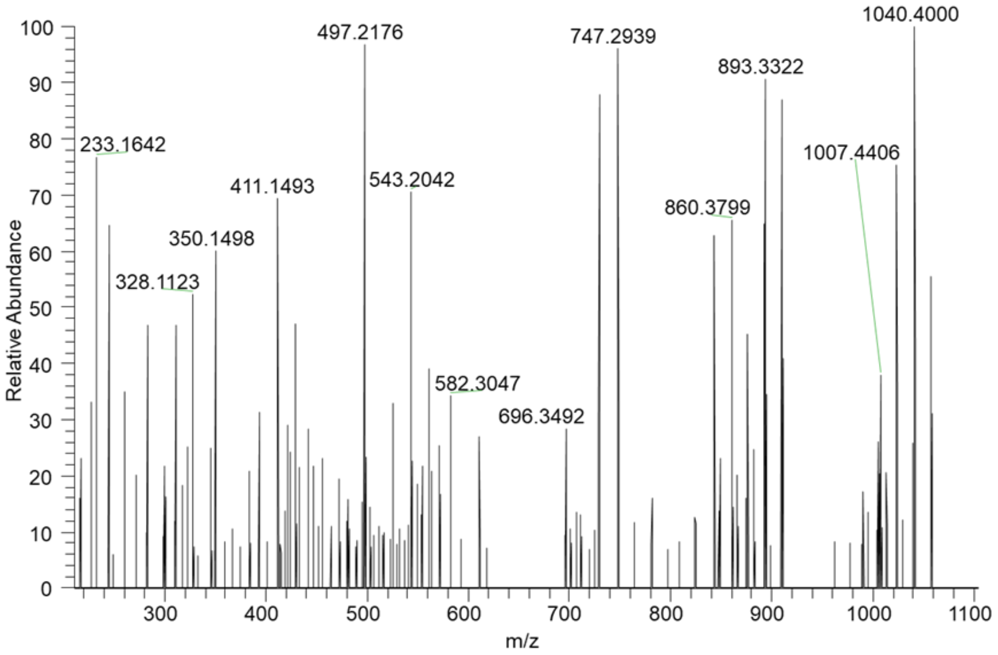

Figure S9. MS/MS spectrum for cleaved IndA-Cyclic core after incubation with IndF and SeIndP. Star (*) indicates the loss of one water in the MS/MS fragment ions.

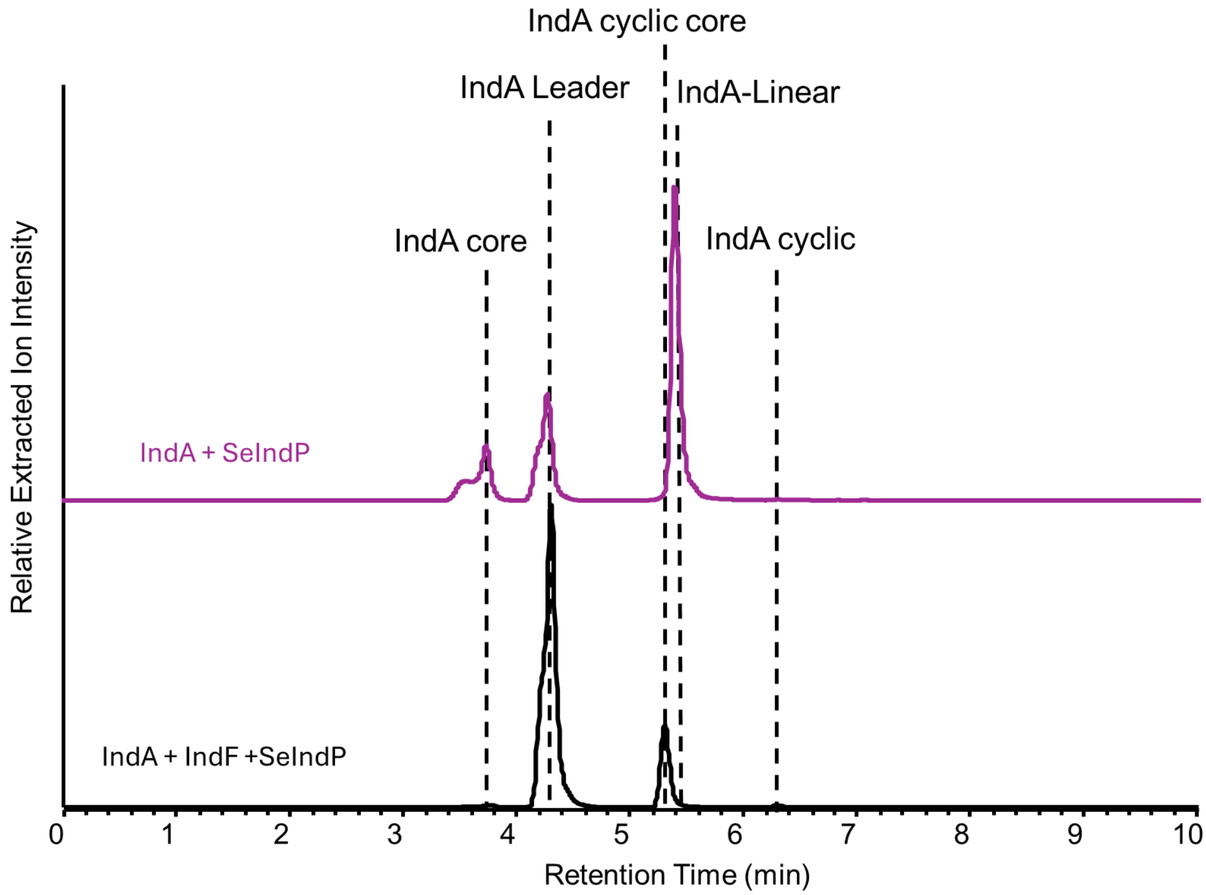

Figure S10. UHPLC-HRMS analysis of SeIndP cleavage of leader peptide from IndA (Top Trace) or IndA-Cyclic (Bottom Trace). Extracted ion chromatograms for each include IndA Leader: 1028.9822 *m/z* $[M+2H]^{2+}$, IndA linear core: 594.7640 *m/z* $[M+2H]^{2+}$, IndA: 1613.7319 $[M+2H]^{2+}$, IndA-Cyclic: 1604.7229 *m/z* $[M+2H]^{2+}$, IndA-Cyclic Core: 585.75791 *m/z* $[M+2H]^{2+}$ using a mass tolerance of 20 ppm.

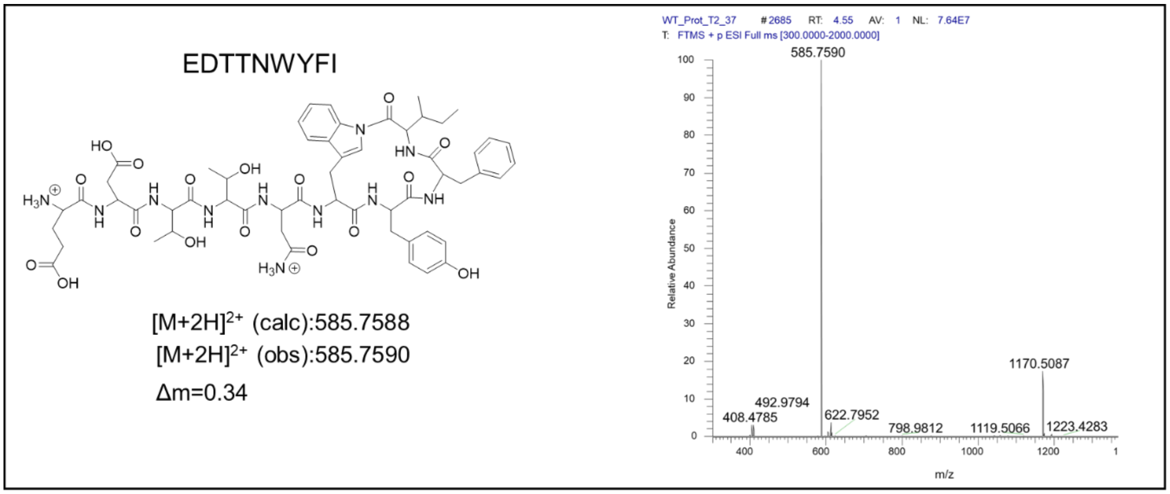

1 EDTTNWFI

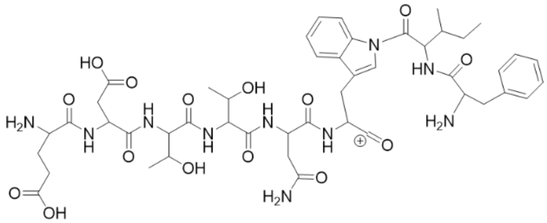

$[M+H]^+$ (calc): 1007.4469
$[M+H]^+$ (obs): 1007.4438
Δm= 3.03 ppm

2 EDTTNWYI

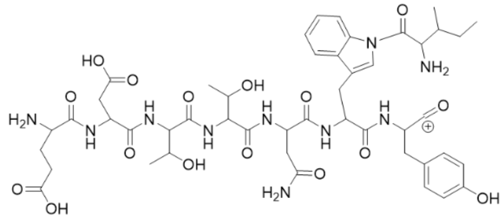

$[M+H]^+$ (calc): 1023.4418
$[M+H]^+$ (obs): 1023.4436
Δm= 1.76 ppm

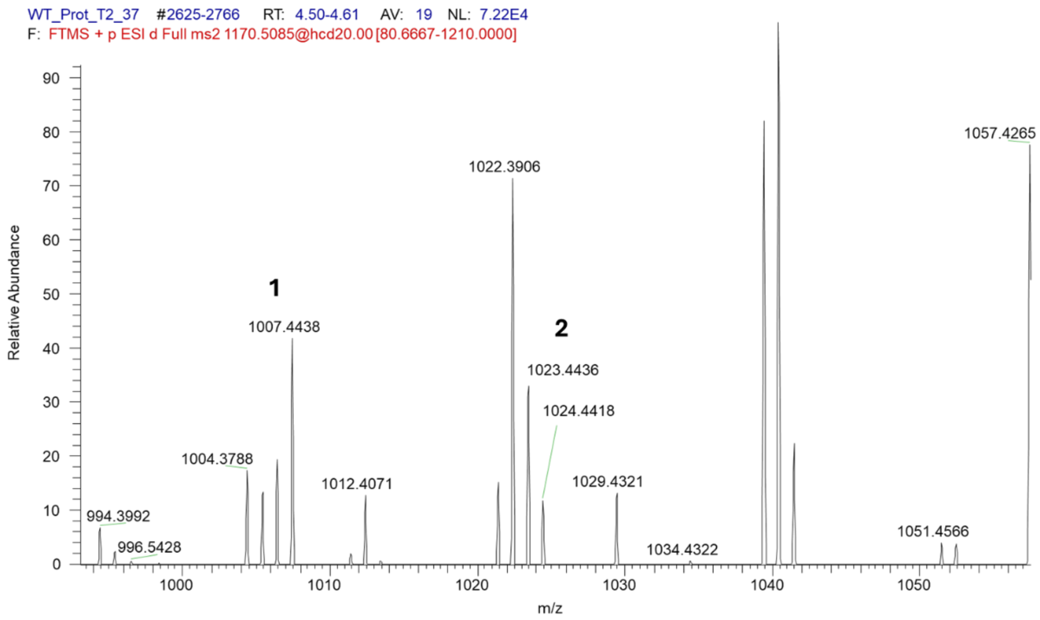

Figure S11. MS/MS fragmentation of IndA-Cyclic core, supporting W6 to I9 cyclization.

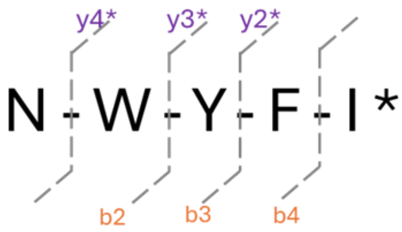

Fragment	calc m/z	Obs m/z	Δm (ppm)
b2	301.1295	301.1295	0.07
b3	464.1928	464.1925	0.75
b4	611.2613	611.2605	1.24
y2 *	261.1597	261.1600	0.96
y3 *	424.2231	424.2239	1.93
y4 *	610.3024	610.3030	1.00

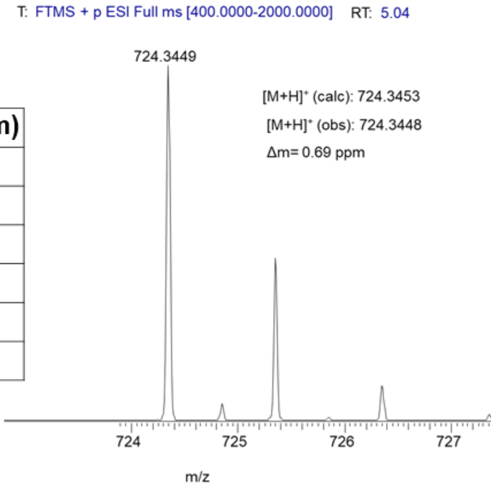

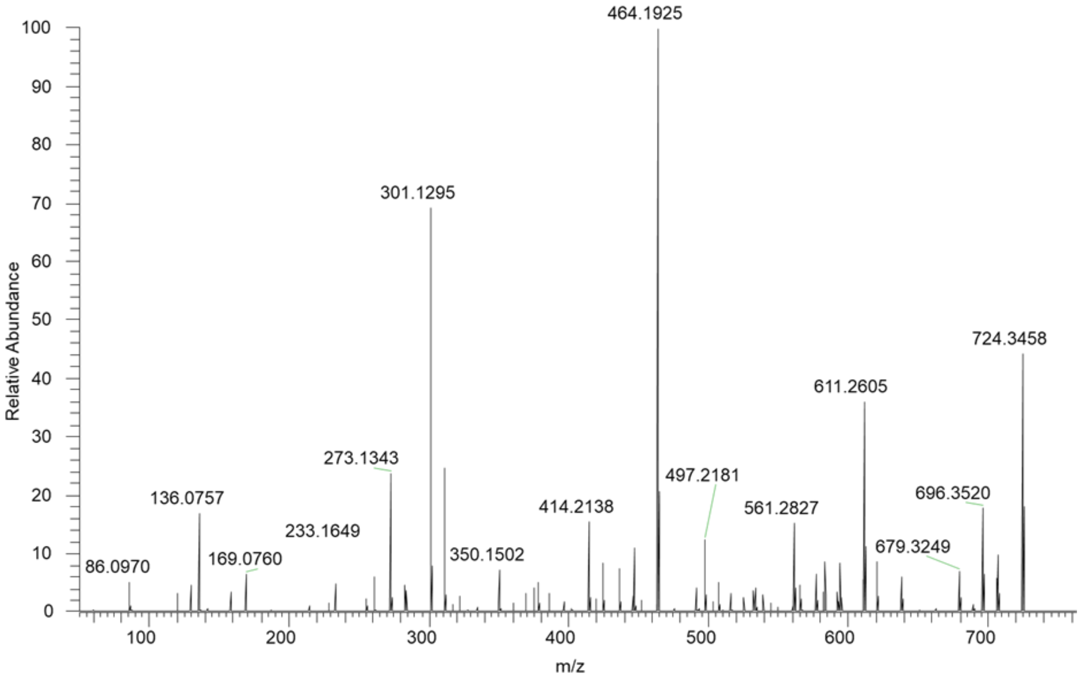

Figure S12. MS/MS spectrum of IndA-Cyclic$_{N5\text{-}I9}$ after cleavage by α-lytic protease. Star (*) indicates the loss of one water in the MS/MS fragment ions.

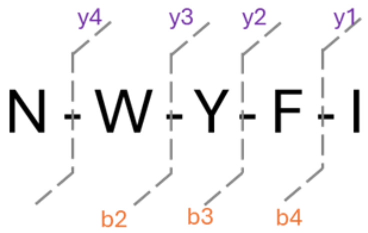

Fragment	calc m/z	Obs m/z	Δm (ppm)
b_2	301.1295	301.1295	0.07
b_3	464.1928	464.1925	0.75
b_4	611.2613	611.2620	1.21
y_1	132.1019	132.1015	3.33
y_2	279.1703	279.1701	0.90
y_3	442.2337	442.2335	0.41
y_4	628.3130	628.3129	0.14

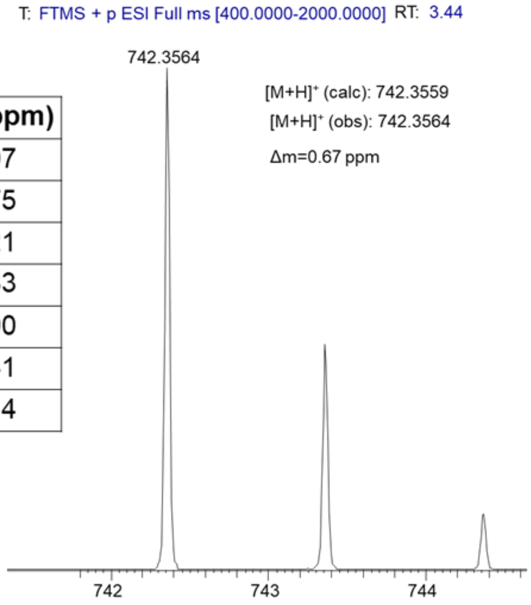

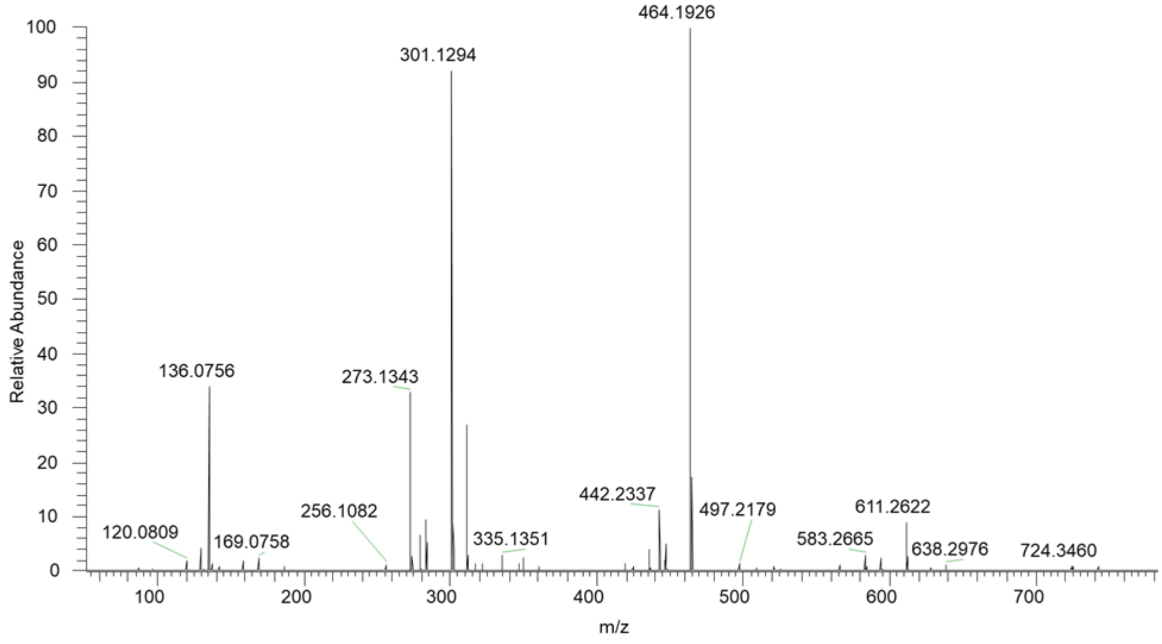

Figure S13. MS/MS spectrum of synthetic IndA$_{N5-I9}$.

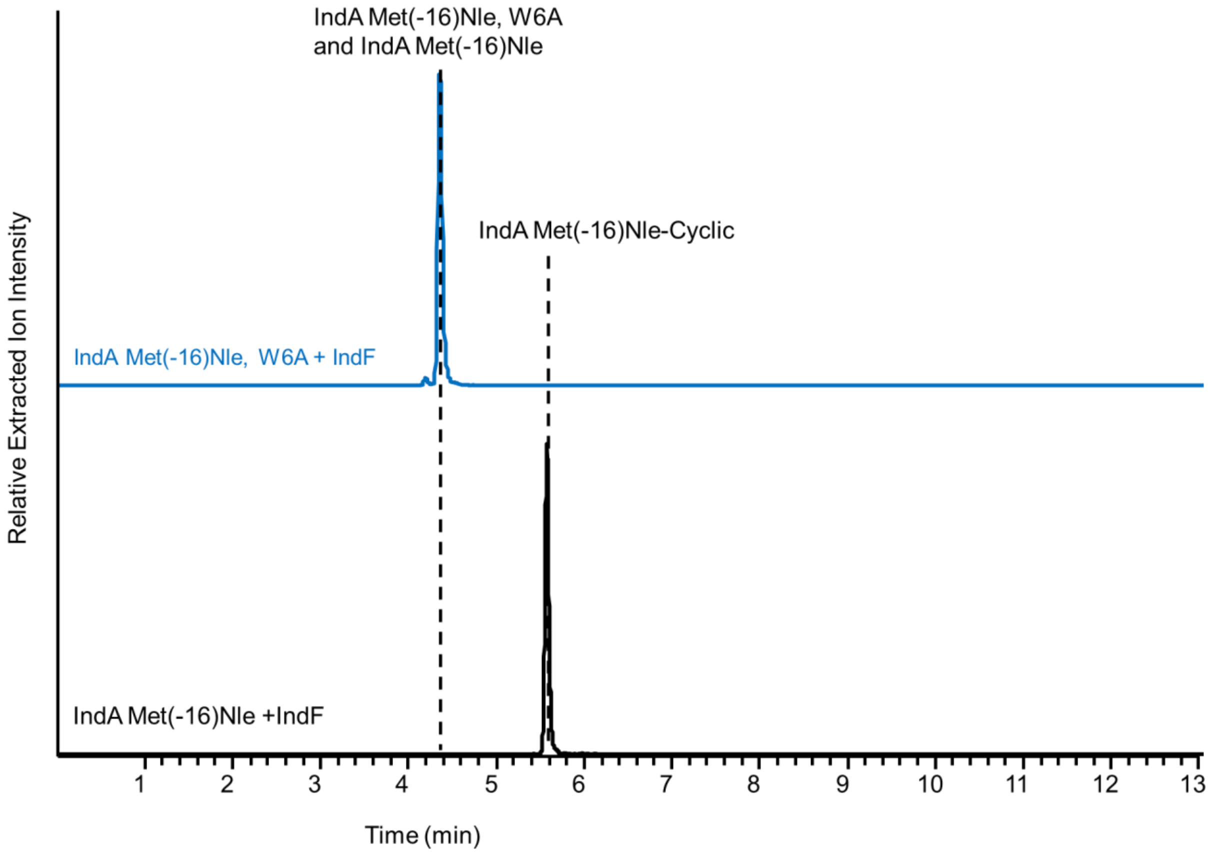

Figure S14. UHPLC-HRMS analysis of IndF cyclization of IndA variants. Extracted ion chromatograms of IndA Met(-16)Nle (1604.7568 *m/z* $[M+2H]^{2+}$) and IndA Met(-16)Nle-Cyclic (1595.7502 *m/z* $[M+2H]^{2+}$) after incubating with IndF (bottom) and IndA Met(-16)Nle,W6A (1547.2339 *m/z* $[M+2H]^{2+}$) after incubating with IndF (top) using a mass tolerance of 20 ppm.

The IndA Met(-16)Nle peptide was used to overcome issues with methionine oxidation.

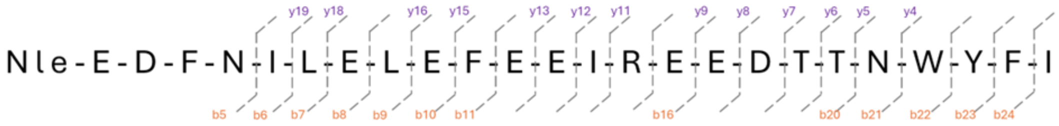

Fragment	calc m/z	Obs m/z	Δm (ppm)
b5	619.2722	619.2713	1.47
b6	732.3563	732.3569	0.86
b7	845.4403	845.4393	1.22
b8	974.4829	974.4809	2.07
b9	1087.5670	1087.5642	2.56
b10	1216.6096	1216.6068	2.28
b11	1363.6780	1363.6714	4.83
b16	2019.9909	2019.9864	2.24
b20	2466.1558	2466.1469	3.61
$b21^{+2}$	1290.6030	1290.5980	3.88
$b22^{+2}$	1383.6427	1383.6425	0.12
$b23^{+2}$	1465.1743	1465.1737	0.43
y4	628.3130	628.3133	0.49
y5	742.3559	742.3575	2.13
y6	843.4036	843.4024	1.42
y7	944.4513	944.4474	4.11
y8	1059.4782	1059.4806	2.25
y9	1188.5208	1188.5228	1.67
y11	1473.6645	1473.6607	2.59
y12	1586.7486	1586.7534	3.04
y13	1715.7912	1715.7855	3.30
y15	1991.9022	1991.9060	1.93
y16	2120.9448	2120.9429	0.87
y18	2363.0714	2363.0711	0.13
y19	2476.1555	2476.1482	2.93

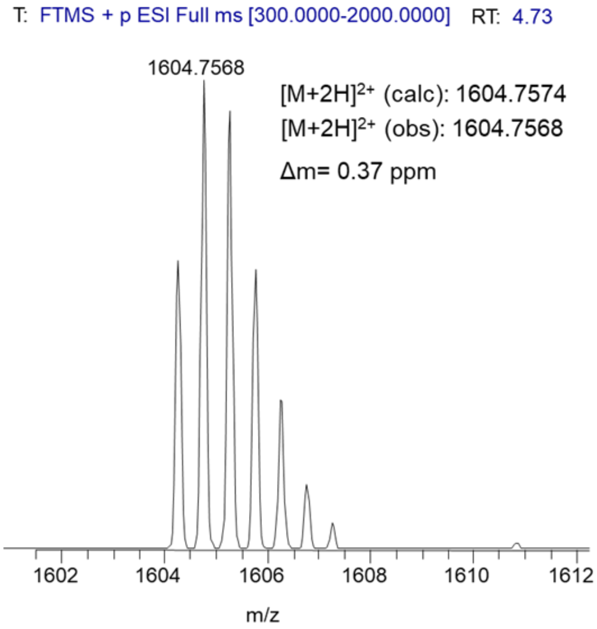

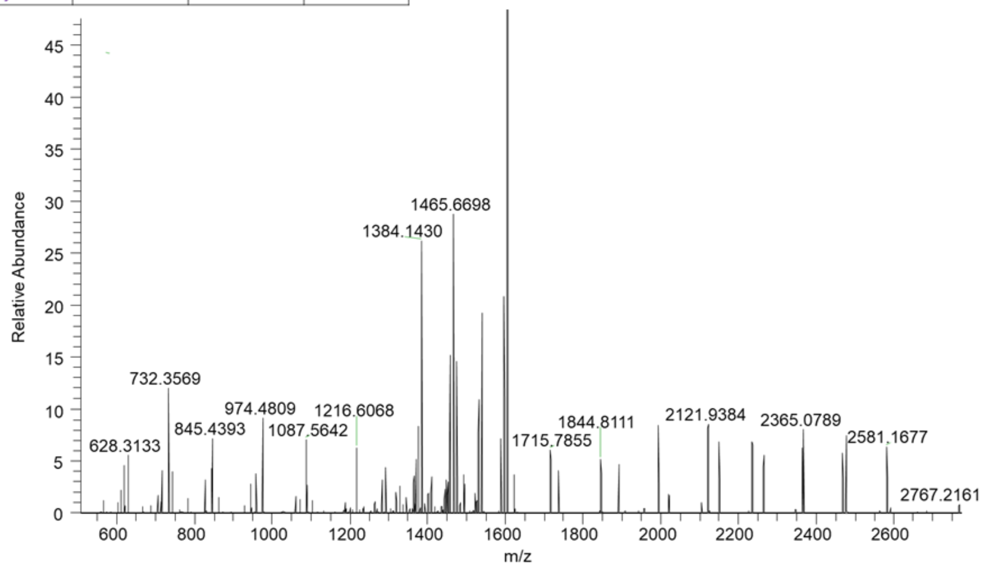

Figure S15. MS/MS spectrum of IndA Met(-16)Nle.

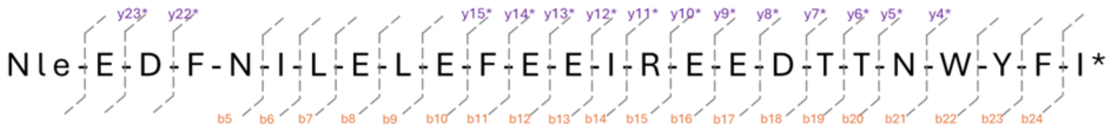

Fragment	calc m/z	Obs m/z	Δm (ppm)
b5	619.2722	619.2721	0.16
b6	732.3563	732.3569	0.86
b7	845.4403	845.4401	0.24
b8	974.4829	974.4808	2.80
b9	1087.5670	1087.5644	2.37
b10	1216.6096	1216.6073	1.87
b11	1363.6780	1363.6737	3.14
b12	1492.7206	1492.7175	2.06
b13	1621.7632	1621.7564	4.17
b14	1734.8472	1734.8387	4.91
b16	2019.9909	2019.9918	0.44
b17	2149.0335	2149.0397	2.88
b20^{+2}	1233.5815	1233.5763	4.25
b21^{+2}	1290.6030	1290.6093	4.87
b22^{+2}	1383.6427	1383.6371	4.02
b23^{+2}	1465.1743	1465.1672	4.87
y4*	610.3024	610.3029	0.84
y5*	724.3453	724.3455	0.25
y6*	825.3930	825.3913	2.06
y7*	926.4407	926.4394	1.34
y8*	1041.4676	1041.4659	1.65
y9*	1170.5102	1170.5055	4.02
y10*	1299.5528	1299.5491	2.85
y11*	1455.6539	1455.6503	2.48
y12*	1568.7380	1568.7365	0.94
y13*	1697.7806	1697.7769	2.16
y14*	1826.8232	1826.8174	3.15
y15*	1973.8916	1973.8925	0.48
y16*	2102.9342	2102.9437	4.54
y22^{+2} *	1416.6738	1416.6718	1.3900
y23^{+2} *	1474.1872	1474.1881	0.5800

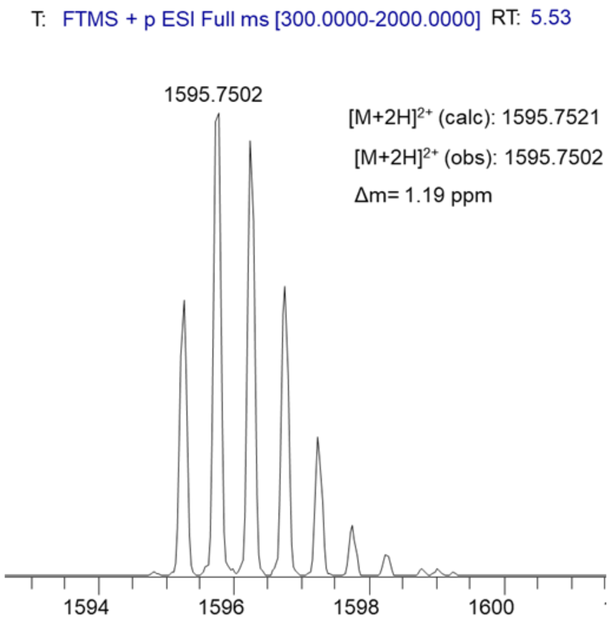

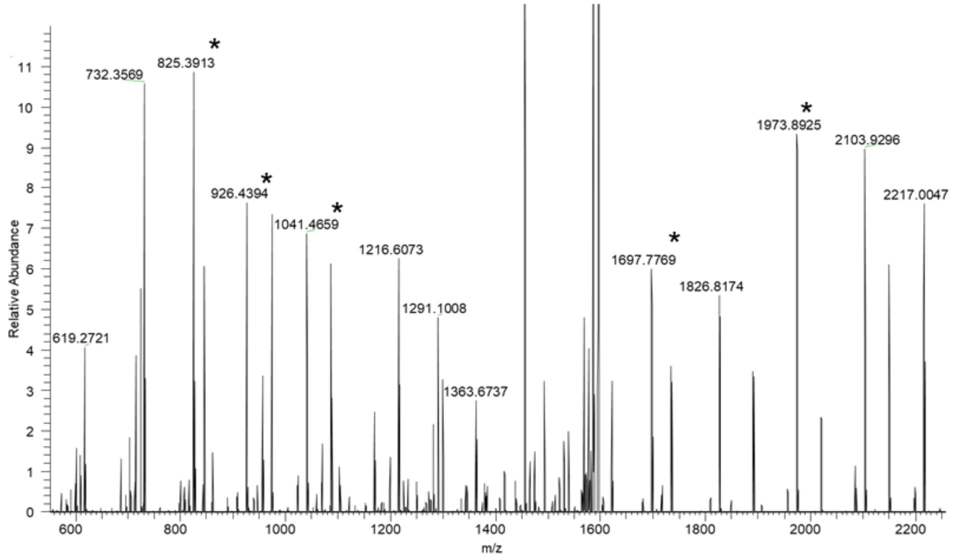

Figure S16. MS/MS spectrum of IndA-Cyclic Met(-16)Nle. Star (*) indicates the loss of one water in the MS/MS fragment ions.

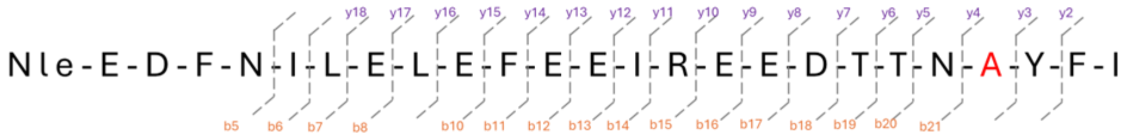

Fragment	Calc m/z	Obs m/z	Δm (ppm)
b_5	619.2722	619.2719	0.50
b_6	732.3563	732.3569	0.86
b_7	845.4403	845.4404	0.08
b_8	974.4829	974.4823	0.64
b_9	1087.5670	1087.5669	0.07
b_{10}	1216.6096	1216.6063	2.69
b_{11}	1363.6780	1363.6752	2.04
b_{12}	1492.7206	1492.7146	4.00
b_{13}	1621.7632	1621.7586	2.81
b_{14}	1734.8472	1734.8451	1.22
b_{15}	1890.9483	1890.9484	0.04
b_{16}	2019.9909	2019.9995	4.25
b_{17}	2149.0335	2149.0294	1.91
b_{18}	2264.0604	2264.0619	0.64
b_{19}	2365.1081	2365.0977	4.41
b_{21}	2580.1987	2580.1994	0.26
y_2	279.1703	279.1698	1.97
y_3	442.2337	442.2333	0.86
y_4	513.2708	513.2704	0.76
y_5	627.3137	627.3139	0.29
y_6	728.3614	728.3617	0.41
y_7	829.4091	829.4110	2.31
y_8	944.4360	944.4352	0.87
y_9	1073.4786	1073.4777	0.85
y_{10}	1202.5212	1202.5223	0.91
y_{11}	1358.6223	1358.6233	0.73
y_{12}	1471.7064	1471.7085	1.45
y_{13}	1600.7490	1600.7454	2.22
y_{14}	1729.7916	1729.7903	0.72
y_{15}	1876.8600	1876.8569	1.63
y_{16}	2005.9026	2005.9083	2.87
y_{17}	2118.9866	2118.9891	1.18
y_{18}	2248.0292	2248.0232	2.67

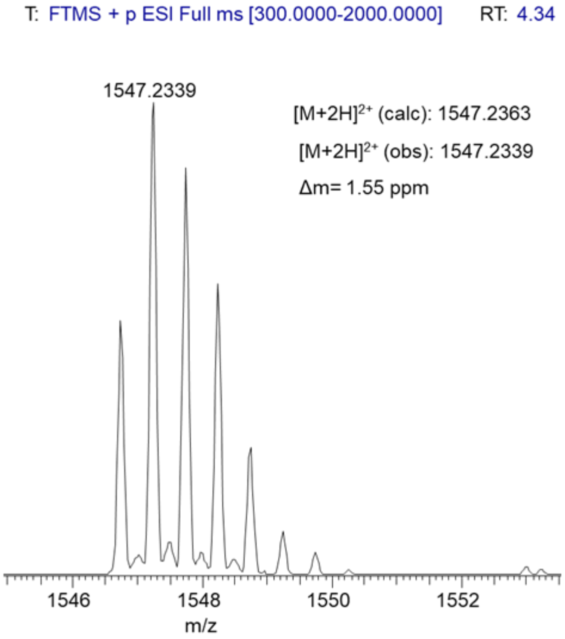

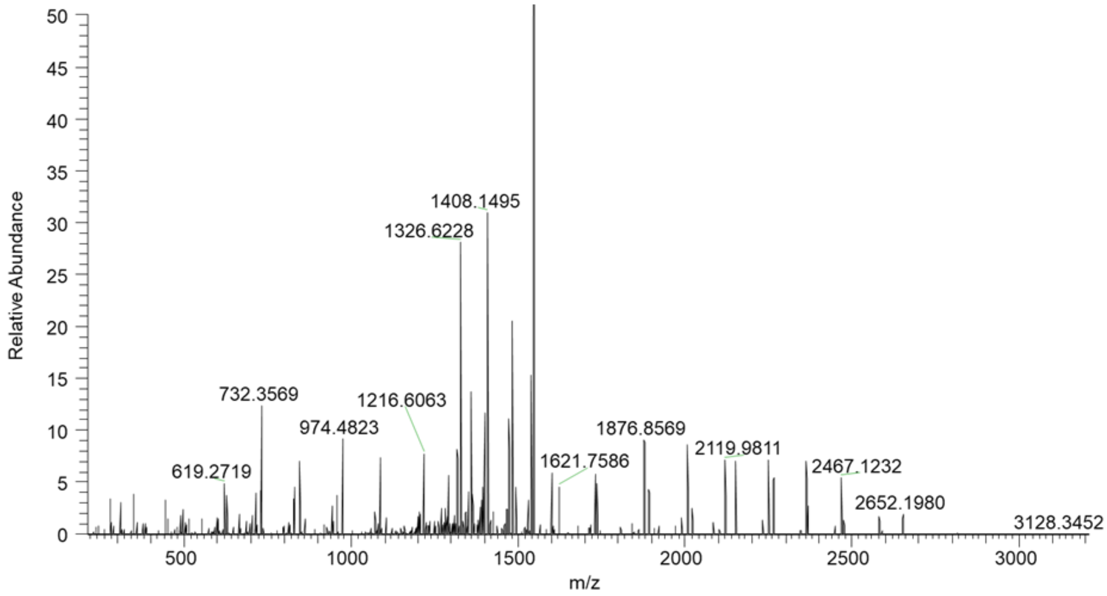

Figure S17. MS/MS spectrum of IndA Met(-16)Nle+W6A.

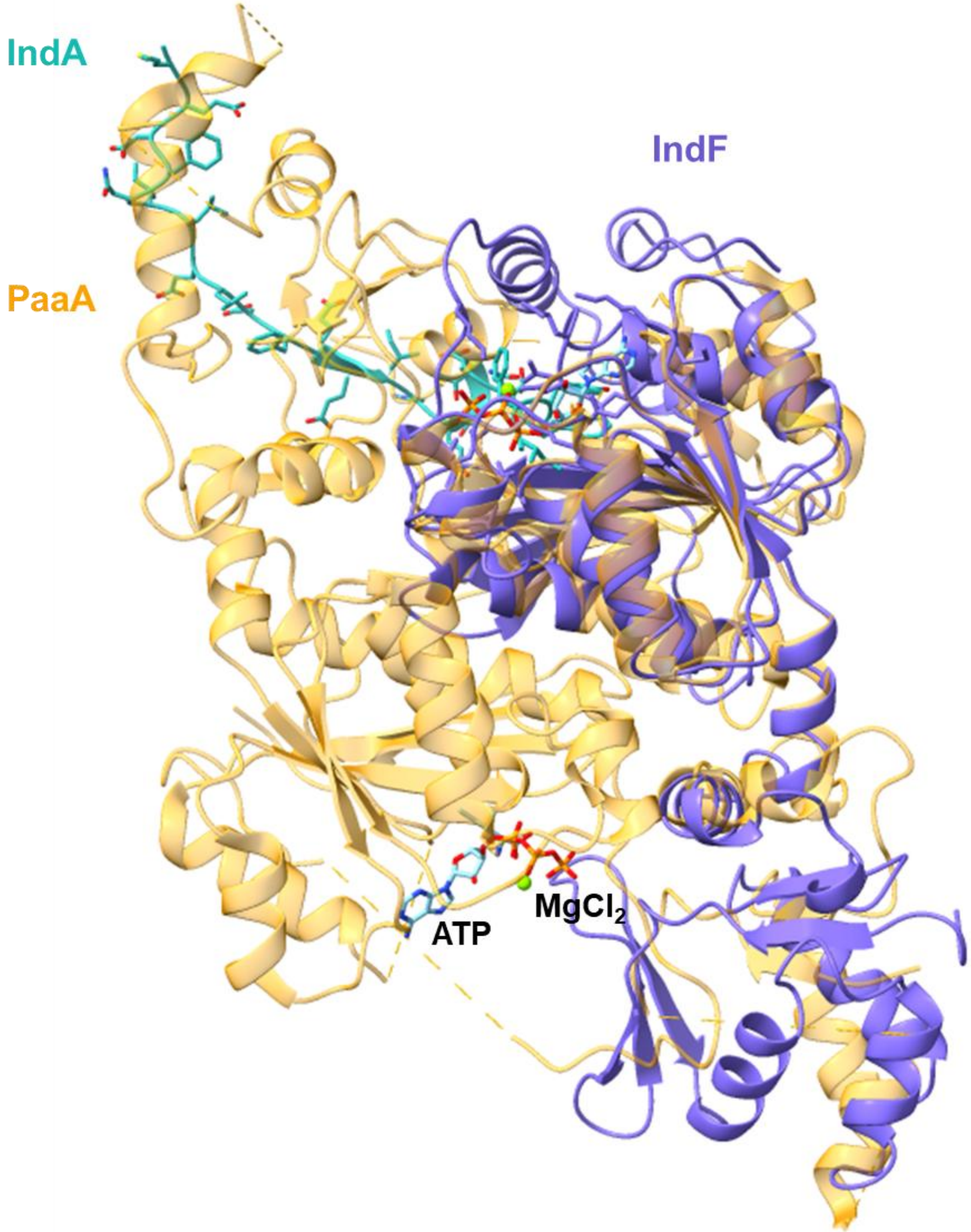

Figure S18. PaaA and IndF structure comparison: PaaA structure (orange-PDB:5FF5) and IndF monomer (purple) with ATP and IndA substrate (teal) docking. RMSD of 3.44 Å with 263 aligned residues.

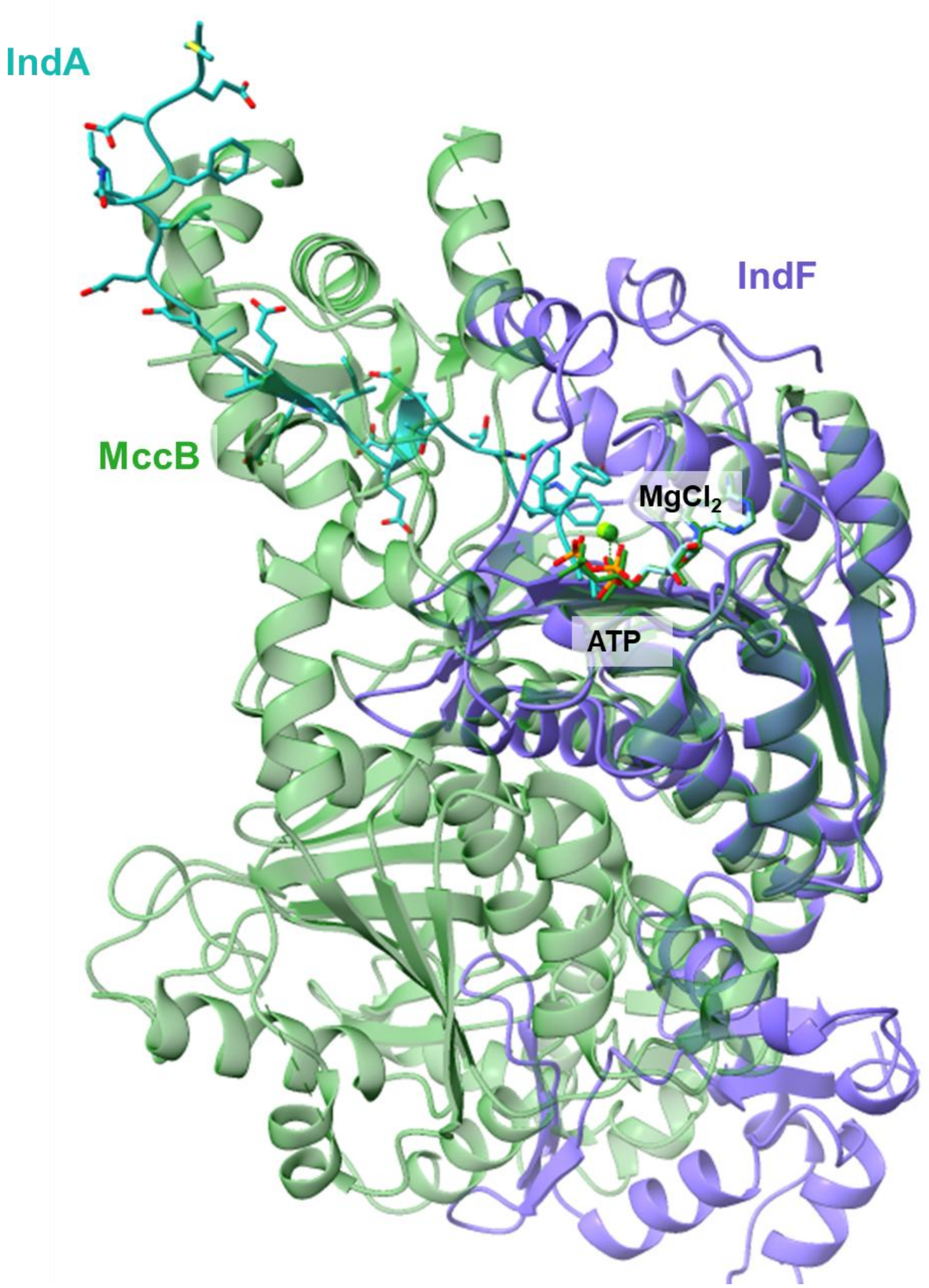

Figure S19. MccB and IndF Structure comparison: MccB structure (green-PDB: 3H5N) overlay with IndF monomer (purple) with ATP and IndA (teal) docking, RMSD of 4.34 Å with 246 residues aligned.

```
IndF/1-373     1 MDN--YFPKWNQDRIVYQWKNDRLRIGADDVDVLEITGYSD-FWSD-LISCCNGINSFEEIKDLLRKKY-DISENII  72
EnfB/1-375     1 MSY--ISPRVKPIYPLYKLNSNIFRIGAQLGITTEIEEEDNQFWT--LANLLNGKNKLDHIITSMQKKYPELTEQNI  73
MccB/1-350     1 MDY-----ILGRYVKIARYGSGGLVGGG--GKEQYVENLVL--WEN--IIKTAYCFITPSSYTAAL-ETANIPEKDF  65
PaaA/1-373     1 MSLTNVKPLIKESHHIILADDGDICIGEIPGVSQVINDPPS--WVRPALAKMDGKRTVPRIFKELVSEGVQIESEHL  75
GrcB/1-362     1 MGVY----VKNKLCYIIFQEQSILVVNNDIAKYYVFEDSPV---LNCLLSALENNILTNDKLDRISQQL----SENI  66

IndF/1-373    73 EKYISKFSDRNLLEILDR----------PVNQIDHYLINESLETYYSSEGIGGIKLLEKLSNLKVTILGCGAGGSHI 139
EnfB/1-375    74 VDGINLLNDEGVLEESLP----------NKATEERYLPNVNYFSRFIDTSGDRFLIQKEINETKILLLGLGGGGSNI 140
MccB/1-350    66 SNCF------RFLKENFF---IIPGEYNNSTENNRYSRNFLHYQSYGAN---PVLVQDKLKNAKVVILGCGGIGNHV 130
PaaA/1-373    76 EGLVAGLAERKLLQDNSFFSKVLSGE-----EVERYNRQILQFSLIDADNQHPFVYQERLKQSKVAIFGMGGWGTWC 147
GrcB/1-362    67 DILFKVLNELIKLKIVFYVDETEEEFNINHITDERFSTEILYLNKFTSK---PYQLLQAIQSKRVVIIGAGALGSSL 140

IndF/1-373   140 ALQLAQLGVGRLHLVDDDIVKENNINRQSMFTFNDI--GKYKVDCVKDCILKRNYQCVVTKR-KLKMSTVDAVKKEI 213
EnfB/1-375   141 LTLLAGLGPKSIKIVDYDIVELGNLGRQFLYRENDI--GKKKTEIAKSAVEKMNSLIKIEAF-DKKIVVVEDVLELI 214
MccB/1-350   131 SVILATSGIGEIILIDNDQIENTNLTRQVLFSEDDV--GKNKTEVIKRELLKRNSEISVSEI-ALNINDYTDLHKVP 204
PaaA/1-373   148 ALQLAMSGIGTLRLIDGDDVELSNINRQVLYRTDDV--GKNKVDAAKDTILAYNENVHVETFFEFASPDRARLEELV 222
GrcB/1-362   141 ALKISAIGVRDIICIDGDKIELDNLTRQILYTPGDIEKNLFKVSALESRIKDFFPSSQFQGIKSYITSYDDCVNYIP 217

IndF/1-373   214 SESDWVF---CCMDEPPYI-AQRLVNRACYLFNIPSIYCFSQRSAGKL--LFCNPNIQNIGCVDC------LLYEQD 278
EnfB/1-375   215 NDVDIVV---CAIDEPPFI-IHRIVNEAIVKAGIPCVFGASQVSRGRV--YSVVPRVT--GCFDCLN----LHFSKN 279
MccB/1-350   205 EADIWVV---SADHPF---NLINWVNKYCVRANQPYINAGYVNDIAVFGPLYVPG--KT-GCYECQKVVADLYGSEK 272
PaaA/1-373   223 GDSTFIILAWAALGYYRKDTAEEIIHSIAKDKAIPVIELGGDPLEISVGPIYLNDGVHS-GFDEVKNSVKDKYYDSN 298
GrcB/1-362   218 HNSDLII---QTADYPIGK-LDDWINKVSLKYNIPVIFSHF----GSVGPFLIPS--ET-GCLRCLDQFLDTKTNGL 283

IndF/1-373   279 SD-----NFQNLVKKFSNYDG--KLITANILTNILLLSSWVVKKWLDCVTE----KNSNVWNTLFRFDFYSFREDEF 344
EnfB/1-375   280 DS-----KFIEQFIGFRNINF--NPPSIAYGPGIFQLVAGIVDEVVRIITQYAPPRSLGTQYEINYEDGSSFCHPSW 349
MccB/1-350   273 ENIDHKIKLINSRFKPATF-----------APVNNVAAALCAADVIKFIGKYSEPLSLNKRIGIWSDEIKIHSQNMG 338
PaaA/1-373   299 SDIRK---FQEARLKHSFIDGDRKVNAWQSAPSLSIMAGIVTDQVVKTITGYDKPHLVGKKFILSLQDFRSREEEIF 372
GrcB/1-362   284 NSITK--NFFQNIPNSKSPSF----------VTGTQLNELLILDIIKKFWISDEYRDFYNKVYTFYDGFSDIKTLIF 348

IndF/1-373   345 KHFSKQSHCPTCG---HDFDKSKLWEILKIDE-                                             373
EnfB/1-375   350 PRFA--SECPTCGAGNHDD-----WAIFQYYKQ                                             375
MccB/1-350   339 RSPV----CSVCG------------------NRM                                            350
PaaA/1-373   373 K--------------------------------                                             373
GrcB/1-362   349 PTVE---NC-ICRRNYHE---------------                                             362
```

Figure S20. Sequence alignment of IndF, EnfB, MccB, PaaA, and GrcB.

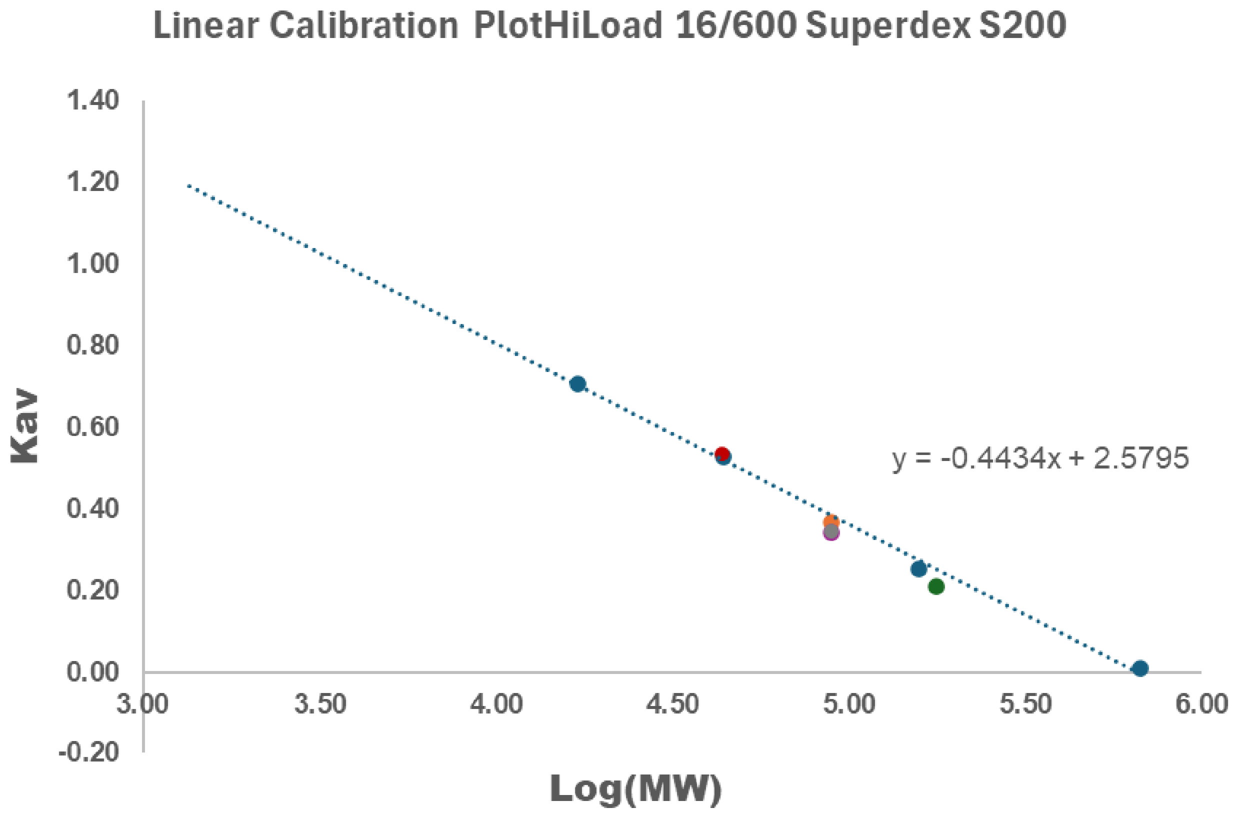

- MW [MBP-IndF]-dimer$_{GF}$ = 222.75 kDa; MW [MBP-IndF]-dimer$_{Th}$ = 178.02 kDa
- MW [MBP-IndF]-monomer$_{GF}$ = 98.26 kDa; MW [MBP-IndF]-monomer$_{Th}$ = 89.01 kDa
- MW [IndF]$_{GF}$ = 39.74 kDa; MW [IndF]$_{Th}$ = 43.61 kDa
- MW [MBP-IndF:IndA]$_{GF}$ = 108.09 kDa; MW [MBP-IndF:IndA]$_{Th}$ = 89.01 kDa
- MW [MBP-IndF+ATP+$MgCl_2$]$_{GF}$ = 109.70 kDa; MW [MBP-IndF+ATP+$MgCl_2$]$_{Th}$ = 89.01 kDa

Figure S21. Linear calibration plot for HiLoad 16/600 Superdex S200 size exclusion chromatography column. The MW standards are in blue. GF is calculated molecular weight from elution time and Th is theoretical molecular weight.

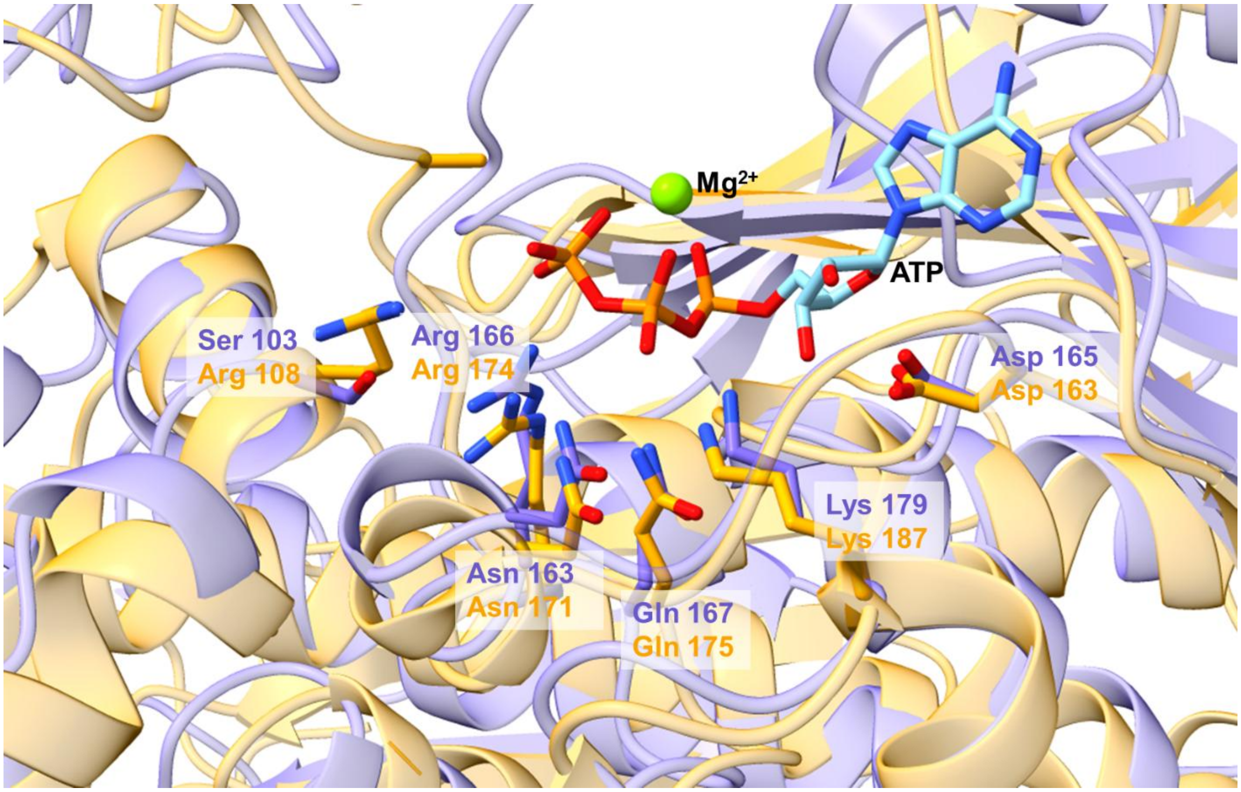

Figure S22. Structure overlay of PaaA and IndF ATP Binding Domains: PaaA (PDB: 5FF5) ATP binding domain (orange) with IndF ATP binding domain (purple). ATP and magnesium shown are from the IndF model.

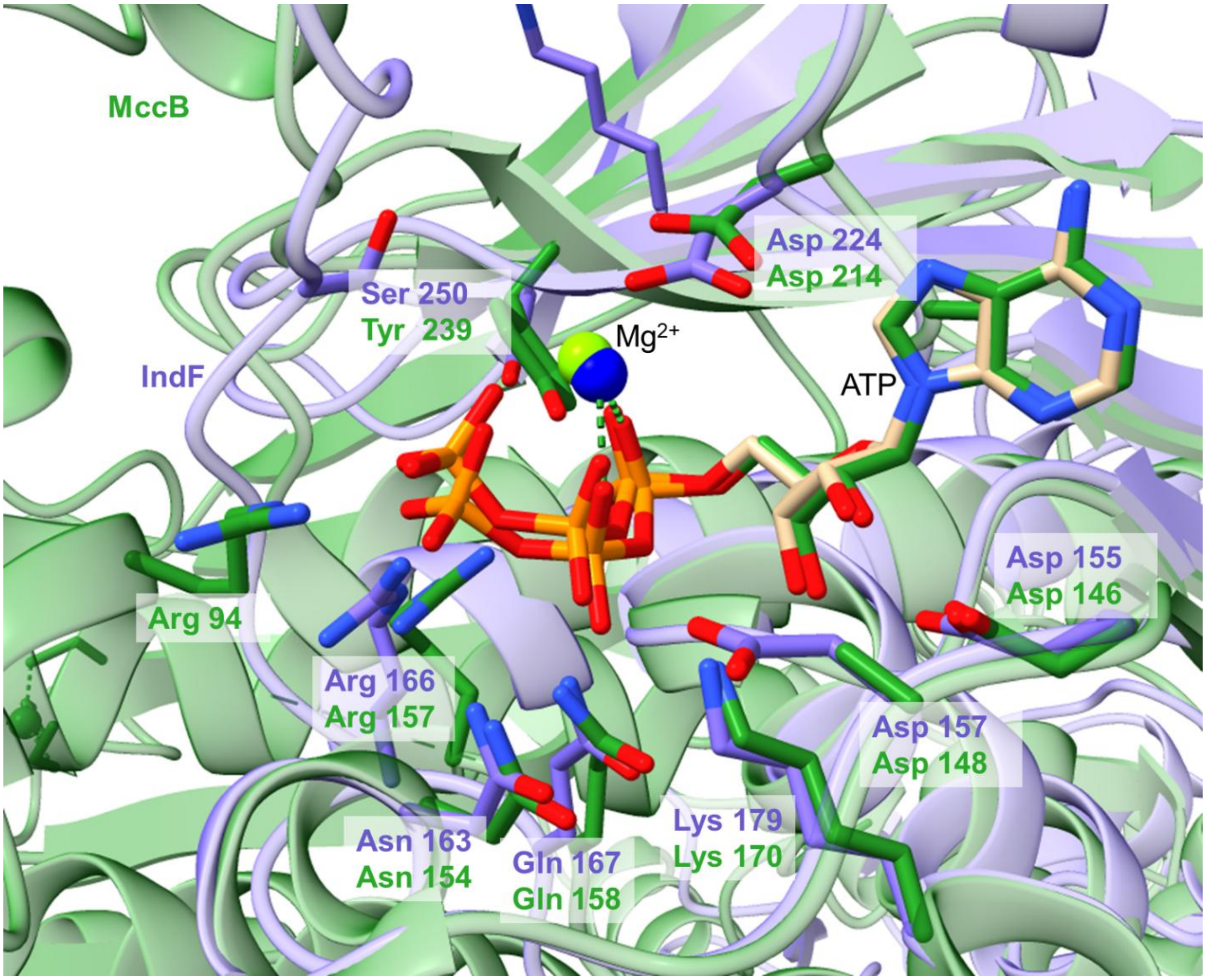

Figure S23. Structure overlay of MccB and IndF ATP Binding Domains: MccB (PDB: 3H5N) ATP binding domain (green) with IndF ATP binding domain (purple). ATP and magnesium shown are from both the MccB structure and IndF model.

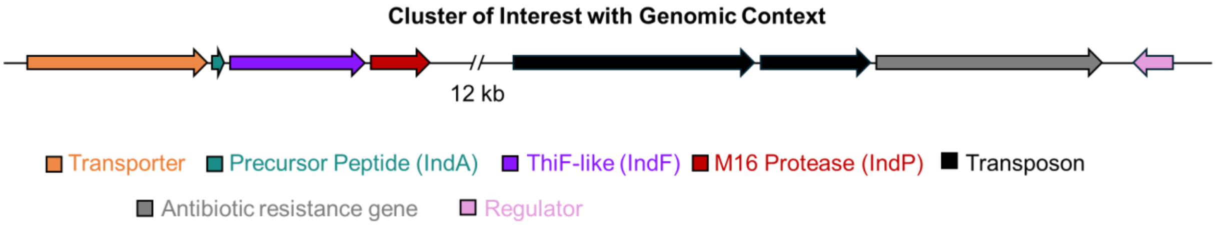

Figure S24. Genomic context for IndF biosynthetic gene cluster (GenBank assembly CP002176.1).

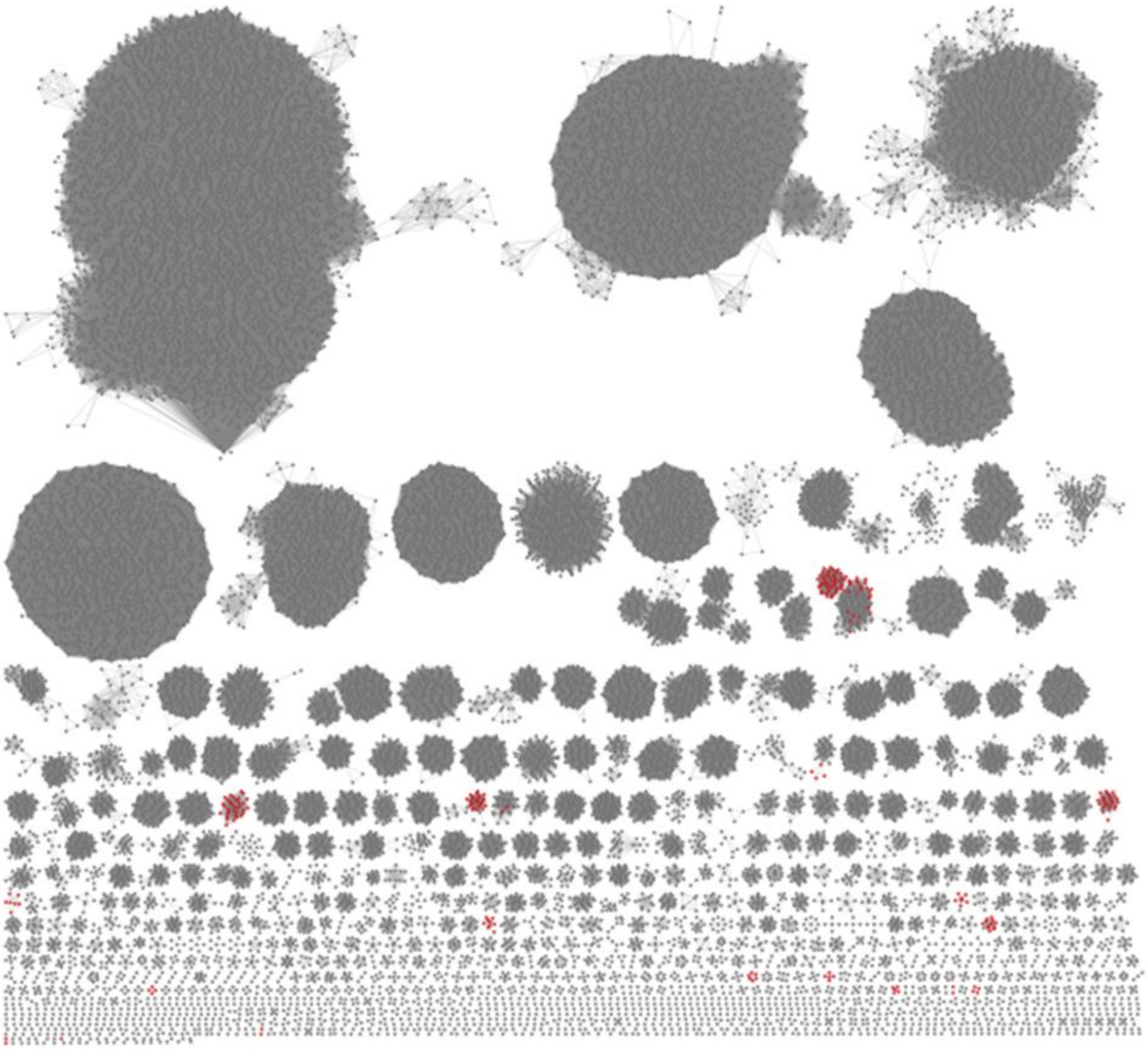

Figure S25. Sequence Similarity Network with Streptococcus ThiF-like genes highlighted in red.

Table S2. NMR table for IndA$_{N5\text{-}I9}$

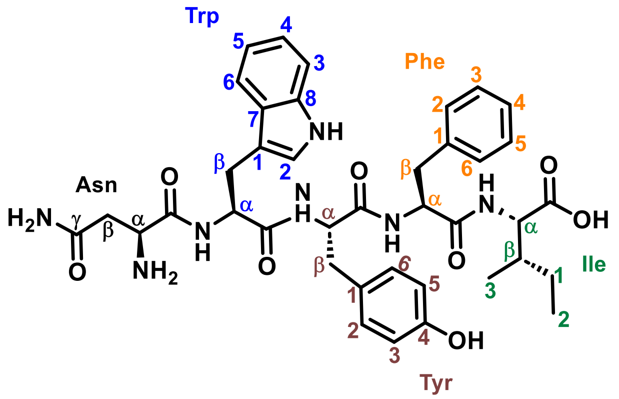

Residue	H	δ(^{1}H) (*J*, d) [ppm]	C	δ(^{13}C) [ppm]
**Asn**	Hα	3.48 (dd, *J* = 8.7, 4.4 Hz)	Cα	51.5
	Hβ, Hβ	2.43 (dd, J = 15.2, 4.3 Hz), 2.13 (dd, *J* = 15.2, 8.7 Hz)	Cβ	39.6
**Trp**				
	Hα	4.48 – 4.41 (m)	Cα	53.3
	Hβ	3.03 (overlap), 2.88 (overlap)	Cβ	27.7
	-	-	C1	109.8
	H2	7.06 (d, *J* = 2.4 Hz)	C2	123.6
	H3	7.30 (d, *J* = 8.1 Hz)	C3	111.2
	H4	7.05 – 7.01 (m)	C4	120.8
	H5	6.92 (m)	C5	118.4
	H6	7.52 (d, *J* = 7.9 Hz)	C6	118.1
	-		C7	127.4
	-		C8	136.01
	-NH	8.19 (d, J = 7.8 Hz)		
	-NH (Indole)	10.75 (d, *J* = 2.4 Hz)		
**Tyr**				
	Hα	4.36 (td, *J* = 8.6, 4.5 Hz,)	Cα	54.5
	Hβ	2.93 – 2.77 (overlap), 2.67 (dd, *J* = 14.0, 9.2 Hz)	Cβ	36.5
	-		C1	127.8
	H2, H6	6.99 – 6.87 (overlap)	C2, C6	130.1
	H3, H5	6.59 (d, *J* = 8.4 Hz)	C3, C5	114.8
	-		C4	155.6
			-OH	9.14, bs
	-NH	8.08 (overlap)		

Phe	Hα	4.61 (td, J = 8.7, 4.6 Hz)	$C_α$	56.7
	Hβ	3.08 (overlap), 2.84 (overlap)	$C_β$	37.4
			C1	137.6
	H2, H6	7.25 (overlap)	C2, C6	129.1
	H3, H5	7.25 (overlap)	C3, C4	128.0
	H4	7.19 – 7.15 (m)	C5	126.1
	-NH	8.06 (overlap)		
Ile	Hα	4.15 (dd, *J* = 8.3, 5.7 Hz)	$C_α$	56.7
	Hβ	1.87 – 1.72 (m)	$C_β$	36.6
	H1	1.49 – 1.38 (m), 1.25–1.15 (m)	C1	24.7
	H2	0.90 – 0.76 (m, overlap)	C2	11.4
	H3	0.90 – 0.76 (m, overlap)	C3	15.6
	-NH	7.92 (d, *J* = 8.3 Hz)		

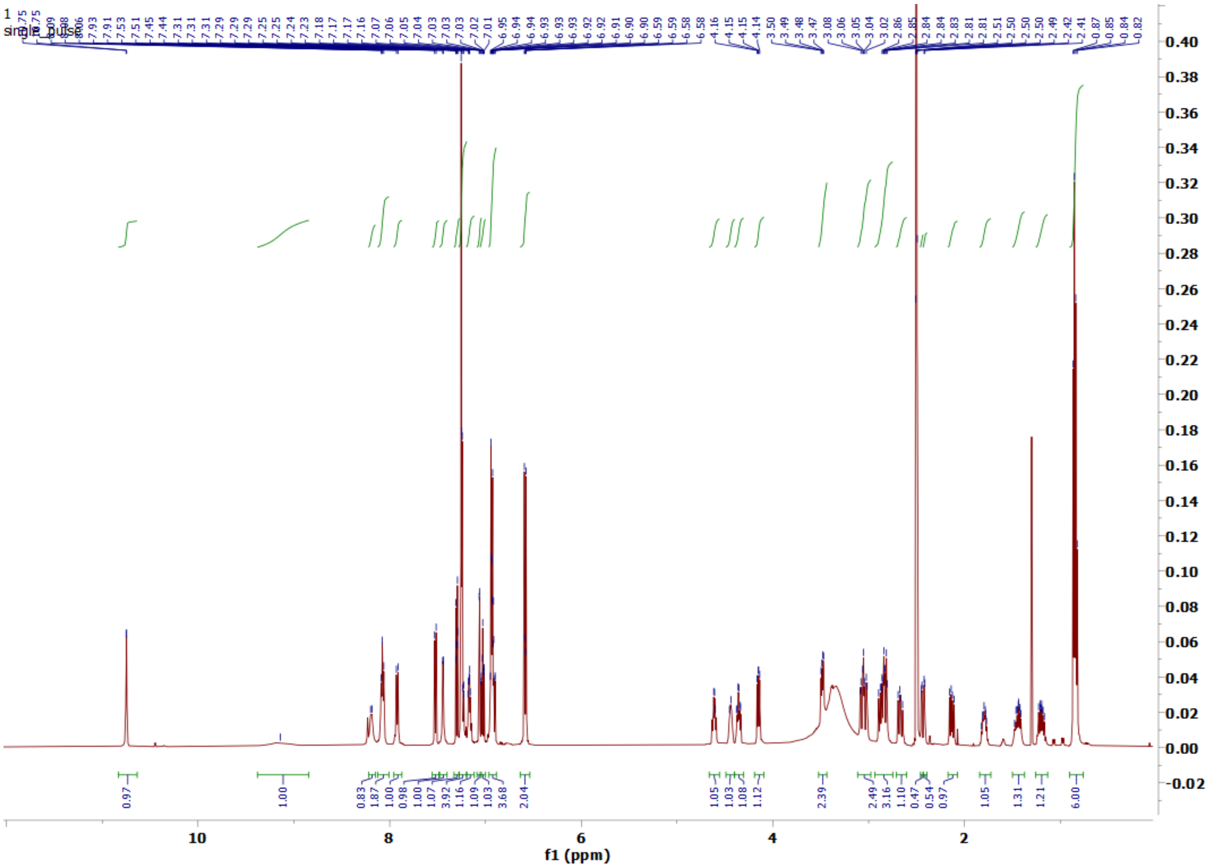

Figure S26. ^{1}H-NMR spectrum of IndA$_{N5\text{-}I9}$ in DMSO-d_6 (500 MHz).

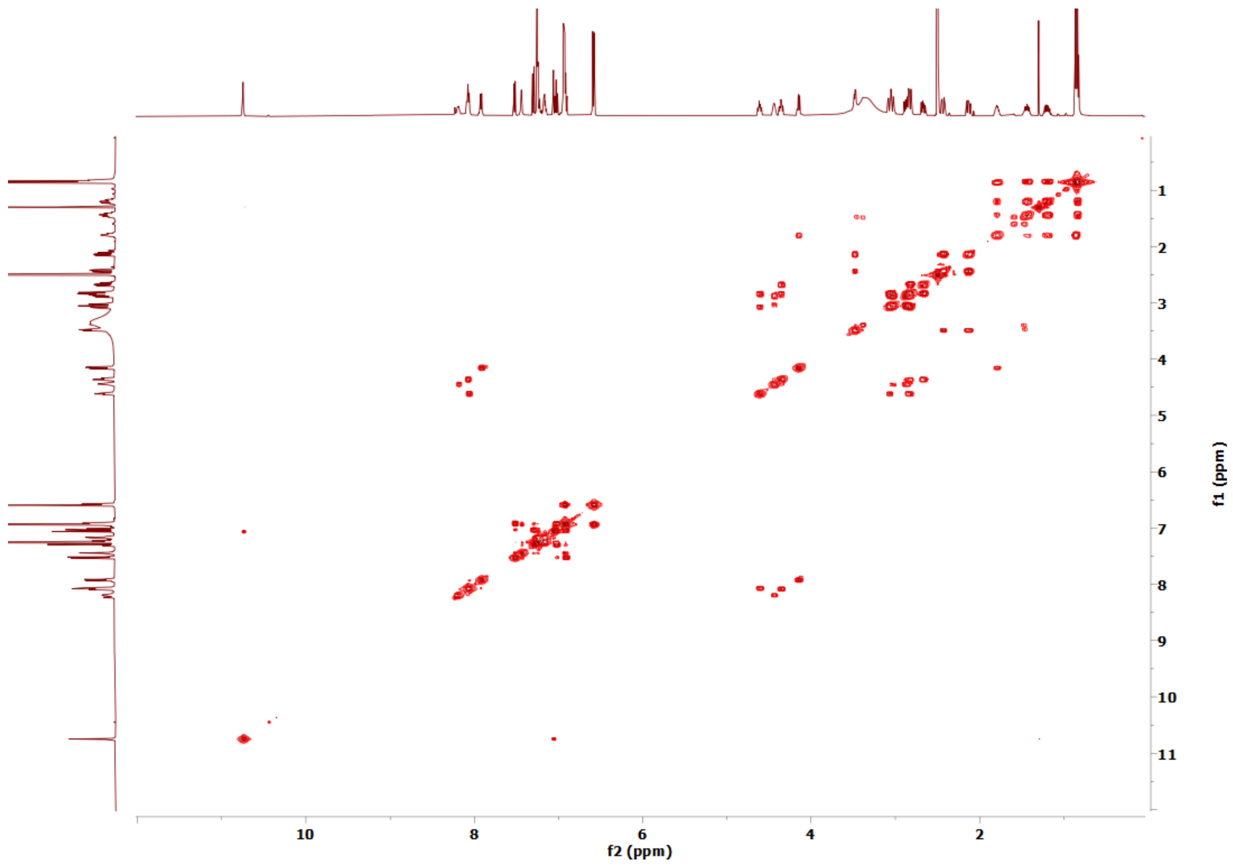

Figure S27. ^{1}H-^{1}H-COSY NMR spectrum of IndA$_{N5-I9}$ in DMSO-d_6 (500 MHz).

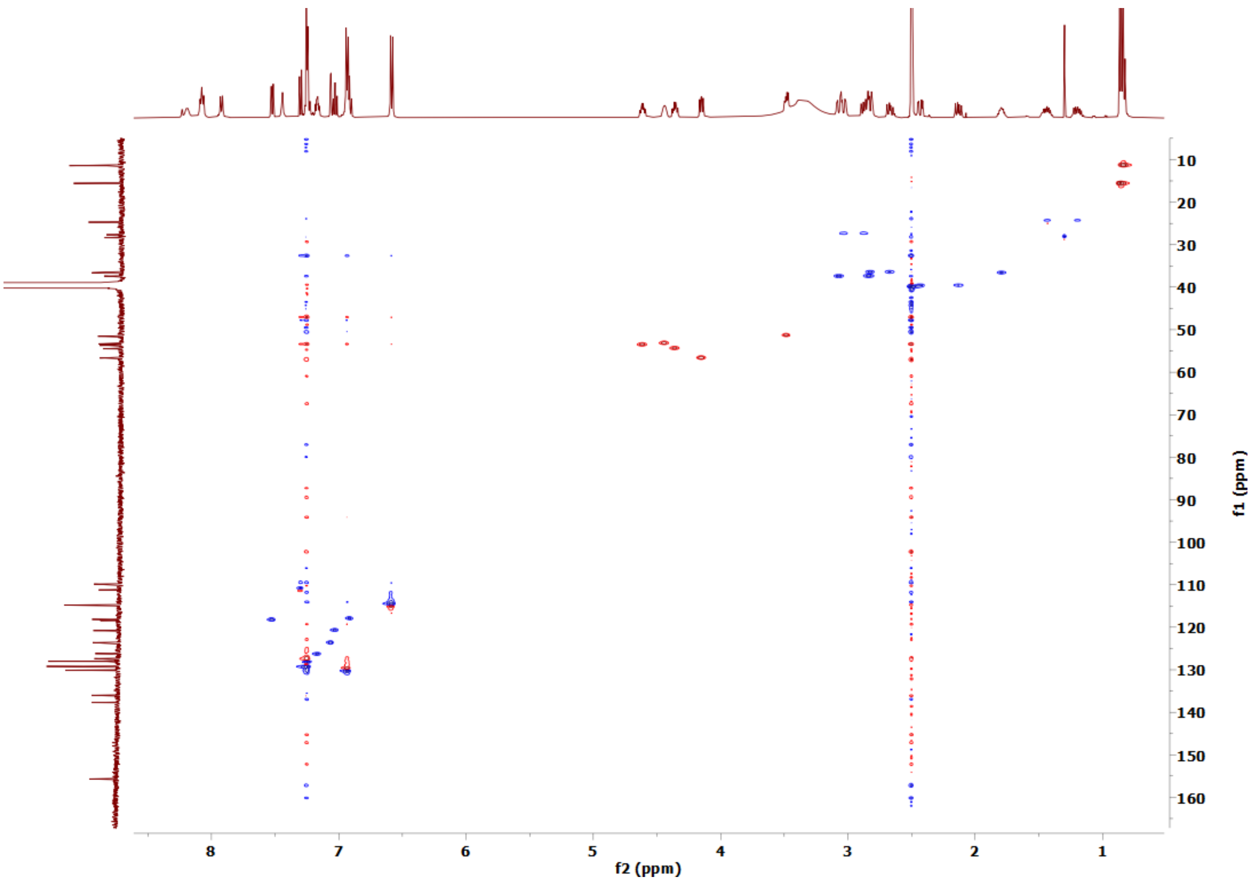

Figure S28. ^{1}H-^{13}C-HSQC NMR spectrum of IndA$_{N5\text{-}I9}$ in DMSO- d_6 (500 MHz).

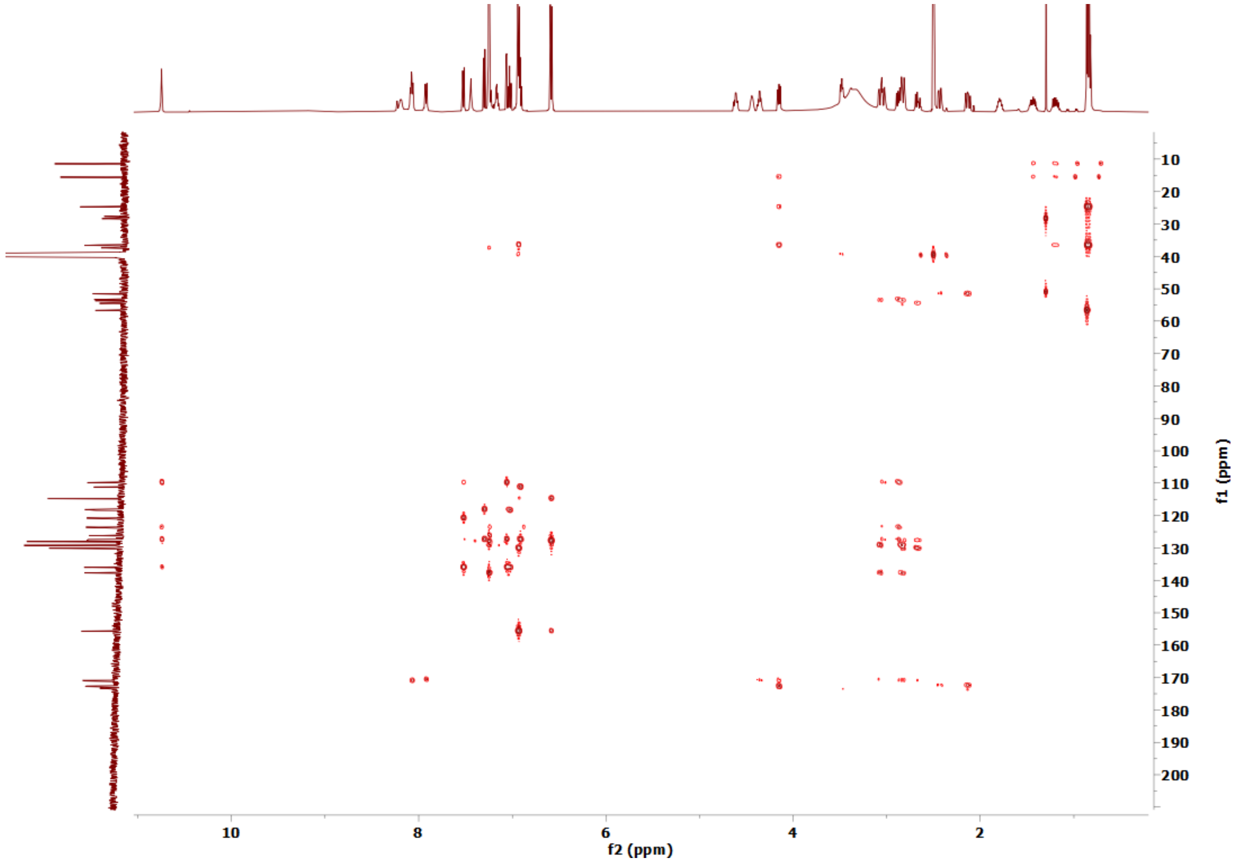

Figure S29. ^{1}H-^{13}C-HMBC NMR spectrum of $IndA_{N5\text{-}I9}$ in DMSO- d_6 (500 MHz).

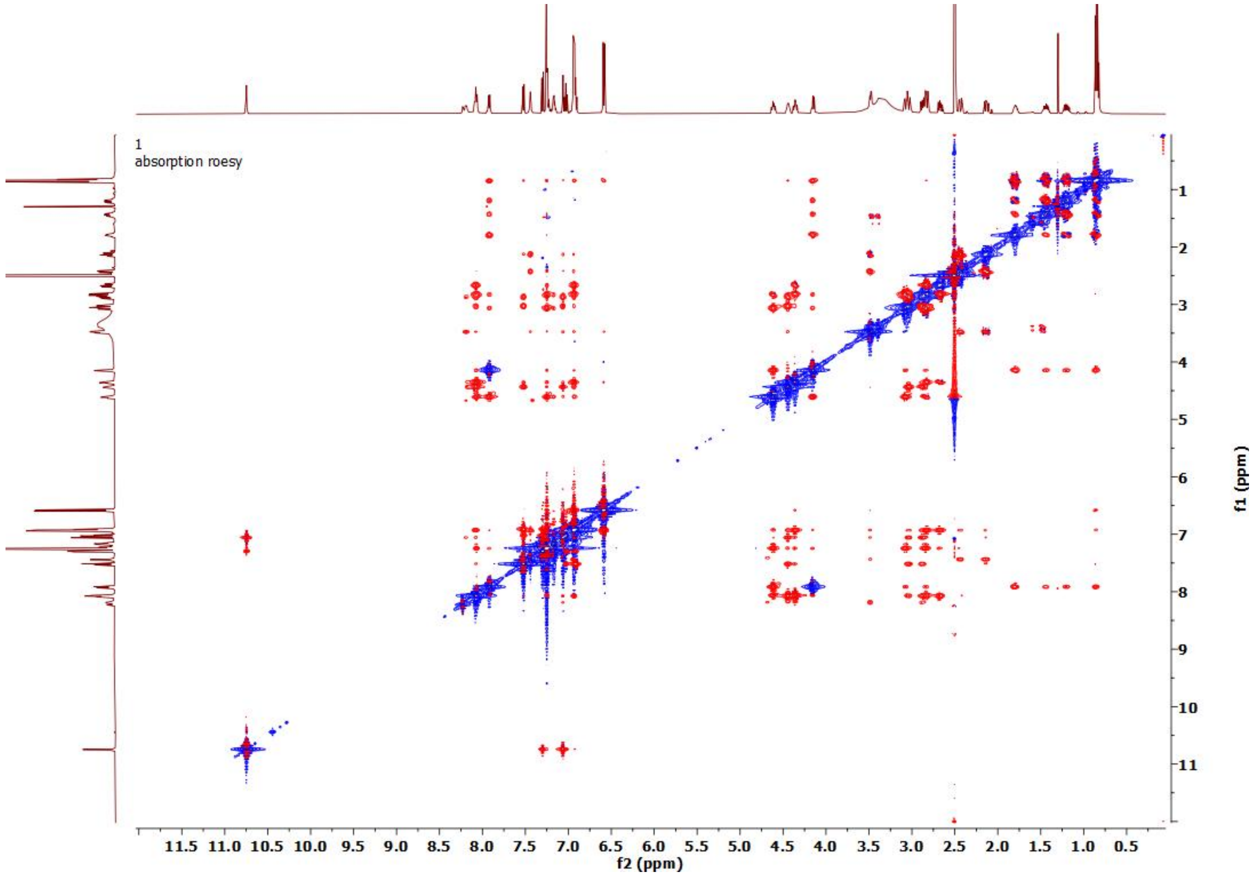

Figure S30. ^{1}H-^{1}H-ROESY NMR spectrum of IndA$_{N5\text{-}I9}$ in DMSO- d_6 (500 MHz).

Table S3. NMR table for IndA-Cyclic$_{N5\text{-}I9}$

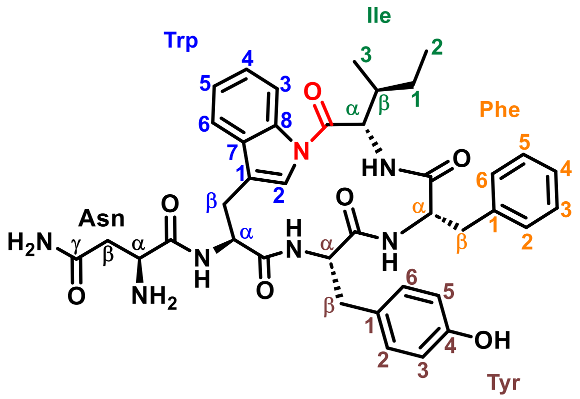

Residue	H	δ(¹H) (*J*, d) [ppm]	C	δ(¹³C) [ppm]
**Asn**	Hα	3.51-3.49 (m)	Cα	51.9
	Hβ	2.46 (d, *J* = 3.8 Hz), 2.30 (dd, J = 15.2, 8.8 Hz, 1H)	Cβ	39.9
	γ–NH	7.40 (s), 6.90 (s)		
**Trp**	Hα	4.64 (s, 1H)	Cα	51.9
	Hβ	3.05 (overlap); 2.80 (overlap)	Cβ	28.3
	-	-	C1	116.8
	H2	7.56 (s)	C2	122.4
	H6	7.80 (d, 5.9 Hz)	C3	119.0
	H4	7.32 (overlap)	C4	124.5
	H5	7.29 (overlap)	C5	123.0
	H3	8.36 (overlap)	C6	116.1
	-		C7	130.1
	-		C8	135.5
**Tyr**	Hα	4.22 (overlap)	Cα	56.2
	Hβ	2.66 – 2.54 (m)	Cβ	36.8
	-		C1	
	H2/H6	6.75 (d, *J* = 7.9 Hz)	C2, C6	129.7
	H3/H5	6.54 (d, *J* = 8.0 Hz)	C3, C5	114.8
	-		C4	155.8

**Phe**	Hα	4.37 (q, *J* = 7.9 Hz)	Cα	55.2
	Hβ	2.80 (overlap); 2.95 (dd, *J* = 13.4, 7.5 Hz, 1H)	Cβ	36.7
			C1	137.1
	H2/H6	7.21 (overlap)	C2, C6	129.
	H3/H5	7.26 (overlap)	C3, C5	127.8
	H4	7.19 (overlap)	C4	126.1
**Ile**	Hα	4.21 (overlap)	Cα	59.7
	Hβ	2.21 (m)	Cβ	34.3
	H1	1.50–1.45 (m)	C1	24.2
	H2	0.80 (overlap)	C2	10.5
	H3	0.81 (overlap)	C3	15.7
	-NH	8.93 (s)		

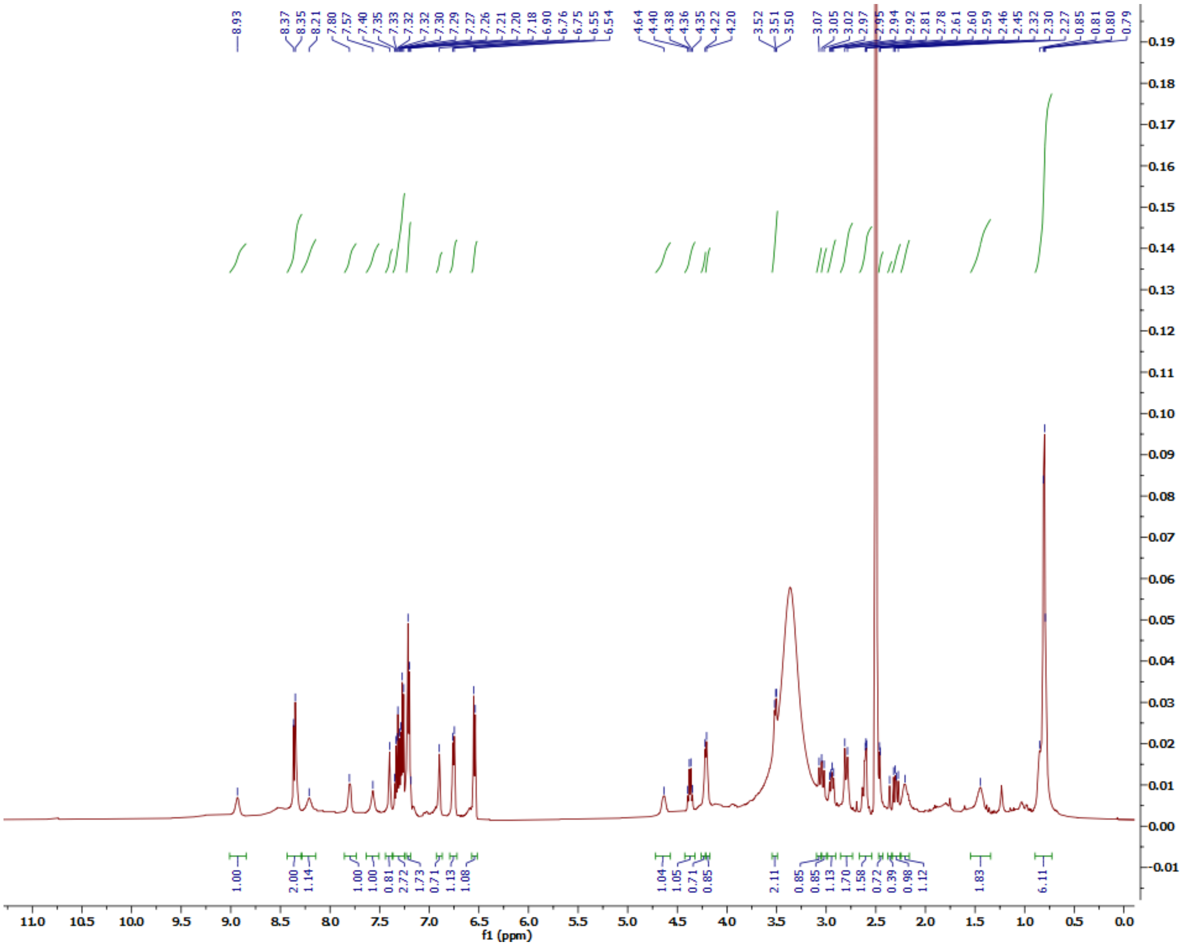

Figure S31. ^{1}H-NMR spectrum of IndA-Cyclic$_{N5\text{-}I9}$ in DMSO-d_6 (500 MHz).

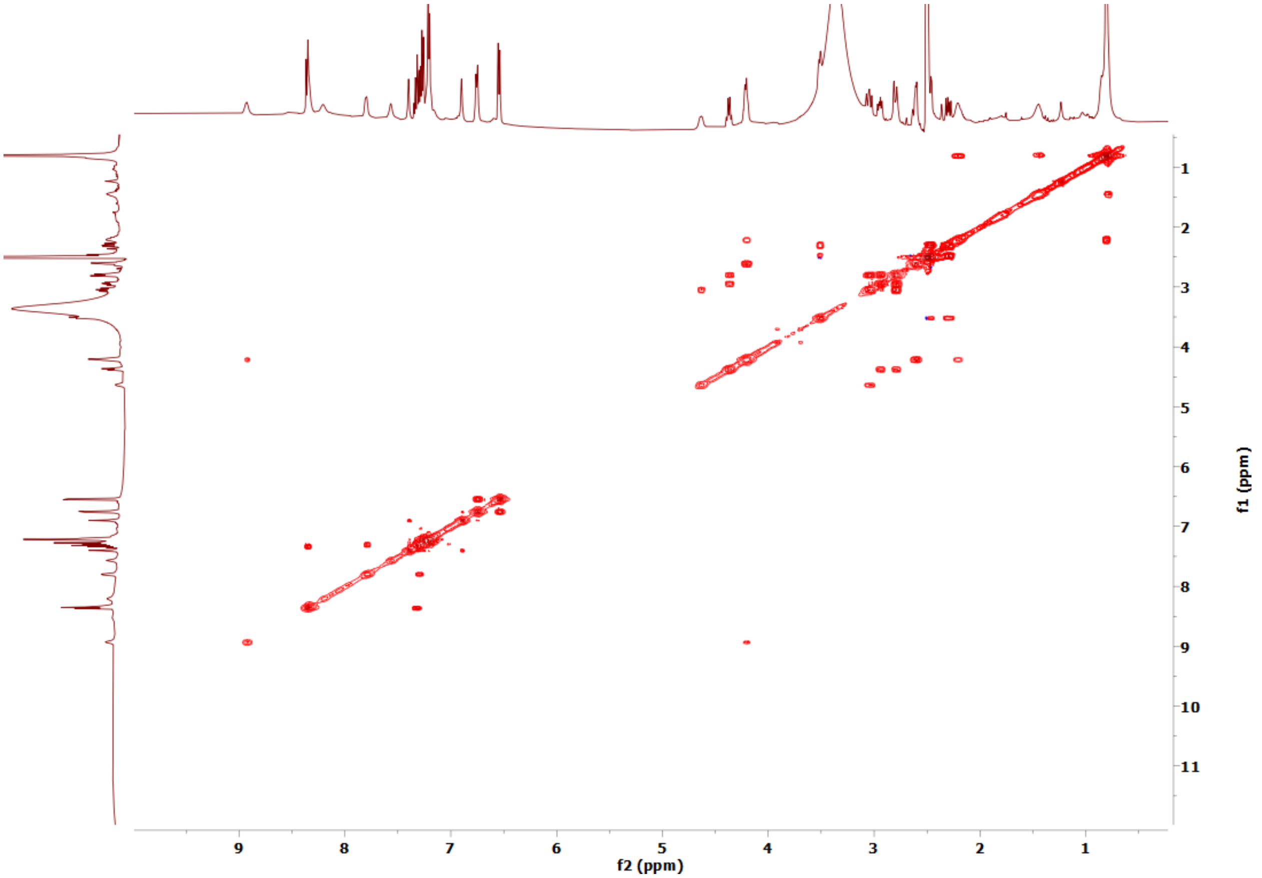

Figure S32. ^1H-^1H-COSY NMR spectrum of IndA-Cyclic$_{N5\text{-}I9}$ in DMSO-d_6 (500 MHz).

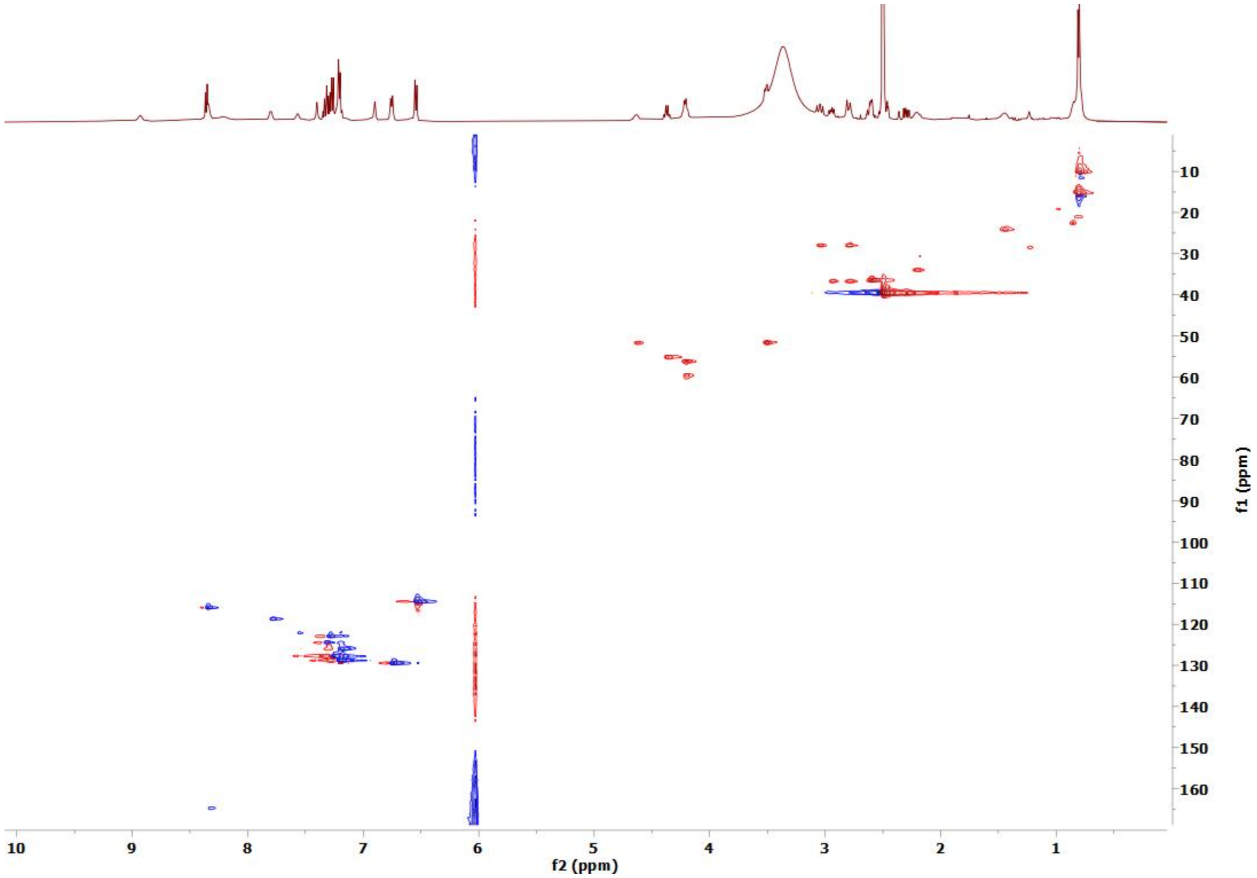

Figure S33. ^{1}H-^{13}C-HSQC NMR spectrum of IndA-Cyclic$_{N5\text{-}I9}$ in DMSO-d_6 (500 MHz).

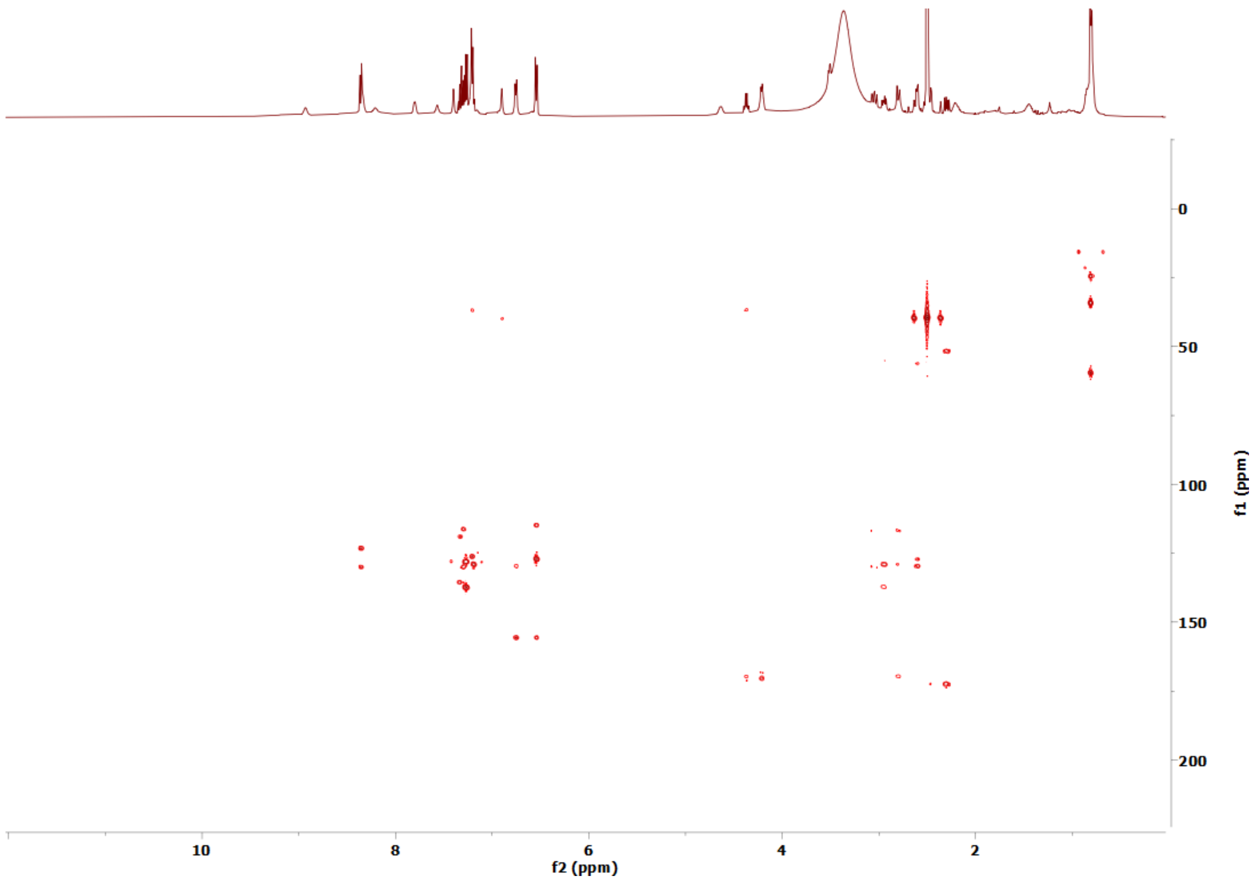

Figure S34. ^{1}H-^{13}C-HMBC NMR spectrum of IndA-Cyclic$_{N5-I9}$ in DMSO- d_6 (500 MHz).

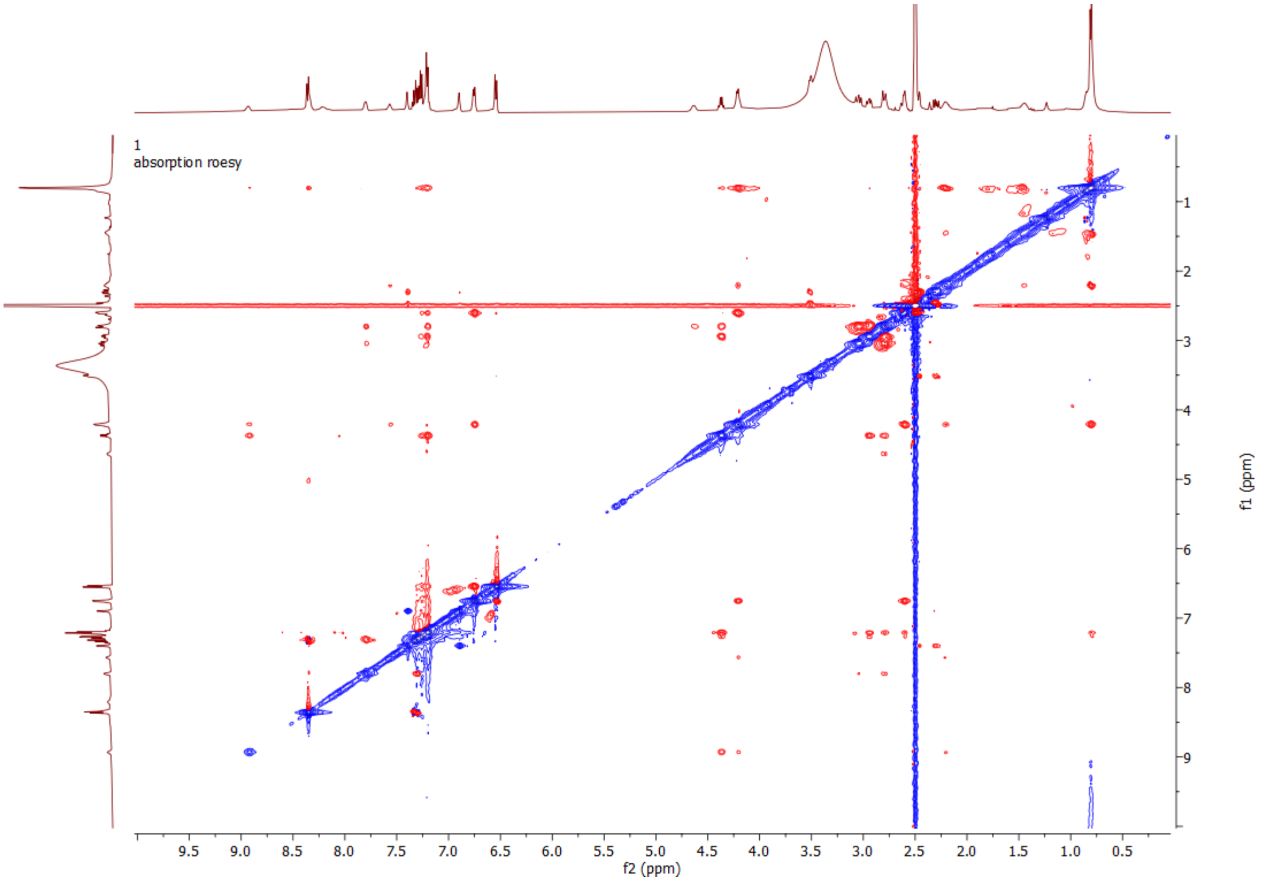

Figure S35. ^{1}H-^{1}H-ROESY NMR spectrum of IndA-Cyclic$_{N5\text{-}I9}$ in DMSO- d_6 (500 MHz).

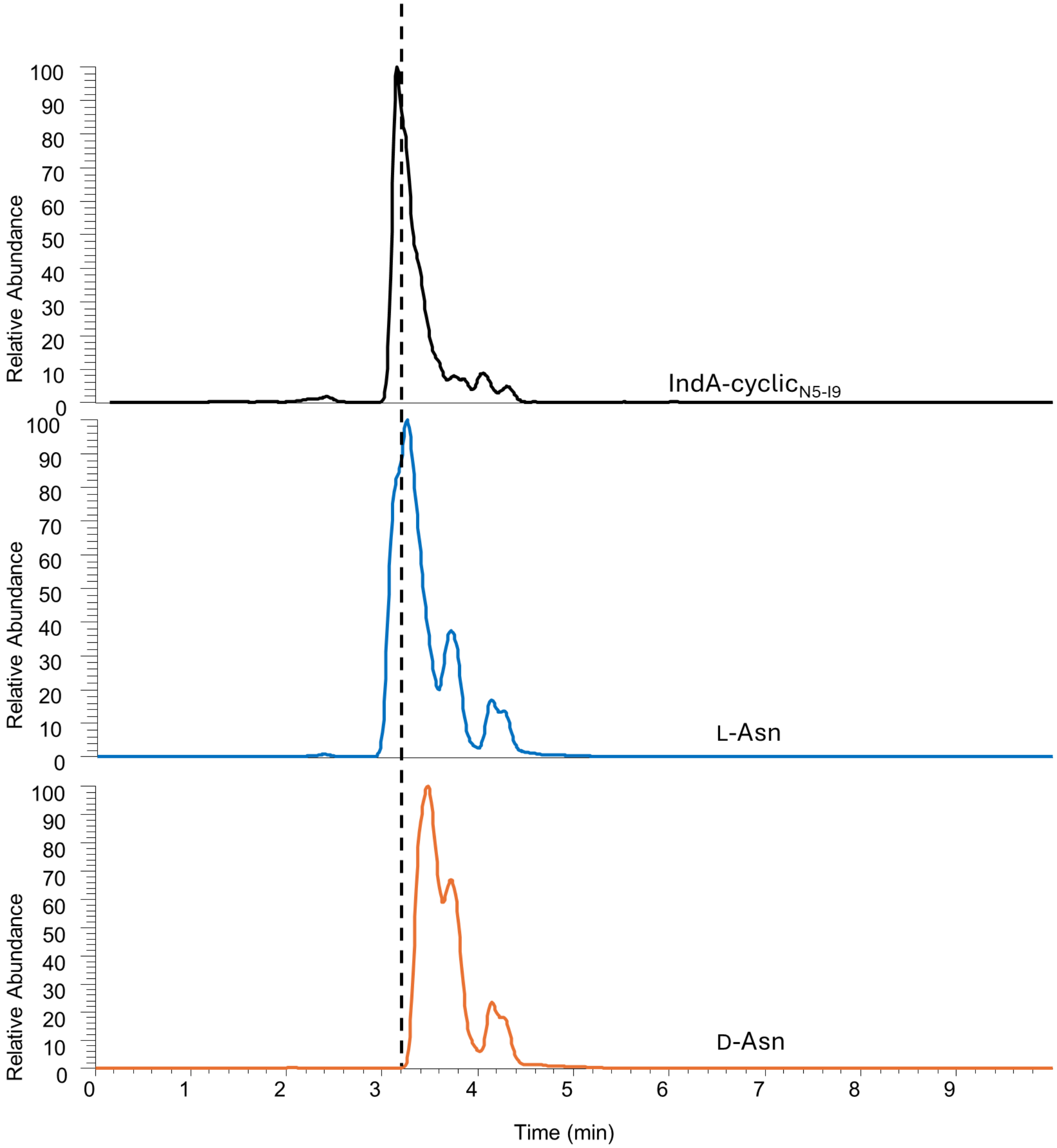

Figure S36. Extracted ion chromatogram of Marfey's analysis for Asn: hydrolyzed and derivatized IndA-Cyclic$_{N5\text{-}I9}$ compared with derivatized standards of L-Asn and D-Asn ($[M+H]^+$ 386.0943 *m/z,* mass tolerance of 50 ppm.)

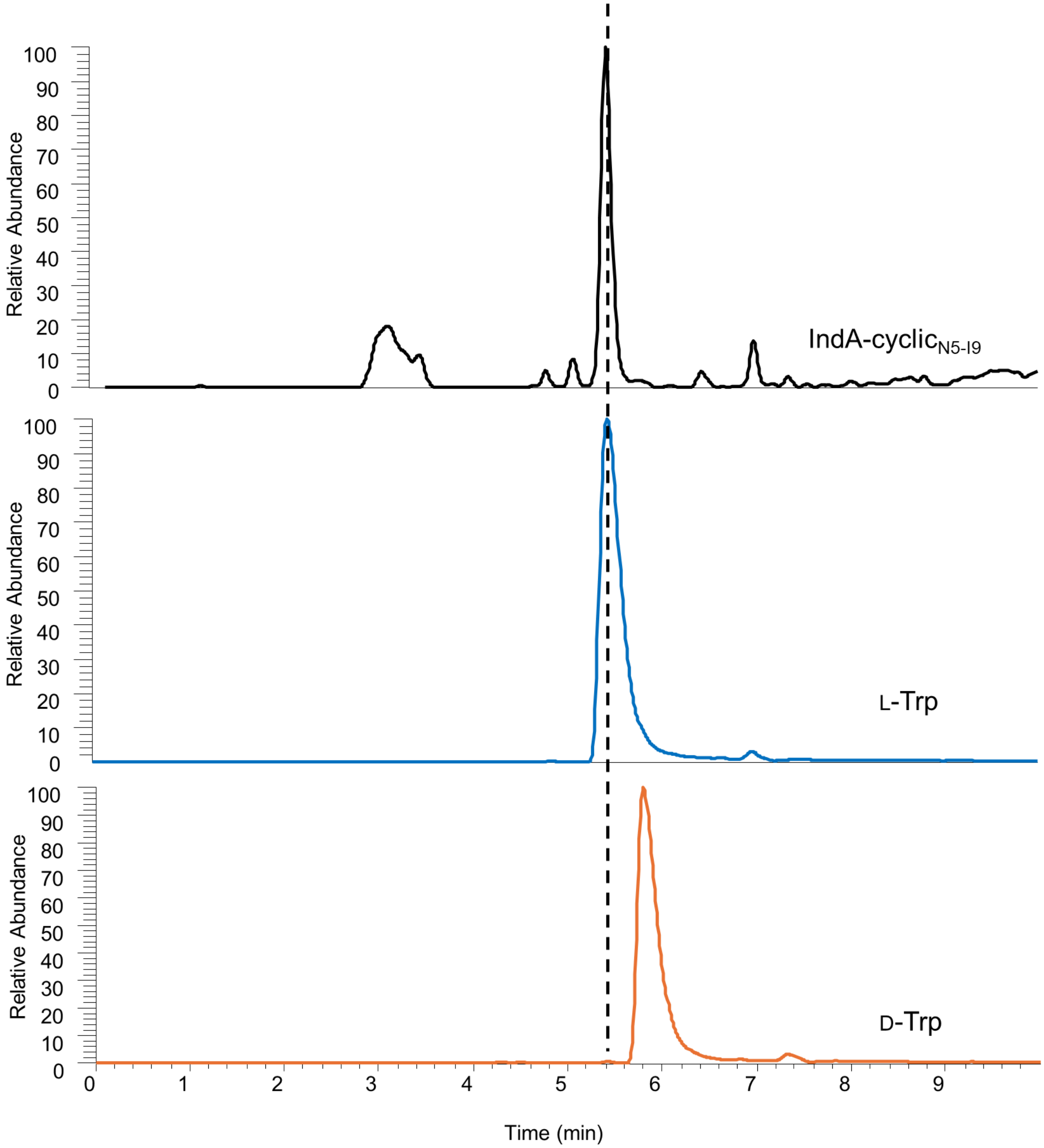

Figure S37. Extracted ion chromatogram of Marfey's analysis for Trp: hydrolyzed and derivatized IndA-Cyclic$_{N5\text{-}I9}$ compared with derivatized standards of L-Trp and D-Trp ($[M+H]^+$ 457.1466 *m/z*, mass tolerance 50 ppm).

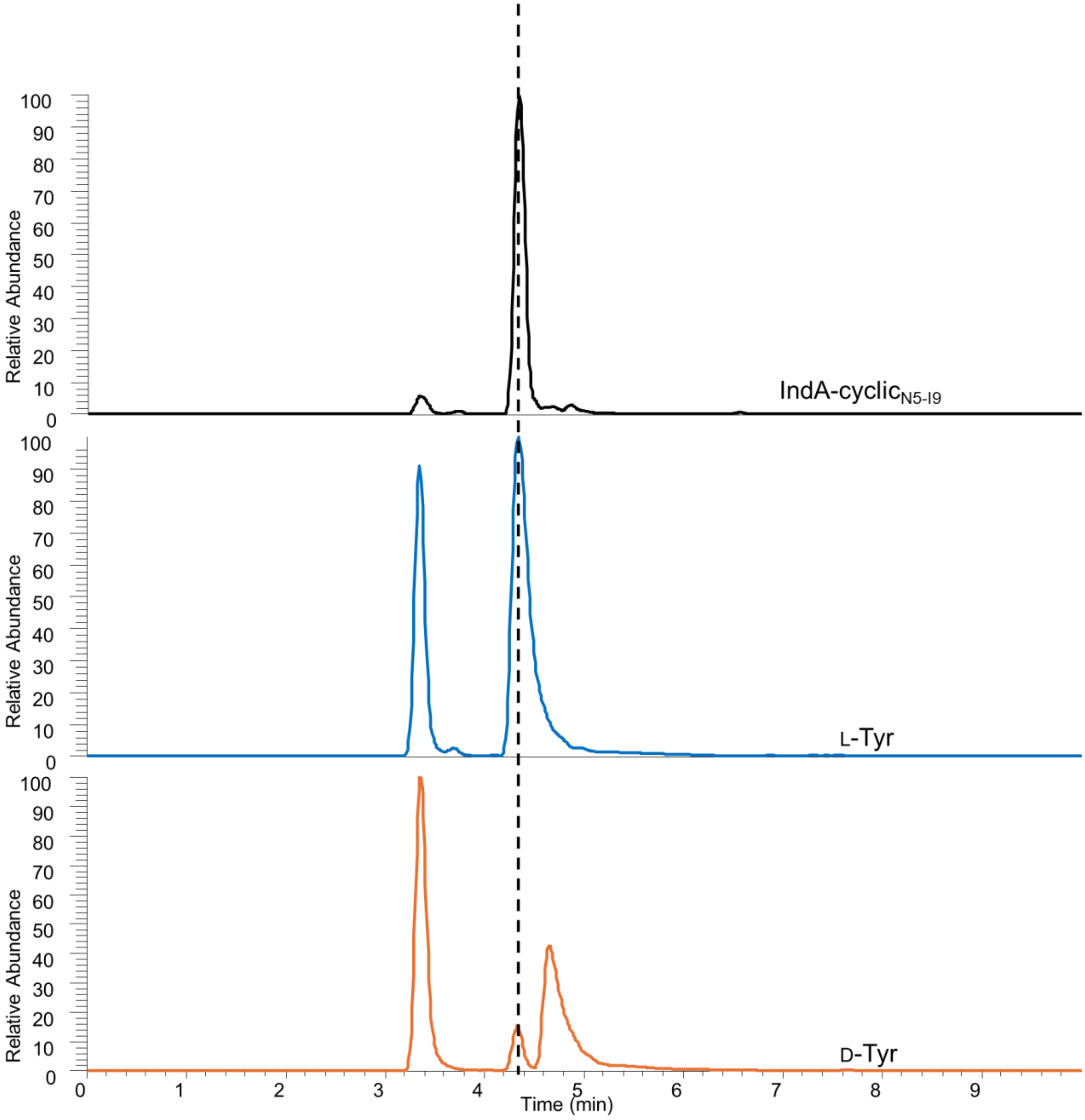

Figure S38. Extracted ion chromatogram of Marfey's analysis for Tyr: hydrolyzed and derivatized IndA-Cyclic$_{N5\text{-}I9}$ compared with derivatized standards of L-Tyr and D-Tyr ($[M+H]^+$ 434.1306 *m/z*, mass tolerance 50 ppm).

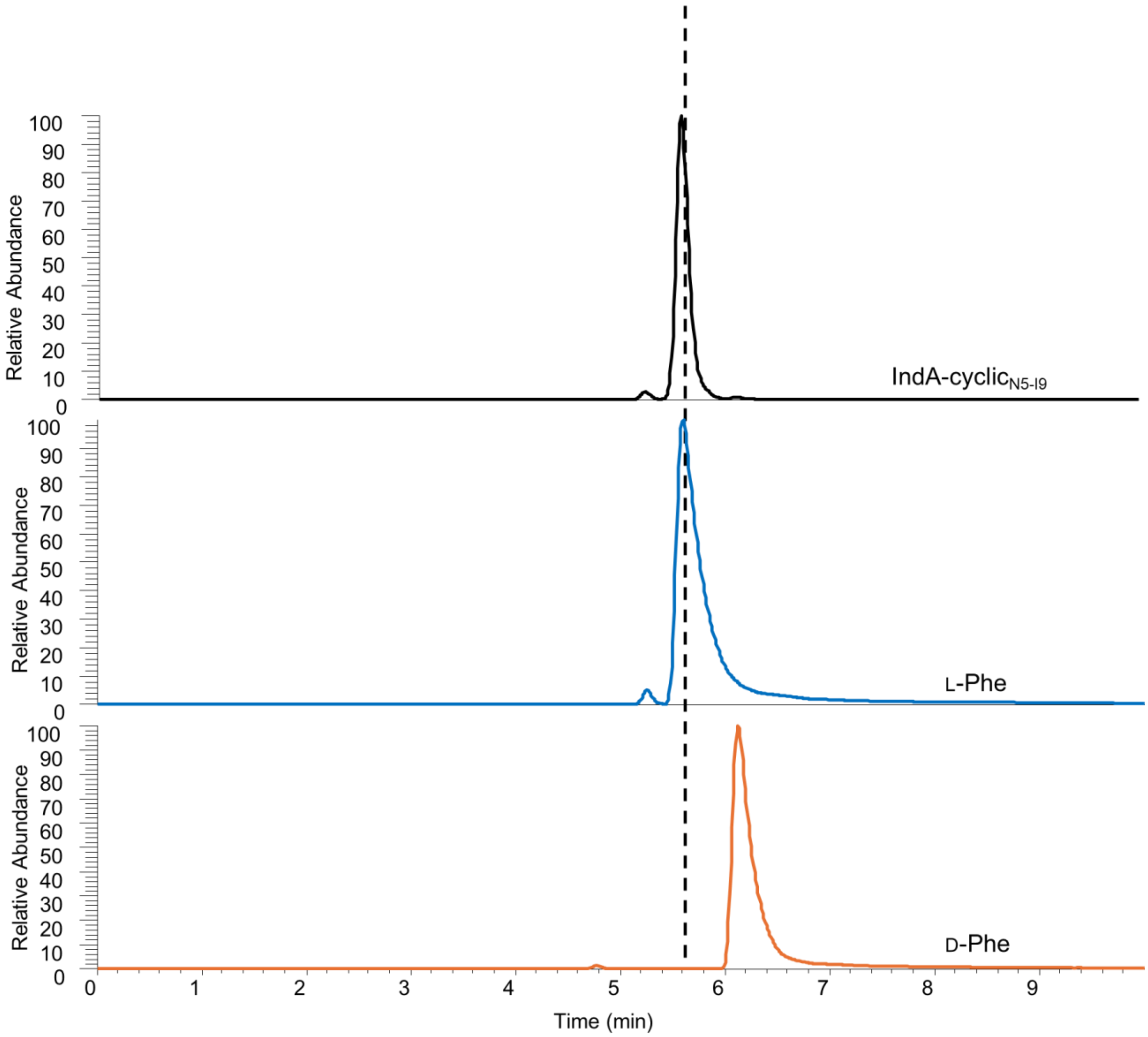

Figure S39. Extracted ion chromatogram of Marfey's analysis for Phe: hydrolyzed and derivatized IndA-Cyclic$_{N5\text{-}I9}$ compared with derivatized standards of L-Phe and D-Phe ($[M+H]^+$ 418.1357 *m/z*, mass tolerance 50 ppm).

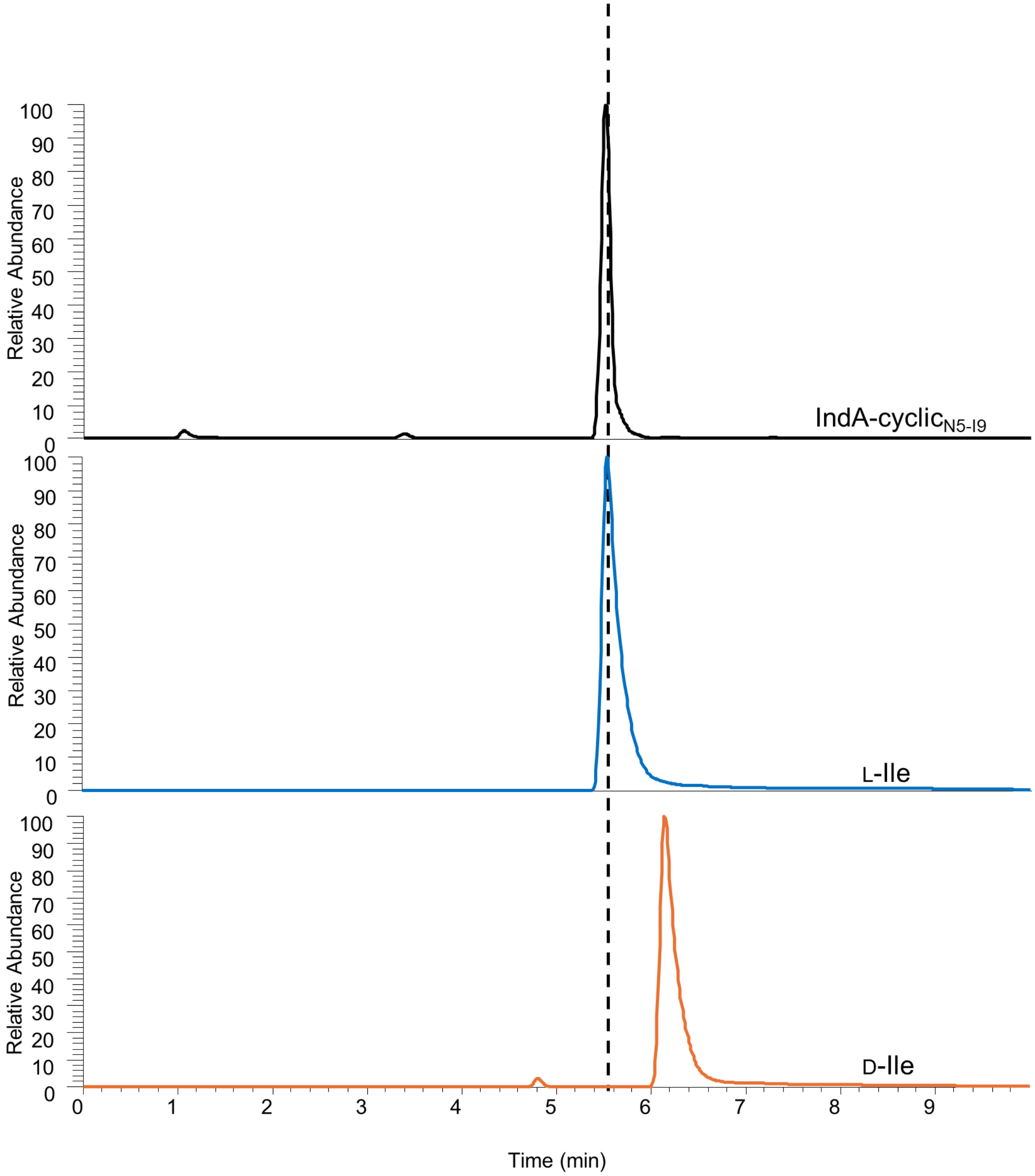

Figure S40. Extracted ion chromatogram of Marfey's analysis for Ile: hydrolyzed and derivatized IndA-Cyclic$_{N5\text{-}I9}$ compared with derivatized standards of L-Ile and D-Ile ($[M+H]^+$ 384.1514 *m/z*, mass tolerance 50 ppm).

SUPPLEMENTARY REFERENCES

(1) Novoradovsky, A.; Zhang, V.; Ghosh, M.; Hogrefe, H.; Sorge, J.; Gaasterland, T. Computational Principles of Primer Design for Site Directed Mutagenesis. *Vol* **2005**, *1*, 532–535.
